# Transcriptomic analysis of non-leukemic cell subsets in azacytidine-responsive AML highlights pathways associated with adhesion, platelet aggregation, and angiogenesis in mice and humans

**DOI:** 10.1186/s10020-025-01233-2

**Published:** 2025-05-13

**Authors:** Nancy D. Ebelt, Suvithanandhini Loganathan, Lara C. Avsharian, Edwin R. Manuel

**Affiliations:** 1 Department of Immuno-Oncology, Beckman Research Institute of the City of Hope, Duarte, CA USA; 2 Irell and Manella Graduate School of Biological Sciences, City of Hope, Duarte, CA USA

**Keywords:** Acute myeloid leukemia, 5-azacytidine, Hypomethylating agents, Remission, Relapse, Transcriptomics, Platelet coagulation, Integrin-mediated adhesion

## Abstract

**Background:**

Hypomethylating agents (HMAs), such as 5-azacytidine (AZA), are valuable treatment options for patients with acute myeloid leukemia (AML). Despite providing significant extensions in survival when used alone or in combination with BCL-2 inhibitors, resistance and eventual relapse is observed. Reported mechanisms of these outcomes are inconsistent when focusing on leukemic populations within bone marrow, indicating a need for studies on the impact of HMAs on non-leukemic cells in the blood and other tissue compartments.

**Methods:**

Whole blood and spleens from vehicle- or AZA-treated mice implanted with the syngeneic AML line C1498 were transcriptionally profiled using a comprehensive panel of immune-related gene probes. Publicly available RNAseq data from blood of AZA-responsive, human AML patients were analyzed compared to matched, pre-treatment samples. Genes differentially expressed between vehicle- and AZA-treated (mouse) or pre- and post-AZA treatment (human) samples were analyzed for statistical overrepresentation in gene ontologies using Fisher’s one-tailed *t*-test. Pathological analyses of various tissues in AML relapsed, AZA-responsive mice were compared to the corresponding tissues in vehicle-treated mice.

**Results:**

We observed hematologic recovery in the peripheral blood of AZA-treated groups, versus vehicle control, that was associated with significant extensions in survival. Transcriptional analysis of AZA-treated samples revealed decreased cell type scores for suppressive subsets and increased pathway scores for T and B cell functions. Comparisons of gene ontology annotations enriched from genes differentially regulated by AZA in human and mouse blood samples revealed overlap in numerous biological pathways including adhesion, thrombosis, and angiogenesis. Consistently, C1498 permeated the liver at end-stage disease in vehicle-treated mice, while AZA treatment limited their spread to just outside the bone after relapse.

**Conclusions:**

AZA-induced differences in C1498 spread could be a result of gene expression changes in adhesion, platelet aggregation and/or angiogenesis in non-leukemic compartments; however, further mechanistic studies must be done to confirm a direct link between modulated genes and disease manifestation. Overall, these studies provide rationale for expanding the exploration of biomarkers and therapeutic targets to include normal immune cells in blood, spleen, or other microenvironments of AML patients treated with HMA, rather than limiting studies to the bone marrow and leukemic blasts.

**Supplementary Information:**

The online version contains supplementary material available at 10.1186/s10020-025-01233-2.

## Background

Patients with acute myeloid leukemia (AML) are frequently ineligible for standard of care chemotherapy and hematopoietic stem cell (HSC) transplantation due to extensive co-morbidities (Abdel-Wahab et al. 2012;, Abuelgasim et al. 2020). In these cases, hypomethylating agents (HMA) such as 5-azacytidine (AZA) or decitabine are well tolerated and show overall response rates as high as 50% (Alexandraki and Strati 2022;, Anderson et al. 2019). The correlation of BCL-2 expression in AML patients with poorer overall survival (Antar et al. 2013;, Bankova et al. 2021)and the pre-clinical and clinical discoveries of synergy between BCL-2 inhibition and HMAs (Bohl et al. 2013;, Brenner et al. 2017)have resulted in the approval of AZA and venetoclax (a small molecule inhibitor or BCL-2) for the treatment of AML in 2017. This combination therapy increases overall response rates (66%) and survival significantly (median follow up 20.5 months) (Chang 2020). Despite these advances, many patients that respond to the combination treatment will relapse in ≥ 3 years, with overall survival reaching only 35–40% (Chen et al. 2021), similar to AZA monotherapy (2-year survival ~ 50%) (Alexandraki and Strati 2022). Determining the direct, cause-effect relationship between HMAs and anti-leukemia efficacy will be crucial in identifying novel treatment combinations to increase long-term remission for AML patients.

Studies of the precise mechanisms of HMA action have utilized methylation array, gene expression array, and RNAseq, but only of sorted blasts or blast-heavy bone marrow aspirates, despite the fact that HMA efficacy has a large immune component involving the action of non-cancerous white blood cells (WBC) (Cheng et al. 2018; –Ebelt et al. 2020). Thus, these studies have reported highly conflicting results with regard to methylation and gene expression signatures found, and although some signatures had prognostic significance, few were able to help narrow down a common mechanism for HMA efficacy (Ebelt 2022; –Fraczkowska et al. 2018). These inconsistencies form the rationale for performing additional studies focusing on the non-bone marrow AML microenvironments and constituents in addition to the blasts themselves.

For the current study, we utilize the C1498 AML cell line that arose spontaneously in a C57Bl/6 mouse. Intravenous administration of C1498 results in infiltration of the cell line into bone marrow where mono-and myeloblastic cells proliferate to cause bone marrow failure. C1498 also colonize other compartments such as the blood, spleen, liver, lungs and lymph nodes (Cheng et al. 2018;, Gañán-Gómez 2015;, Genomic 2013). This model recapitulates the progression and end stage disease of human AML. Using this model, we have focused on gene expression alterations, following AML response to AZA treatment, in non-leukemic cells of mouse peripheral blood and spleen that may offer insights into mechanisms causing eventual relapse and altered disease outcomes in AML. We compared our results with transcripts from peripheral blood of human AML patients responsive to AZA treatment. These comparisons revealed extensive overlap in multiple biological processes and pathways between mouse and human that can be further explored to produce rational treatment combinations that enhance AZA efficacy and further prevent AML progression and relapse.

## Methods

### Animals and cell lines

C57Bl/6 mice were obtained from breeding colonies at the City of Hope Animal Research Center. NOD.Cg-*Prkdc*^*scid*^* Il2rg*^*tm1 Wjl*^/SzJ (NSG) mice were obtained from Jackson Labs (catalog# 005557). Mice were handled according to institutional animal care and use committee guidelines (protocol #17128). The C1498 mouse AML cell line was obtained from ATCC® (TIB-49, Manassas, VA, USA). Cell lines were maintained in RPMI media supplemented with 10% FBS, 2 mM L-glutamine and 100 units/mL penicillin, and 100 ug/mL streptomycin. Cell lines were passaged minimally (≤ 5 times) before implantation in mice.

### *In Vivo *AZA treatment studies and survival studies following leukemia challenge

Six- to eight-week old male mice (averaging 22 g) from C57Bl/6 or NSG backgrounds were implanted with 8 × 10 (Anderson et al. 2019) C1498 via intravenous tail-vein injection. C1498 are syngeneic to C57Bl/6. Three days post-implant, mice were randomized into two experimental groups and treated with 5 mg/kg AZA or vehicle (DMSO in diluent) for 3 consecutive days by intraperitoneal injection in a total volume of 500 µl in 1 × HBSS. Mice showing signs of terminal disease following leukemic challenge (lethargy, paralysis, hunched posture, etc.) were immediately euthanized. Survival data is displayed as Kaplan–Meier curves. Numbers of animals (n) are reported per experiment in the figure legends. Treatments occurred unblinded but raw data from the bulk RNA analysis were collected by a third party company and analyzed with their software according to their guidelines (see below).

### Complete blood counts

Whole blood was sampled from the retroorbital vein using heparinized microhematocrit capillary tubes (22–362–566, Fisherbrand; Waltham, MA, USA) and smeared onto lysine-coated glass slides. Blood smears were dried, fixed in methanol, and stained with Wright-Giemsa (WG32, Sigma-Aldrich; St. Loius, MO, USA). CBC were conducted manually using a light microscope. Platelet counts and morphology observations were made from images taken on the Widefield Observer 7 (Zeiss; Oberkochen, Germany) microscope using the 100 × lens with oil. The AxioCam 506 Color camera (Zeiss) was used to capture images. Images were analyzed using ZEN Blue 3.0 software (Zeiss).

### Statistics

All statistical analyses were performed using Prism software by GraphPad (V9) (San Deigo, CA, USA). Types of tests used are reported in figure legends per experiment. Unless otherwise indicated, all error bars represent standard error of the mean.

### Network topology analysis of mouse PanCancer immune profiling panel

The full gene list, unranked, included in the Mouse PanCancer Immune Profiling Panel was entered into the WEB-based Gene Set Analysis Toolkit (WebGestalt.org) (Gruszka 2019) for Network-Topology-based Analysis. Organism:*Mus musculus*; Functional Database: PPI BIOGRID.

### Bulk RNA gene expression analysis for mouse blood and spleen

RNA Extraction and QC: RNA was extracted from whole blood and spleen using the RNeasy Plus Mini kit (74134, Qiagen; Hilden, Germany) and quantified using the Qubit RNA high-sensitivity kit (Q32852, ThermoFisher; Waltham, MA, USA) and Qubit 4 Fluorometer (ThermoFisher). Size distribution was measured using Bioanalyzer 2100 (Agilent; Santa Clara, CA, USA).

nCounter Bulk RNA Expression Assay: The CodeSet hybridization included 8 µl of total RNA (200 ng); 10 μl hybridization master mix containing 5ul of hybridization buffer, 3 µl of Reporter CodeSet and 2 µl of Reporter Plus CodeSet; and capture master mix (100052, Nanostring; Seattle, WA, USA) containing 2 µl of Capture Probe Set and 1 µl of Capture Plus Probe Set. The CodeSet of Mouse PanCancer Immune Profiling Panel (XT-CSO-MIP1-12, Nanostring) with Panel Plus (20 additional genes, Table S1) was used for this study (15,000,142, Nanostring). After overnight hybridization at 65 °C for 18 h, samples were processed, and data acquisition was performed by nCounter MAX/FLEX profiler (Nanostring).

### Differential gene expression, cell type score, and pathway score analysis of mouse gene expression data

nCounter bulk RNA expression data were analyzed using nSolver Analysis Software (Version 4.0, R Version 3.3.2) with the nSolver Advanced Analysis Add-On (Nanostring).

### Gene enrichment analysis

Unranked, unordered gene lists were uploaded to https://pantherdb.org/. Annotated genes were queried for statistical overrepresentation in annotation sets GO biological process complete, GO cellular component complete, GO molecular function complete and PANTHER pathways using Fisher’s one-tailed t-test. Venn diagrams of terms were created using BioVenn (Hankenson et al. 2011).

### Differential gene expression analysis of human data

The Gene Expression Omnibus (GEO) dataset GSE118558 (Haouas 2014)was analyzed using GEO2R (Harris et al. 2012;, Hulsen et al. 2008) (National Center for Biotechnology Information, a division of the National Library of Medicine at the National Institutes of Health (NIH), Bethesda, MA, USA). Samples GSM3333205 and GSM3333197 were used for AZA-treatment responsive samples compared to GSM3333202 and GSM3333194 pre-treatment samples. GEO2R uses the Wald test followed by adjustment for multiple hypothesis testing using the Benjamini–Hochberg method (alpha cut-off 0.05).

### Data sharing statement

Human datasets are publicly available using the accession numbers above. Mouse bulk RNA raw data files are uploaded to GEO under series GSE283410 (access token: sncdqmukzfinhkh).

## Results

### Prolonged AZA treatment extends survival and promotes hematologic recovery in leukemic mice

**Fig. 1:**
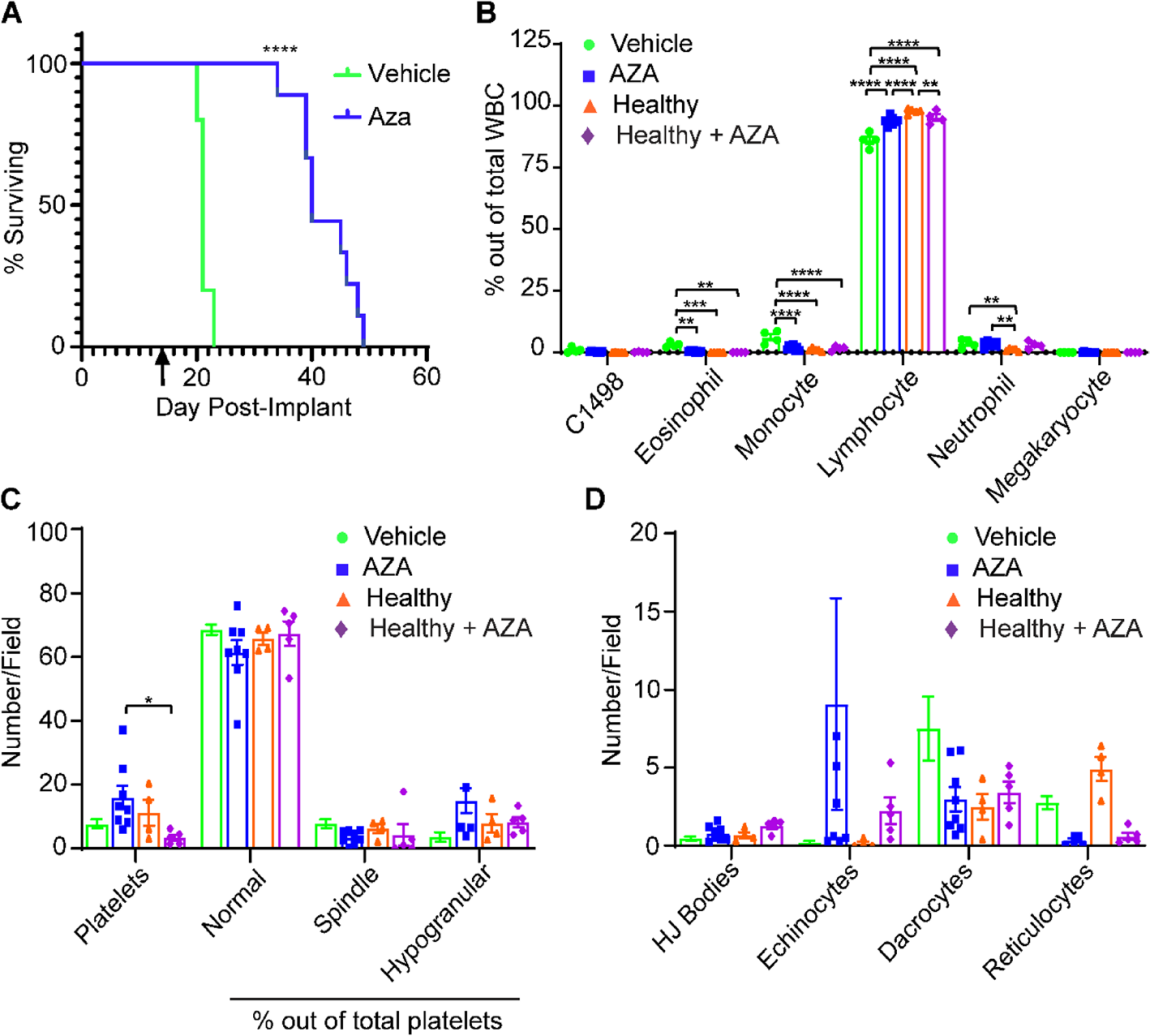
Survival and Hematologic Profiles of Leukemic and Non-Leukemic Vehicle- and AZA-treated Immunocompetent Mice. C1498 leukemia cells (80,000) were implanted in C57Bl/6 mice via tail vein injection. Treatment with DMSO in diluent (Vehicle) (*n* = 5) or 5 mg/kg/mouse AZA (*n* = 11) by intraperitoneal injection began on day 3 after implantation and continued thrice weekly until survival endpoints. **A** Mice were euthanized once they showed signs of terminal disease state and survival was recorded for each mouse and displayed as a Kaplan–Meier curve. Statistical significance was analyzed using the Log-rank (Mantel-Cox) test: ****p < 0.0001. The arrow (↑) indicates the timepoint at which retroorbital blood was drawn to assess hematologic profiles. **B** Blood drawn at week 2 after treatments was smeared and stained for manual complete blood counts. Blood from non-leukemic, untreated (Healthy) mice and non-leukemic, AZA-treated (Healthy-Aza) was also included. White blood cells (WBC) were counted manually, including C1498, and are presented as a percentage of total WBC counted. **C** Numbers of platelets and percentages of normal, spindle-shaped, and hypogranular platelet morphologies out of total platelets are presented. **D** Red blood cell morphologies including the presence of Howell-Jolly bodies (HJ Bodies), echinocytes, dacrocytes, and reticulocytes are presented as numbers counted per field. Statistical significance was analyzed using 2-way ANOVA followed by Tukey’s test for multiple comparisons: *p < 0.05, **p < 0.01, ***p < 0.001, ****p < 0.0001

In our previous study, a single round of AZA treatments (three doses in one week) using the C1498 mouse AML model only resulted in a modest increase in survival, probably indicating that a majority of blasts were still present(Cheng et al. 2018). To ensure full penetrance of AZA treatment to more resistant clones, we continuously treated C1498-implanted C57Bl/6 mice thrice weekly with AZA until survival endpoints were reached. C1498-implanted, AZA-treated mice (C1498-AZA) survived significantly longer than C1498-implanted, DMSO-treated mice (C1498-vehicle) (Fig. 1A, *p*< 0.0001, Mantel-Cox test, median survival 40 vs. 21 days, respectively). These data are consistent with human AML patients experiencing increased benefit from prolonged AZA treatment beyond initial response (Jeyaraju et al. 2024).

In our previous study, we also observed that immunodeficient NSG mice implanted with C1498 and treated with a single course of AZA did not survive significantly longer than vehicle-treated mice (Cheng et al. 2018). We repeated this experiment with continuous AZA treatment, as in Fig. 1A, and found that survival of C1498-AZA NSG mice did not differ significantly from C1498-vehicle NSG mice (Figure S1 A, *p* = 0.0816, Mantel-Cox test, median survival 38.5 vs. 23 days, respectively).

Hematologic recovery of blood cell types is an important indicator of treatment efficacy and AML remission (Karakas et al. 1998). As it takes approximately three weeks for a minimal number of actively growing C1498 blasts to cause end stage AML, we interpolated that mice should be in remission/peak AZA efficacy after two consecutive weeks of treatment. Peripheral blood was drawn 48 h after the last treatment and complete blood counts (CBC) were conducted (Fig. 1B). At this timepoint, few C1498 blasts were observed in C1498-AZA and C1498-vehicle mice. C1498-AZA blood showed significantly decreased percentages of eosinophils and monocytes and an increased percentage of lymphocytes compared to C1498-vehicle mice. However, the percentages of neutrophils remained significantly higher, with lymphocytes concurrently lower, in C1498-AZA mice compared to healthy mice. Some of these effects may be due the action of AZA independent of the presence of C1498, as healthy mice treated with AZA (Healthy-AZA) showed decreased lymphocytes compared to healthy mice; however, percentages of neutrophils in healthy-AZA mice remained consistent with healthy mice (Fig. 1B). These results possibly indicate a mechanism of AZA efficacy or an indication of incomplete hematologic recovery at this timepoint. Interestingly, week 2 blood smears from C1498-AZA NSG mice were almost completely devoid of WBC. In C1498-vehicle NSG mice, blood was populated mostly by monocytes and neutrophils (Figure S1B) as is to be expected in this immunodeficient strain (Katagiri 2017). These data may indicate that AZA treatment removes immunosuppressive, myeloid cell types, but a lack of functional lymphocytes in NSG mice impedes the clearance of C1498 leukemia cells.

Total platelets did not differ significantly between C1498-vehicle, C1498-AZA, or healthy mice but total platelets were significantly lower in healthy-AZA mice compared to leukemic mice treated with AZA (Fig. 1C), this highlights another effect that is specific to AZA treatment in the context of leukemia. Platelets in all groups were largely of normal morphology (Fig. 1C). Although not significant, several C1498-AZA blood smears showed a high number of echinocytes (Fig. 1D). Because they are also found in high numbers in healthy-AZA mice, this is likely due to a direct effect of AZA treatment on RBCs as echinocytes can be found after exposure to other chemotherapeutic agents (Keating 2012;, Kobayashi 2020). Overall, these data suggest that therapeutic doses of AZA, leading to remission in leukemic mice, re-establishes profiles resembling a healthy blood microenvironment.

### AZA treatment generates an anti-leukemic immune transcript profile in blood and decreases overall immune transcripts in the spleen

Because the bone marrow microenvironment is key in leukemogenesis and AML progression, human datasets comprised of bone marrow mononuclear cells (typically > 80% leukemic blasts) before and after HMA treatment have been analyzed for biomarkers of HMA efficacy, but no significant gene expression changes have been found (Konopleva and Letai 2018)(GSE77750 AML samples only, GSE116567). Studies of non-leukemic blood and spleen cells in human AML patients have been largely neglected despite the importance of normal immune cells in AZA-mediated cancer control (Cheng et al. 2018; –Ebelt et al. 2020).

To delve more deeply into the phenotypes of normal immune cells and other blood components in peripheral blood and spleens in our model, RNA isolated from whole blood or whole spleen samples from C1498-vehicle, C1498-AZA, or healthy, immunocompetent mice was assayed for gene expression using a panel of 543 immune-related genes. The genes in this panel focused on cell activation, cytokine production, adaptive immune response, immune effector processes, activation and regulation of the immune response, and leukocyte differentiation (Figure S2).

**Fig. 2:**
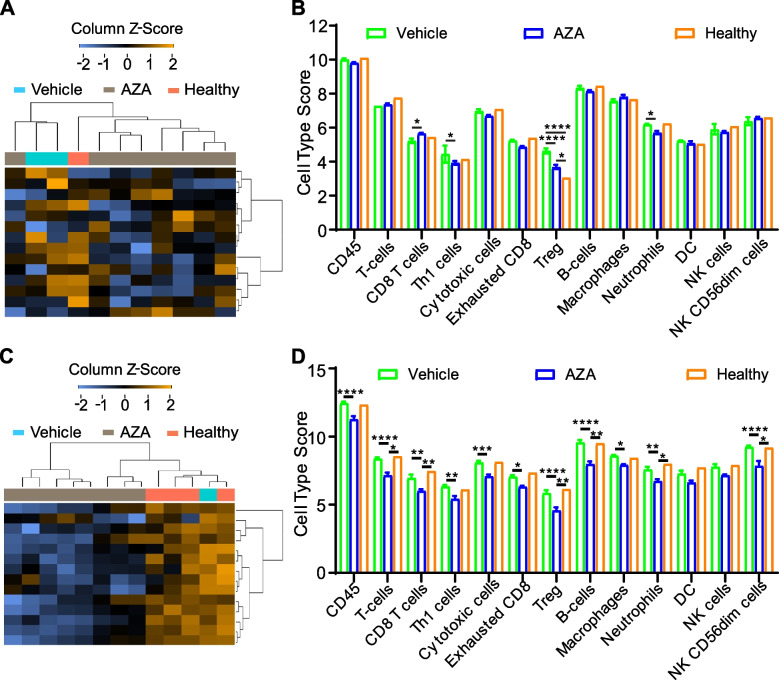
In-depth Analysis of Immune Cell Abundance and Phenotypes by Gene Expression. C1498 (8 × 10^4^ cells) were implanted in C57Bl/6 mice via tail vein injection. Treatment with DMSO in diluent (Vehicle) (*n* = 4) or 5 mg/kg/mouse AZA (*n* = 8) by intraperitoneal injection began on day 3 after implantation and continued thrice weekly. Forty-eight hours after the 6.^th^ treatment (week 2) mice were euthanized after harvesting whole blood and whole spleens. Whole blood and spleen were also isolated from a non-leukemic, untreated (Healthy) mouse. mRNA was isolated from blood and spleen and probed for gene expression using the Mouse PanCancer Immune Profiling Panel. Cell type scores were calculated from bulk mRNA expression per sample and are presented as heatmaps and bar graphs for both blood (**A**, **B**) and spleen (**C**, **D**). Statistical significance was analyzed using 2-way ANOVA followed by Tukey’s test for multiple comparisons: *p < 0.05, **p < 0.01, ***p < 0.001, ****p < 0.0001

Peripheral blood cell types were enumerated by manual CBC in Fig. 1B, however deconvolution of cell types from bulk RNA provides transcriptomic differences in WBC phenotypes (Fig. 2). Hierarchical clustering of blood samples reveals that C1498-AZA cell type scores typically group with Healthy cell type scores, while C1498-vehicle scores cluster separately (Fig. 2A). When comparing specific cell type scores, C1498-AZA blood had significantly increased CD8 + T-cell, decreased Th1 cell, decreased regulatory T-cell (Treg), and decreased neutrophil scores compared to C1498-vehicle blood (Fig. 2B). In spleens, Healthy and C1498-vehicle samples cluster together overall regarding cell type scores (Fig. 2C). C1498-AZA mice had significantly lower scores for most cell types analyzed including CD45, T, CD8 + T, Th1, cytotoxic, exhausted CD8 +, Treg, B, macrophages, neutrophils, and CD56 dim NK cells (Fig. 2D). This is likely due to overall low reads for all AZA samples in the spleen specifically (Figure S3 A, B). This is not surprising, as AZA has been shown to significantly affect HSC and leukemic stem cells (LSC) in the spleen, causing their differentiation and death (Kornblau et al. 1999;, Kovacsovics et al. 2018). Analysis of immune subsets in blood following AZA treatment, however, appears to decrease the frequency of suppressive cell type scores (neutrophils and Treg), while increasing anti-tumor cell type scores like CD8 + T-cells, which is conducive for anti-leukemic immunity.

### Gene pathway alterations in blood induced by AZA treatment are largely anti-tumorigenic

**Fig. 3:**
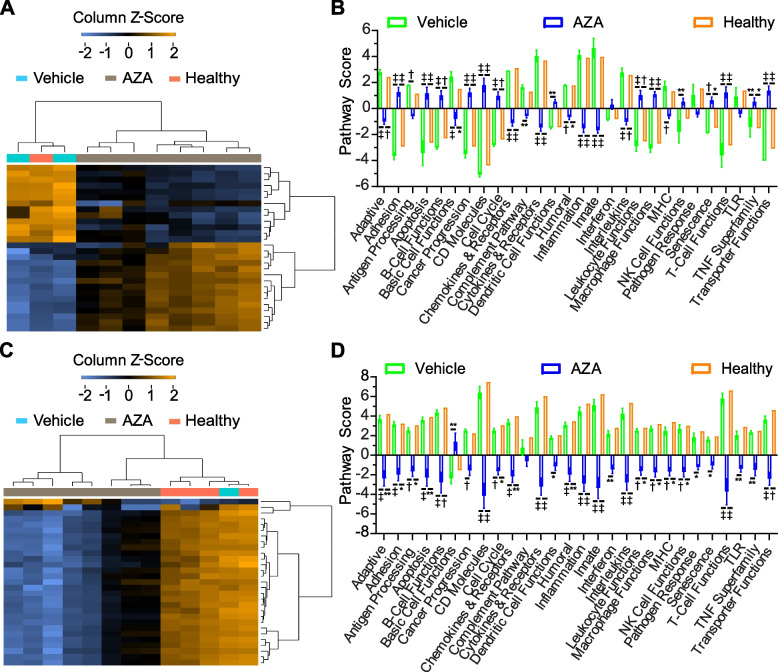
In-depth Analysis of Immune Pathways by Gene Expression. Expression levels of genes from the Mouse PanCancer Immune Profiling Panel were used to calculate scores for various immune-related pathways. Pathway scores are presented as heatmaps and bar graphs comparing non-leukemic, untreated (Healthy), DMSO-treated leukemic mice (Vehicle), and AZA-treated leukemic mice (AZA) for blood (**A**, **B**) and spleen (**C**, **D**) samples. Statistical significance was analyzed using 2-way ANOVA followed by Tukey’s test for multiple comparisons: *p < 0.05, **p < 0.01, †*p* < 0.001, ‡*p* < 0.0001

Using gene expression data from the large-scale immune panel assay, weighted scores were developed for multiple immune response pathways (Fig. 3). In blood, C1498-vehicle and Healthy samples clustered together regarding overall pathway scores (Fig. 3A). Compared to both C1498-vehicle and Healthy blood samples, C1498-AZA mice scored significantly higher in adhesion, apoptosis, B-cell functions, cancer progression, CD molecules, cell cycle, leukocyte functions, macrophage functions, senescence, T-cell functions, tumor necrosis factor superfamily, and transporter functions. In dendritic cell and NK cell functions, C1498-AZA samples scored higher than C1498-vehicle samples, but were statistically comparable to Healthy. C1498-AZA mice scored significantly lower than C1498-vehicle and Healthy in adaptive immune pathways, basic cell functions, chemokines and receptors, cytokines and receptors, humoral immune pathways, inflammation, innate immune pathways, and interleukins. In the complement pathway and MHC signaling, C1498-AZA samples scored significantly lower than C1498-vehicle but not Healthy (Fig. 3B). In spleens, almost every pathway score was significantly lower in C1498-AZA samples compared to both C1498-vehicle and Healthy samples (Fig. 3C, D), again likely due to overall reduced reads for each gene (Figure S3B).

Mirroring the decrease in pro-tumorigenic, immunosuppressive cell type scores in AZA-treated blood, decreased scores in inflammation and innate immune pathways along with increased T- and B-cell function scores appear to indicate an anti-tumor phenotype. There are conflicting data, however, such as decreased scores for adaptive immune signaling and basic cell functions, with increased scores for cancer progression and macrophage functions. These could indicate the persistence of suppressive cell types that decrease the adaptive immune response. A global decrease in pathway scores for C1498-AZA spleen samples also mirrors the downregulation of overall immune cell type scores seen in Fig. 2D. Taken together, these transcriptional profiles highlight the complex changes occurring in the non-cancerous cells of blood and spleen which may explain anti-leukemic AZA efficacy, while others may reflect compensatory mechanisms leading to resistance and relapse.

### Differential gene expression analysis identifies common targets in blood and spleen modulated by AZA

**Fig. 4:**
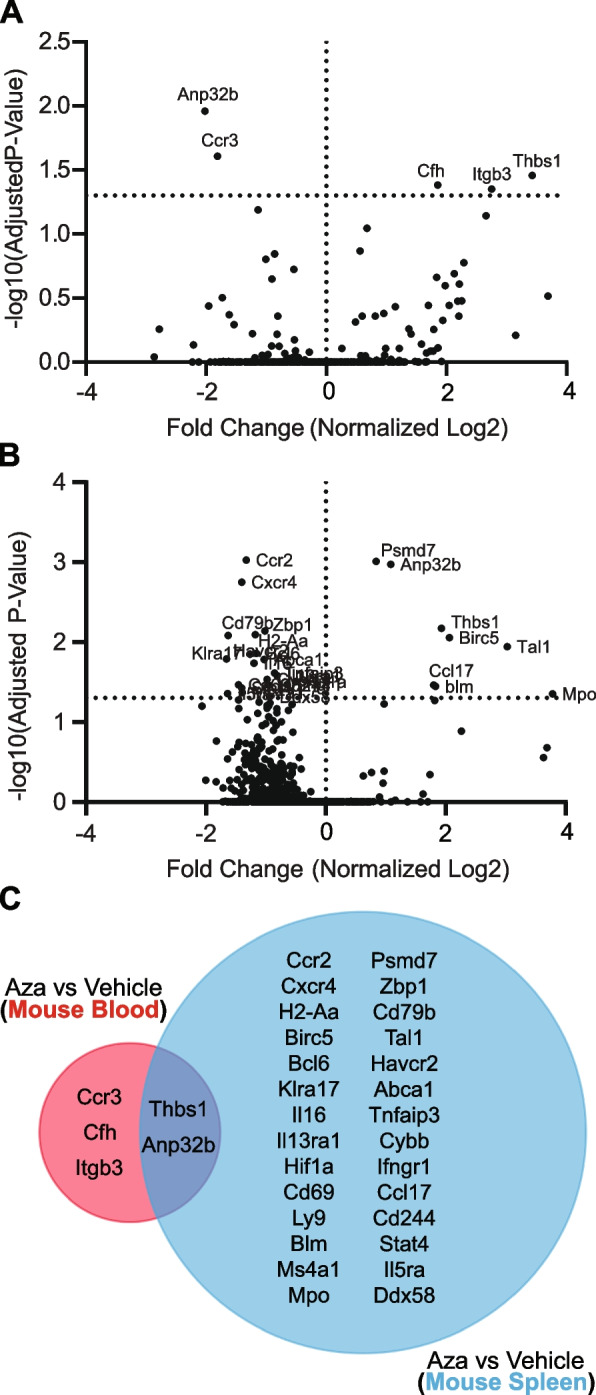
Differential Gene Expression Analysis Between Vehicle- and AZA-treated Blood and Spleen Samples from Leukemic Mice. Differentially expressed genes from the Mouse PanCancer Immune Profiling Panel between DMSO-treated leukemic mice (Vehicle) and AZA-treated leukemic mice (AZA) are shown in volcano plots as fold change (log2) versus adjusted *p*-values (-log10). Fold changes are presented as AZA versus vehicle for blood (**A**) and spleen (**B**) samples with significant genes labeled. Statistical significance was analyzed with multiple unpaired t-tests and corrected for multiple comparisons using the Holm-Sidak method. **C** A Venn diagram of genes shared between blood and spleen samples is presented

When comparing specific gene expression differences in C1498-AZA to C1498-vehicle blood samples, five distinct genes were significantly, differentially expressed: *Anp32b* (−2.02), *Ccr3* (−1.81), *Cfh* (1.85), *Itgb3* (2.75), and *Thbs1* (3.43) (Fig. 4A). In spleen samples, 30 candidate genes had a significant fold change in expression in C1498-AZA compared to C1498-vehicle: *Klra17* (−1.65), *Il5ra* (−1.64)*, Cd79b* (−1.63), *Cd69* (−1.46)*, Cxcr4* (−1.40)*, Stat4* (−1.40), *Ms4a1* (−1.40)*, Ccr2* (−1.33), *Havcr2* (−1.27), *Il16* (−1.20), *H2-Aa* (−1.17), *Bcl6* (−1.16), *Abca1* (−1.03), *Zbp1* (−1.02), *Cybb* (−0.98), *Ly9* (−0.96), *Cd244* (−0.95), *Ddx58* (−0.94), *Tnfaip3* (−0.85), *Il13ra1* (−0.81), *Ifngr1* (−0.64), *Hif1a* (−0.50), *Psmd7* (0.84), *Anp32b* (1.08), *Ccl17* (1.80), *Blm* (1.83), *Thbs1* (1.93), *Birc5* (2.06), *Tal1* (3.03), and *Mpo* (3.78) (Fig. 4B). Two targets, *Thbs1* and *Anp32b*, are shared between blood and spleen datasets (Fig. 4C). *Anp32b* is downregulated in blood and upregulated in spleen after AZA treatment, *Thbs1* is upregulated in both blood and spleen after AZA treatment. *Thbs1*is a strong inhibitor of angiogenesis (Krenn 2022), and high levels in blood serum are associated with better prognosis in AML patients (Laverdière et al. 2016).

### Gene targets modulated by AZA are primarily expressed on cell surfaces and function in adhesion, angiogenesis and platelet aggregation in mouse and human blood samples

**Fig. 5:**
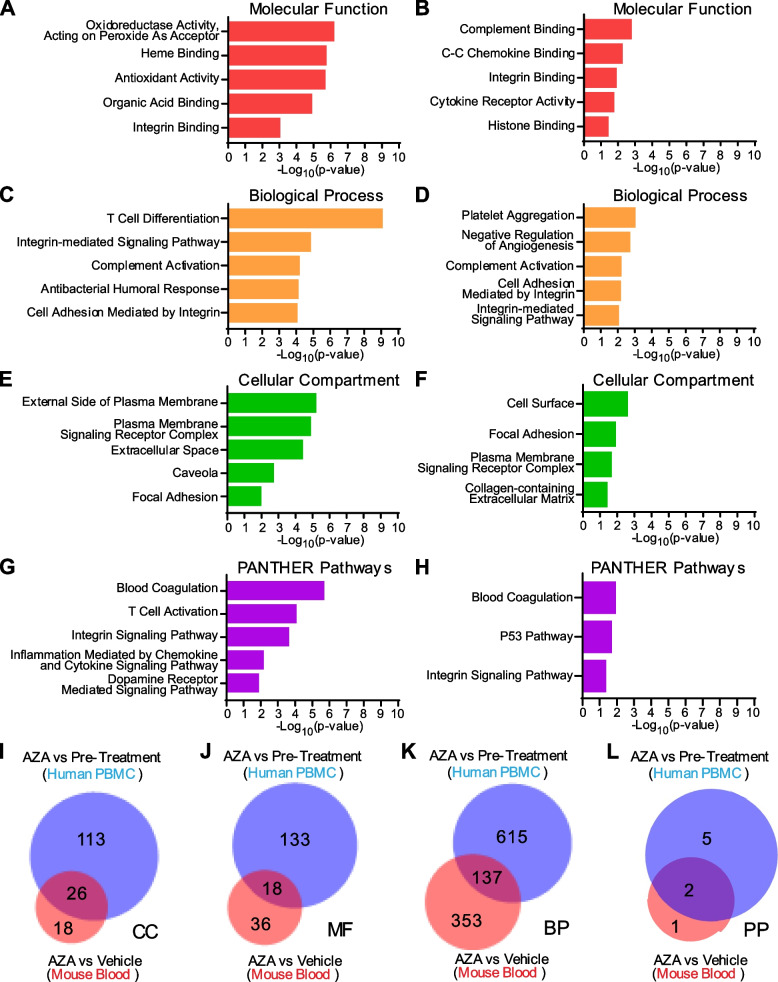
Gene Ontology (GO) Annotations in Common Between Mouse and Human Differentially Expressed Genes. Differentially expressed genes from the Mouse PanCancer Immune Profiling Panel between DMSO-treated leukemic mice (C1498-Vehicle) and AZA-treated leukemic mice (C1498-AZA) were analyzed for statistical overrepresentation analysis in gene ontology annotations. The same analysis was performed for differentially expressed genes from Gene Expression Omnibus (GEO) data set GSE118558 when comparing peripheral blood mononuclear cells (PBMC) from AZA-responsive AML and MDS patients after three complete cycles of AZA treatment versus each patient’s matched, pre-treatment PBMC. The top five annotations significantly overrepresented from the PANTHER GO “Slim” analysis are represented in bar graphs plotted against the negative log (base 10) of their *p*-values. Annotations from the subontologies “Molecular Function”, “Biological Process”, “Cellular Compartment”, and “PANTHER Pathways” are presented for human (**A**, **C**, **E**, **G**) and mouse (**B**, **D**, **F**, **H**) differentially expressed genes, respectively. A separate analysis of the same differentially expressed genes was run for the complete list of statistically significant enriched GO terms. Terms in common between mouse (red) and human (blue) are represented in Venn diagrams for (**I**) Cellular Component (CC), (**J**) Molecular Function (MF), (**K**) Biological Process (BP), and (**L**) PANTHER pathways (PP)

Despite most available human datasets containing only leukemia-burdened bone marrow or sorted leukemic blasts, we did find one dataset of peripheral blood mononuclear cells (PBMC) harvested from AZA-responsive, AML patients. Evaluating RNAseq expression data from these patients after three complete cycles of AZA treatment compared to pre-treatment, we identified a list of genes that were significantly differentially expressed (Table S2). Out of the 340 genes found to be significantly affected by AZA treatment, only *ITGB3* was a common gene upregulated in our AZA-targeted gene candidates in blood and the patient gene set; however, when genes from the mouse and human blood datasets were analyzed for functional enrichment, considerable overlap was observed in the subontologies of Cellular Compartment, Molecular Function, Biological Process and PANTHER pathways (Fig. 5). Using the PANTHER gene ontology (GO) “Slim” analysis to return the top five most significant, non-redundant annotations, we found that the human differentially expressed genes were in enriched in the biological processes and pathways of T-cell activation and differentiation, integrin-mediated signaling and adhesion, inflammation mediated by chemokines, cytokines, and complement, as well as blood coagulation (Fig. 5A, C, G). Differentially expressed genes from our mouse dataset were in enriched in blood coagulation and platelet aggregation, integrin signaling and adhesion, angiogenesis, complement activation, and p53 pathway signaling (Fig. 5B, D, H). Gene products from both datasets were enriched in expression at the plasma membrane, focal adhesions, and in the extracellular space (Fig. 5E, F).

A statistical overrepresentation analysis was also run for the complete list of gene ontology annotations in which both the mouse and human differentially expressed genes were found to be significantly enriched (Full annotations lists Table S3 (Mouse) and Table S4 (Human)). Annotations in common between human and mouse blood samples revealed that their products are expressed in the extracellular space, cell surface, integrin and adhesion complexes, and in platelet alpha granules (from the gene subontology “Cellular Component”) (Fig. 5I). These products function in immune receptor activity, protein binding, growth factor binding, fibrinogen binding, chemokine binding, integrin binding, and extracellular matrix binding (from the gene subontology “Molecular Function”) (Fig. 5J), and are implicated in the control of epithelial cell proliferation, inflammatory response, leukocyte migration, lymphocyte migration, body fluid levels, cell adhesion mediated by integrin, apoptotic process, extracellular matrix organization and adhesion, fibroblast migration, myeloid cell homeostasis, platelet aggregation, platelet activation, hemostasis, wound healing, bone remodeling, smooth muscle cell migration, phosphatidylinositol 3-kinase/protein kinase B signal transduction, angiogenesis and vasculature development(form the gene subontology “Biological Processes”) (Fig. 5K). The specific pathways in common that these genes are enriched in include blood coagulation and integrin signaling (Fig. 5L).

### AZA alters leukemic disease outcome following relapse

**Fig. 6:**
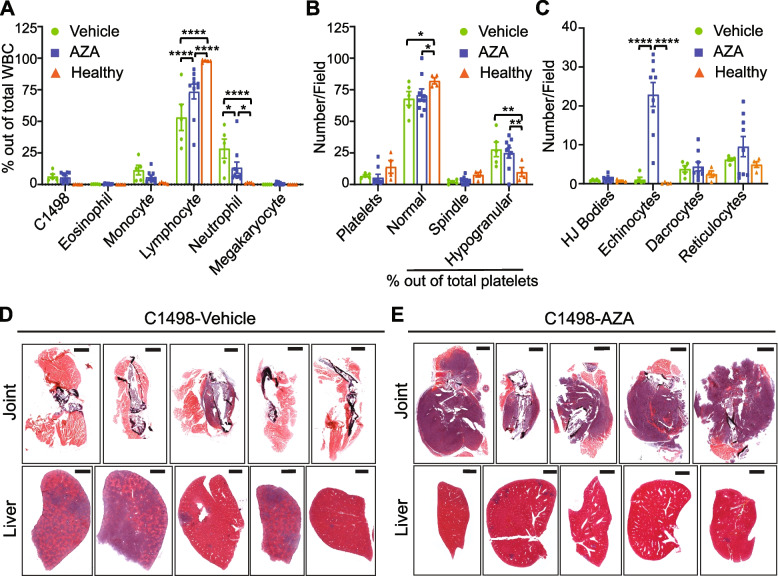
Hematologic Profiles and Tissues from Terminally Leukemic Vehicle- and AZA-treated mice. C1498 cells (80,000) were implanted in C57Bl/6 mice via tail vein injection. Treatment with DMSO in diluent (Vehicle) (*n* = 5) or 5 mg/kg/mouse AZA (*n* = 11) by intraperitoneal injection began on day 3 after implantation and continued thrice weekly until survival endpoints. Mice were euthanized once they showed signs of terminal disease including, but not limited to, labored breathing, a hunched posture, or decreased mobility. Whole blood was taken just prior to euthanasia, smeared onto slides and stained for complete blood counts. **A** White blood cells (WBC) were counted manually, including C1498, and are presented as a percentage of total WBC counted. **B** Numbers of platelets and percentages of normal, spindle-shaped, and hypogranular platelet morphologies out of total platelets are presented. **C** Red blood cell morphologies including the presence of Howell-Jolly bodies (HJ Bodies), echinocytes, dacrocytes, and reticulocytes are presented as numbers counted per field. Statistical significance was analyzed using 2-way ANOVA followed by Tukey’s test for multiple comparisons: *p < 0.05, **p < 0.01, ***p < 0.001, ****p < 0.0001. Multiple organs and tissues including hip and knee joints and livers were removed post-euthanasia, fixed, and stained by Hematoxylin and Eosin. Representative joint and liver tissues for (**D**) C1498-Vehicle and (**E**) C1498-AZA are shown (*n* = 5)

Many of these processes and pathways found to be replicated in both the mouse model and human data have implications in normal immune cell actions including cancer cell surveillance (Lawler and Lawler 2012; –Cancer 2013); however, they also have implications in leukemic proliferation, spreading to other organs via the blood stream, and even extramedullary metastatic growth of AML blasts (Li and Wu 2024; –Malik and Cashen 2014). Thus, we interrogated our model for differences in end-stage disease outcomes including the spread of C1498 growth in both vehicle and AZA-treated immunocompetent mice. The number of circulating C1498 cells at end stage disease did not differ between C1498-vehicle and C1498-AZA mice and make up about 6.63% of total WBCs (Fig. 6A). However, even at end stage disease, AZA treatment limited the expansion of neutrophils while maintaining a higher percentage of lymphocytes compared to C1498-vehicle, although not to the level of healthy mice. Total platelet counts did not differ between C1498-vehicle, -AZA, or healthy mice, but platelet morphology did differ significantly with both C1498-vehicle and C1498-AZA mice showing significantly higher percentages of hypogranular platelets with concurrent lower percentages of normal platelets compared to healthy (Fig. 6B). RBC in C1498-AZA mice showed a significant increase in echinocyte morphology compared to both C1498-vehicle and healthy blood smears (Fig. 6C).

Terminally ill C1498-vehicle mice displayed a hunched position, labored breathing, and lower body temperature prior to euthanasia. Liver enlargement and splenomegaly was observed in all C1498-vehicle mice during gross anatomy. Histochemical staining of organs revealed infiltration by cancerous cells with atypical nuclei, dense chromatin, and a high nuclear/cytoplasmic ratio most notably in the liver (Fig. 6D). Similar infiltrations were observed in C1498-AZA mice but to a lesser extent (Fig. 6E). Terminally ill C1498-AZA mice did not outwardly show signs of morbidity but were euthanized due to mobility issues. Masses were palpable on hip and knee joints (typically a single mass per mouse), sometimes causing paralysis of the affected limb. Histologically, these masses appear to be cancerous cells that have expanded within the region of the bone *rather than* spreading to distal tissues via the blood stream. Nearly 100% of relapsed C1498-AZA mice displayed more growth of cancerous cells around bone compared to liver, with the opposite being true of C1498-vehicle mice (Fig. 6D, E).

## Discussion

Gene alterations driving AML leukemogenesis and progression frequently dysregulate histone-modifiers and other epigenetic regulatory proteins (including at the level of promoter enhancers) (Mestas and Hughes 2004; –Ogana et al. 2022), this leads to unique variations on gene expression across AML clones within and between patients (Ogana et al. 2024;, Ohtani et al. 2020). Hence, the prognostic value of specific or global gene methylation or expression patterns in leukemic blasts after HMA treatment has been limited (Fraczkowska et al. 2018;, Palmieri 2020). Due to the immune modulating effects that have been reported post-AZA treatment in other cancers, we and other investigators have sought to elucidate the effects of AZA on non-cancerous immune cells in the context of leukemia in order to deduce any prognostic or mechanistic insights that might be concealed therein (Cheng et al. 2018;, Duy et al. 2019;, Haouas 2014;, Kornblau et al. 1999;, Perzolli et al. 2024;, Romer-Seibert and Meyer 2021).

So far, studies of the effects of HMAs on normal HSC in the bone marrow have highlighted the importance of non-leukemic immune and stromal cells in controlling leukemic growth after AZA treatment. In a study to determine the value of using AZA to deplete HSC prior to donor HSC transplant, they found that treatment of healthy C57Bl/6 mice with AZA led to a dose-dependent depletion of mature immune cells in peripheral blood, followed by death of HSC in the bone marrow and spleen (Kornblau et al. 1999). However, in an AML mouse model, AZA treatment caused an expansion of non-malignant HSC which favored normal immune cell production over leukemic cell production from the bone marrow (Perzolli et al. 2024). Using human HSC from healthy or leukemic donors, AZA treatment ex vivo supports the expansion of healthy HSC over LSC through an effect on co-cultured mesenchymal stem cells (Romer-Seibert and Meyer 2021). These results confirm the importance of continued study of the effects of AZA on non-malignant immune and stromal cells in order to completely understand the mechanisms of AZA-mediated leukemic control.

In this study we have utilized a transcriptomic approach to define the effects of AZA treatment of non-cancerous immune cells in the peripheral blood and spleen in leukemic mice compared to treatment with vehicle. Significantly differentially expressed genes in these compartments were analyzed for pathway enrichment. Analysis of a small, publicly available number of RNAseq datasets from normal human PBMC also revealed differentially expressed genes when comparing pre-treatment and post-treatment in AZA-responsive AML patients. Notably, although specifying a largely different set of genes from our mouse dataset, functional enrichment analysis of both the human and mouse differentially expressed genes revealed commonalities in regulation of leukocyte migration and associated signaling, integrin-based adhesion, extracellular matrix adhesion, platelet aggregation, angiogenesis, and apoptosis. Since these transcripts were detected in mice or AML patients in remission, one or more of these processes might represent a mechanism of AZA-mediated AML control by normal immune cells. Indeed, therapies targeting angiogenesis (Roulois et al. 2015), leukocytes (Sauer et al. 2004), platelet aggregation (Schmutz et al. 2023), and integrins (Shatilova et al. 2022) have been explored in treating AML, and targeting of specific adhesion molecules have been shown to synergize with HMA treatment (Shin et al. 2005).

When leukemic cells become refractory to AZA treatment in the C1498 mouse model, the resultant disease manifestation differs from vehicle-treated C1498 such that growth in the liver is overall decreased in C1498-AZA compared to C1498-vehicle mice, while growth in and around the long bones of the mice is more prevalent. If AZA treatment of normal immune cells is somehow modulating angiogenesis, adhesion, and platelet aggregation in a way that limits C1498 spread, then this might account for the altered C1498 manifestation we see after relapse. Interestingly, AZA + venetoclax treatment has shown improved success over standard chemotherapy specifically in the setting of extramedullary AML growth (Silverman et al. 2011; –Su et al. 2016). Determining the frequency of extramedullary disease when using chemotherapy, AZA alone, or AZA + venetoclax in the C1498 AML model could reveal additional benefits of using BCL-2 inhibitors in combination with HMAs.

Within this study we acknowledge there are several limitations that restrict the strength of our conclusions. First, the human transcriptomic data come from blood samples filtered for PBMC, while our mouse blood samples contain whole blood. The lack of some immune cell types (granulocytes, etc.) and other components like platelets in the human PBMC samples would certainly produce different results than if whole blood was used. However, mouse whole blood and human whole blood have very different compositions in that WBC are comprised of mostly neutrophils in humans but are largely lymphocytes in mice (Tobiasson et al. 2017). It could thus be argued that PBMC is more comparable to mouse whole blood than human whole blood, but loss of platelets and other factors in the PBMC samples remains an issue. Due to the small number of human samples analyzed in addition to the differences in blood composition and sample preparation between human and mouse data, these cross-species comparisons should be interpreted with caution. Other limitations include that the relationship between observed gene expression changes must be further studied to determine whether they functionally inhibit or promote any of the identified processes and pathways. Moreover, studies will be needed to functionally characterize individual immune subsets, platelets, and blasts, and their association with endothelial cells, angiogenesis, and extramedullary growth after AZA treatment.

Targeting these processes and associated genes is often challenging with respect to the paradoxical nature of their pro-tumorigenic action in leukemic cells and their anti-tumorigenic action in other cells. Regarding this study’s findings, human AML blasts express *Cfh*, *Ccr3*, *Thbs1*, *Itgb3, Anp32b* and *Stat4*, with higher expression of all being separately associated with poor prognosis (Tsao et al. 2012; –Wang et al. 2021), and expression of*Ccr3*in AML blasts is thought to drive chemokine induced proliferation (Wenk et al. 2018). Conversely, the expression of both*ITGB3* and *STAT4*gene products from non-malignant cell types in the tumor microenvironment has previously been shown to support anti-tumor immunity (Wolff et al. 2017; –Wu et al. 2022), and high THBS1 in blood serum of AML patients predicts better prognosis (Laverdière et al. 2016). Targeting integrins for AML treatment is actively being explored pre-clinically, but whether these treatments diminish the anti-tumorigenic action of circulating immune cells has not been investigated (Xiao et al. 2021;, Yi et al. 2016). Indeed, integrins are critical in normal HSC maintenance, quiescence, proliferation and differentiation (Zhang et al. 2022;, Zhu et al. 2019). Considering these caveats, great care should be taken to fully understand the interplay of adhesion, thrombosis, angiogenesis and their associated genes between leukemic cells, normal immune cells, and other stromal cells before rational combination therapies with AZA can be safely explored in the clinic. We feel that the C1498 model will be a valuable tool to further elucidate the complex mechanisms of AZA-mediated control of AML and to identify more synergistic combinations.

## Conclusion

Genes and pathways connected to angiogenesis, adhesion, and platelet aggregation have been explored in the context of leukemic blasts, but these studies provide data that normal immune subsets utilize these same mechanisms for leukemic control. Expanding the exploration of biomarkers and therapeutic targets in non-bone marrow microenvironments and the associated non-leukemic cells, particularly in the setting of remission, should be included in future studies of the mechanisms of HMA efficacy.

## Supplementary Information

Supplementary Material 1

## Data Availability

Mouse bulk RNA raw data files are uploaded to Gene Expression Omnibus (GEO) under series GSE283410 (access token: sncdqmukzfinhkh).
