# Supplementary material for: Transcriptomic analysis of non-leukemic cell subsets in azacytidine-responsive AML highlights pathways associated with adhesion, platelet aggregation, and angiogenesis in mice and humans
Source: Mol Med. 2025 May 13;31:185. doi: 10.1186/s10020-025-01233-2 (PMC12070539; doi:10.1186/s10020-025-01233-2)
Supplement: Supplementary file 1 — Supplementary Material 1 [file 10020_2025_1233_MOESM1_ESM.pdf]

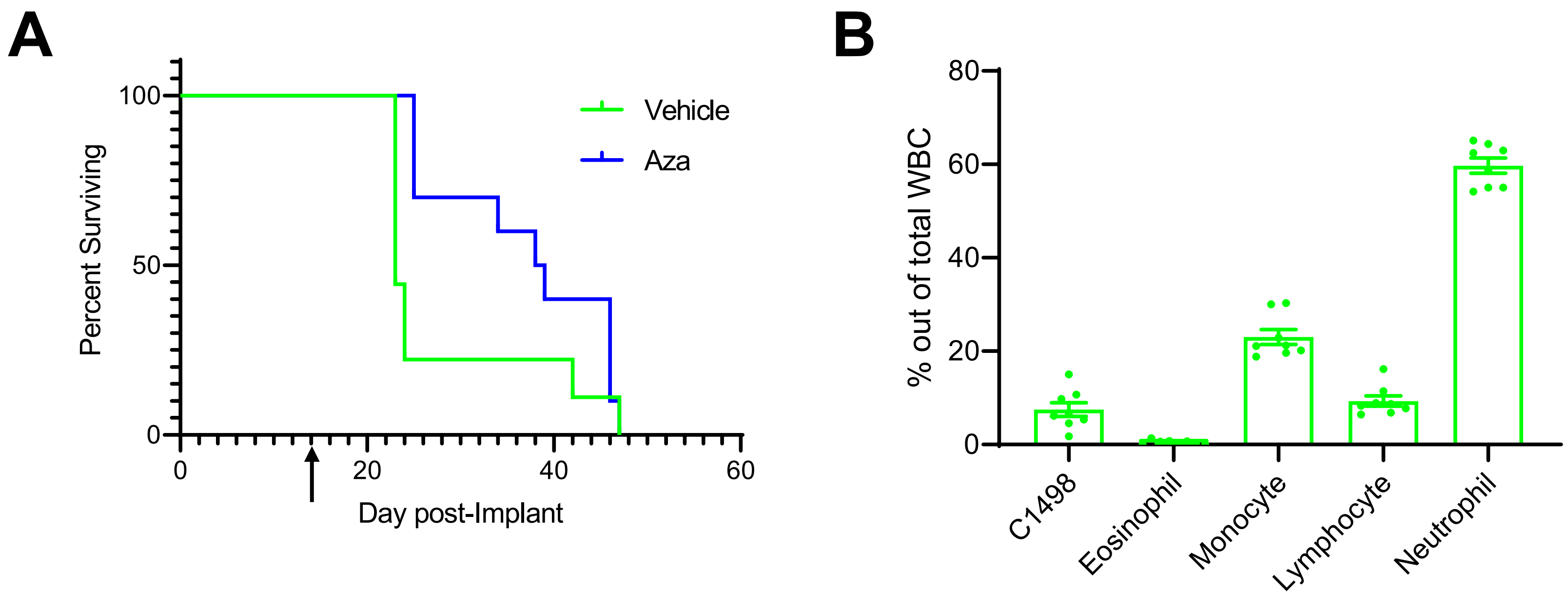

Supplemental Figure 1. Survival and Hematologic Profiles of Leukemic Vehicle- and AZA-treated Immunodeficient Mice. C1498 leukemia cells (80,000) were implanted in NSG immunodeficient mice via tail vein injection. Treatment with DMSO in diluent (Vehicle) (n=9) or 5mg/kg/mouse AZA (n=10) by intraperitoneal injection began on day 3 after implantation and continued thrice weekly until survival endpoints. **(A)** Mice were euthanized once they showed signs of terminal disease state and survival was recorded for each mouse and displayed as a Kaplan-Meier curve. Statistical significance was analyzed using the Log-rank (Mantel-Cox) test. The arrow (↑) indicates the timepoint at which retroorbital blood was drawn to assess hematologic profiles. **(B)** Drawn blood was stained and smeared for complete blood counts. Manually counted WBCs for C1498-Vehicle mice are shown as percentages of total WBC counted. Blood from C1498-AZA mice showed an extremely low WBC count that could not accurately be displayed graphically.

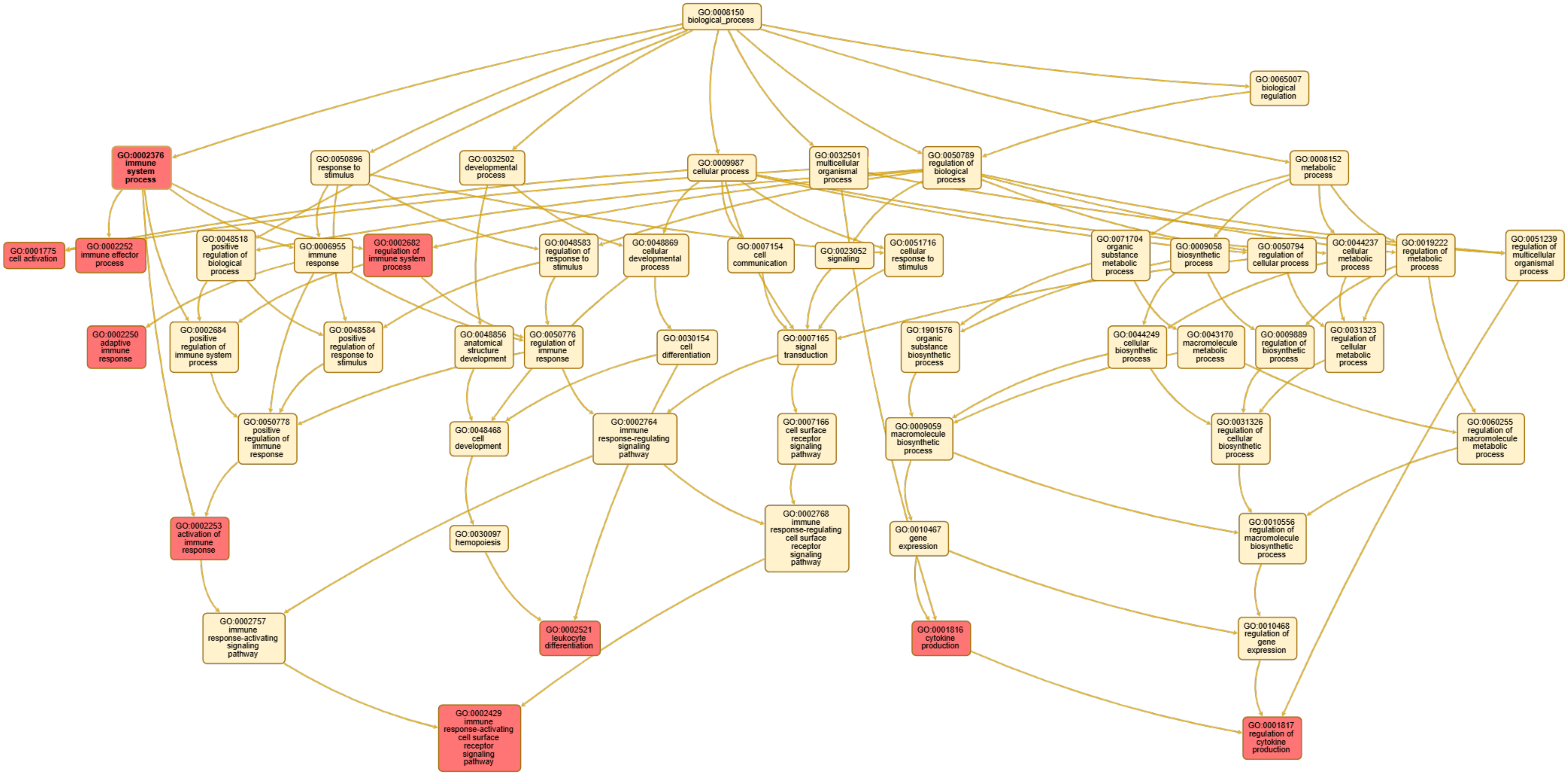

Enriched Gene Ontology Categories

GO Identification	GO Name
GO:0001775	Cell Activation
GO:0001816	Cytokine Production
GO:0001817	Regulation of Cytokine Production
GO:0002250	Adaptive Immune Response
GO: 0002252	Immune Effector Process

GO Identification	GO Name
GO:0002253	Activation if Immune Response
GO:0002376	Immune System Process
GO:0002429	Immune Response-Activating Cell Surface Receptor Signaling Pathway
GO:0002521	Leukocyte Differentiation
GO:0002682	Regulation of Immune System Process

Supplemental Figure 2. Map and Listing of Gene Ontologies Enriched in the Mouse PanCancer Immune Profiling Panel. Genes to be probed for expression were analyzed for Network Topology using the WEB-based Gene Set Analysis Toolkit (WebGestalt). The mapping of enriched gene ontologies is shown in red with the linked ancestor ontologies in off-white. The top ten enriched ontologies are shown with their Gene Ontology (GO) identification number.

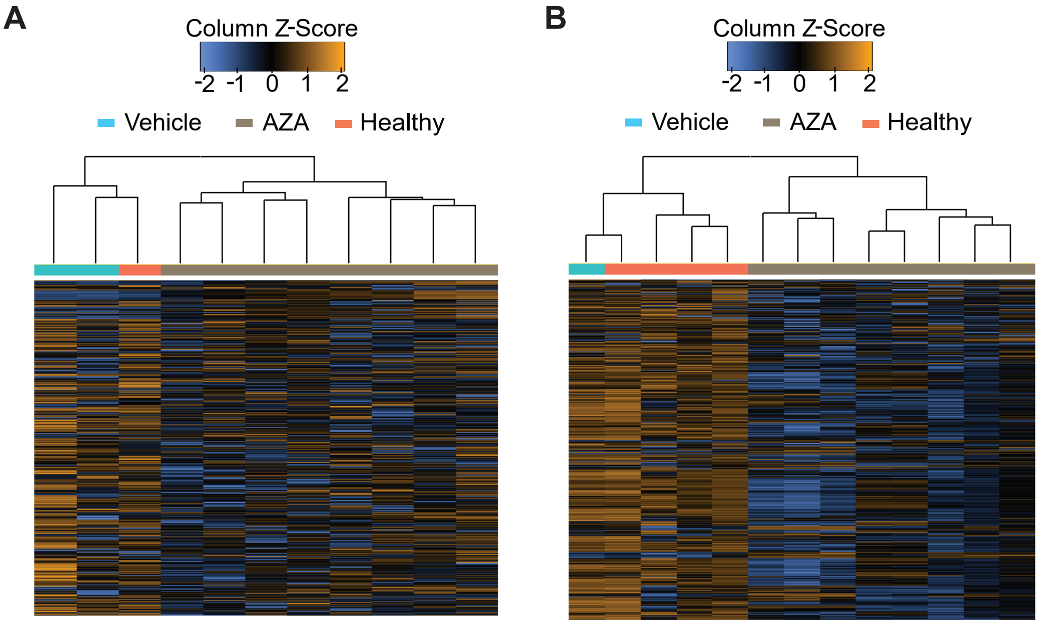

Supplemental Figure 3. Heatmap of total reads for all genes by sample using the Mouse PanCancer Immune Profiling Panel. Total reads for each gene by sample from **(A)** whole blood or **(B)** whole spleen of C1498-vehicle, C1498-AZA, and Healthy mice.

Supplemental Table 1. "Panel Plus" user added genes to the Mouse PanCancer Immune Profiling panel with optimized probe target sequence followed by the complete gene list from the Mouse PanCancer Immune Profiling Panel.

"Panel Plus" Genes

Customer Identifier	Accession	Position	Target Sequence
**abat**	NM_001170978.1	3561-3660	GAGCCACAGTGTCATATACAGATACTTCCGCAGGTCCTTAGAGTTCAAAGGGTTTTAATCCAGGACATAAGCAGAAATCGCTCTCTTTAGTGAAGGGAGC
**Akt1**	NM_001165894.1	899-998	GCCATGAAGATCCTCAAGAAGGAGGTCATCGTCGCCAAGGATGAGGTTGCCCACACGCTTACTGAGAACCGTGTCCTGCAGAACTCTAGGCATCCCTTCC
**Bad**	NM_007522.3	1147-1246	TTCGAGGCCTTAGGAAAAAAAAAGAGGATCGCTGTGTCCCTTTAACAGGGAGAAGAGCTGACGTACAGCTTGAGTCCCTTCCGGTGCGTGCAATAGCCAC
**bbc3**	NM_133234.1	1462-1561	CCCCAATCCCCATCCATCTCATTGCATAGGTTTAGAGAGAGCACGTGTGACCACTGGCATTCATTTGGGGGGTGGGAGATATTGGCGGAAGCCACCCCAG
**birc3**	NM_007464.3	426-525	CCCTGTCATCTCACCATGAACATGGTTCAAGACAGCGCCTTTCTAGCCAAGCTGATGAAGAGTGCTGACACCTTTGAGTTGAAGTATGACTTTTCCTGTG
**blm**	NM_001042527.2	265-364	AAAGATGTGAACGTGTCTGAGGCCTTTTCATTCACTGAGTCTCCACTCCACAAACCAAAGCAGCAGGCAAAGATTGAAGGCTTCTTTAAACATTTCCCTG
**casp9**	NM_015733.4	1676-1775	ATAACTGTCCTGCTAAGATAGGATTTTGAACTGGGGCAGGCTGCTCTTTCCCTTTGGCGATGCAAACATGCTCCTAGCAGCTTTCAGGTTGTAGGGCAAT
**ctnnb1**	NM_007614.2	2976-3075	TTGGTCGAGGAGTAACAATACAAATGGATTTGGGGAGTGACTCACGCAGTGAAGAATGCACACGAATGGATCACAAGATGGCGTTATCAAACCCTAGCCT
**dbn1**	NM_019813.4	2571-2670	CGAAATTTAAAACATGGCAATAAATGGCTCGTGGGCTCTGGCTCCCTGGGACCCTTCCCCTTCTCTTTACCCTCGCTGCTTGGTCAGAAGGAATTATCAG
**ddx52**	NM_030096.2	501-600	AGGAAAAGGTCAACTTCTTTCGGAACAAGCACAAGATACATGTCCAAGGAACTGATCTTCCTGACCCAATTGCTACATTTCAGCAACTTGACCAGGAATA
**mdm2**	NM_010786.4	1665-1764	GTCATGTTTCACGTGTGCAAAGAAGCTAAAAAAAAGAAACAAGCCCTGCCCAGTGTGCAGACAGCCAATCCAAATGATTGTGCTAACTTACTTCAACTAG
**pdk4**	NM_013743.2	1356-1455	AGCGGATGACGCCTGACATTTTACGGGATCAAAGTGGGTCTGTGGCATTGCTGCTTCGTGAATGTGTGTGGACTCTAGTTTCCGCAAAACAACGCAACAC
**psmb5**	NM_011186.1	335-434	TGCAGCTTCTGGGAGCGGTTGTTGGCTCGGCAGTGTCGAATCTATGAGCTTCGCAATAAGGAACGCATCTCGGTCGCAGCAGCCTCCAAACTGCTCGCTA
**psmc4**	NM_011874.2	1147-1246	TCGACCTGGAAGACTATGTGGCCCGTCCAGATAAGATTTCAGGAGCCGATATCAACTCCATCTGTCAGGAGAGTGGAATGTTGGCTGTCCGTGAGAACCG
**Ripk1**	NM_009068.3	3186-3285	GCTTTGGCCTTGTTGGCCATTCTGGCACTCATTGGCACTTCATCCTCCTTTGTTGGGCTATCCTGTACTCAGTAGGATATTTGGGAACATTCCTGGCCTC
**Ripk3**	NM_019955.1	271-370	CACAGAACATGGAACCATGATGTAGCAGTCAAGATCGTGAACTCGAAGAAGATATCCTGGGAGGTGAAGGCTATGGTTAATCTTCGTAATGAGAACGTTC
**rock1**	NM_009071.2	416-515	AAGTTGGTTGAACTTGCTTTCCGCTGCGGGCAAGAAGGTATCGTCACAAGTAGCAGCATCATGTCGACTGGGGACAGTTTTGAGACTCGGTTTGAAAAAA
**slc24a3**	NM_053195.2	2271-2370	CAGGAGAGGGTCCGCTGATGGCAGGAAGGTTTGTTTTGTTTGGGAGTGAGTCCTAGGTTACGGGGCTCAGGGAAATTGTTTAATTTGAGGGGGCGCTTTT
**tnfsf9**	NM_009404.3	293-392	GCTCTATGGCCTAGTCGCTTTGGTTTTGCTGCTTCTGATCGCCGCCTGTGTTCCTATCTTCACCCGCACCGAGCCTCGGCCAGCGCTCACAATCACCACC
**ubb**	NM_011664.4	1324-1423	TCCTCCGTCTGAGGGGTGGCTATTAATTATTCGGTCTGCATTCCCAGTGGGCAGTGATGGCATTACTCTGCACTCTAGCCACTTGCCCCAATTTAAGTTT

Mouse PanCancer Immune Profiling Panel

Official Symbol	Accession	Target Sequence
**A2m**	NM_175628.3	AGAGGACATGTATGGCTTCCTAAAGGATATGGGCTTAAAGGTATTCACCAACTTGAACATCCGTAAACCCAAAGTCTGTGAACGCCTTGGAGTCAATAAA
**Abca1**	NM_013454.3	CTCCTTGTCATCTCTAGCCAGGATATTCAGCATCCTCTCCCAGAGCAAAAAGCGACTCCACATAGAAGACTACTCTGTCTCTCAGACAACACTTGACCAA

**Abcb1a**	NM_011076.1	AAGGGGCTACAGGGTCTAGGCTTGCTGTGATTTTCCAGAACATAGCAAATCTTGGGACAGGAATCATCATATCCCTAATCTATGGCTGGCAACTAACACT
**Abcg1**	NM_009593.1	GCGGCCGTGAACATCGAATTCAAGGACCTTTCCTACTCTGTACCCGAGGGGCCCTGGTGGAAGAAGAAAGGATACAAGACCCTTTTGAAAGGGATCTCTG
**Abl1**	NM_009594.4	ATTGCTCTGGGAGATTGCTACCTATGGCATGTCACCTTACCCGGGAATTGACCTGTCTCAGGTTTATGAGCTGCTGGAAAAAGACTACCGCATGGAGCGC
**Ada**	NM_007398.3	GACATGGTTATCACACCATCGAGGATGAAGCTCTCTACAACAGACTACTGAAAGAAAACATGCACTTTGAGGTCTGCCCCTGGTCCAGCTACCTCACAGG
**Adora2a**	NM_009630.2	ACTATGAAAGGCAGGGTGCCAGAGTGTGGGCTCACGTCTCAGGATTGAGTTTAGAGACATCAAGTCATGGACCCGAGCTGGATAGTTCAGAGCTGCCCTG
**Aicda**	NM_009645.2	AATCGTGAAAGAACTTTCAAAGCCTGGGAAGGGCTACATGAAAATTCTGTCCGGCTAACCAGACAACTTCGGCGCATCCTTTTGCCCTTGTACGAAGTCG
**Aire**	NM_009641.1	TCCAGGAACCCGAGGGGCCGTGGAAGGGATCCTTATCCAGCAGGTGTTTGAGTCAGGAAGATCCAAGAAGTGCATTCAGGTTGGGGGAGAGTTTTATACA
**Akt3**	NM_011785.3	GTTGTCATGTTAGCAAGCGCAGCTCCAATTCACAGATCATTGCCTGCGTTTTCTTTTGACCCATGTGCAAGGAATGTACACACCCATTAGAATCATGCAC
**Alcam**	NM_009655.1	ATTTATCCTTAAACCCAAGTGGGGAGGTGACCAAGCAGATTGGCGATACCCTGCCTGTGTCATGCACAATATCTGCAAGTAGGAACGCGACTGTGGTGTG
**Ambp**	NM_007443.3	CCACAAATCCAAATGGAACATAACCTTGGAATCCTATGTGGTCCACACCAACTATGACGAATATGCCATTTTCCTTACCAAGAAGTCCAGCCACCACCAC
**Amica1**	NM_001005421.4	TTTCAAAGGACAATTTCACCAACTCCTCCAACTGATAAGGGTCAGCAGGGCATCCTGAATGGAAATCAGCTGGTGATCATTGTGGGGATCGTCTGTGCCA
**Angpt1**	NM_009640.3	GGGCTGGAAGGAGTATAAAATGGGTTTTGGGAATCCCTCTGGTGAATATTGGCTTGGGAACGAGTTCATTTTTGCAATAACCAGTCAGAGGCAGTACATG
**Angpt2**	NM_007426.3	GAAGAATGTTCCGTGGGAGTTCAGCAGTAAATAACTGGAAAACAGAACACTTAGATGGTGCAGATAAATCTTGGGACCACATTCCTCTAAGCACGGTTTC
**Anp32b**	NM_130889.2	AAGATATCAGCACCTTGGAGCCGTTGAAAAGGTTGGATTGTCTGAAAAGCCTGGATCTGTTTGGCTGTGAGGTCACTAACCGGAGTGATTACCGAGAAAC
**Anxa1**	NM_010730.2	GGCTATGCTAAAAACTCCAGCTCAGTTTGATGCAGATGAACTCCGTGGTGCCATGAAGGGACTTGGAACAGATGAAGACACTCTCATTGAGATTTTGACA
**Apoe**	NM_009696.3	GCTAAGGACTTGTTTCGGAAGGAGCTGACTGGCCAATCACAATTGCGAAGATGAAGGCTCTGTGGGCCGTGCTGTTGGTCACATTGCTGACAGGATGCCT
**App**	NM_007471.2	GTGAGCGACGCCCTTCTCGTGCCCGACAAGTGCAAGTTCCTACACCAGGAGCGGATGGATGTTTGTGAGACCCATCTTCACTGGCACACCGTCGCCAAAG
**Arg1**	NM_007482.3	GTACATTGGCTTGCGAGACGTAGACCCTGGGGAACACTATATAATAAAAACTCTGGGAATTAAGTATTTCTCCATGACTGAAGTAGACAAGCTGGGGATT
**Arg2**	NM_009705.2	GGCTCTCCAGGTTGGGATGCCACCTAAAAGACTTTGGAGACTTGAGTTTTACTAATGTCCCACAAGATGATCCCTACAATAATCTGGTTGTGTATCCTCG
**Atf1**	NM_007497.3	GAAGCTCCGAAAAGATAGAGTGCTCAAGGAATGGACTTTCAGCAGTTTAAATAGAAATAATGGTCAGCAACGAAAATGGCAGGCGACGTTAGTGAAGCAG
**Atf2**	NM_001284371.1	AGAGCAGCAGCTTCAAGATGCCGACAAAAAAGGAAAGTGTGGGTTCAGTCCTTAGAGAAGAAAGCAGAAGACTTGAGTTCACTAAATGGCCAGCTGCAGG
**Atg10**	NM_025770.3	GGGTTCTCATTAACATGGAGGATGAGTTCTTTGGAGAAAAAAGCTTCCAGCATTATTGTGCAGAATTCATCAGACATTCACAGCAGATAGGCGATGGCTG
**Atg12**	NM_026217.1	CATGGCAGCACTCCTAGCATTGAGGAAGTGGAAACAAGCAGACTGAAAGTTTAAGACTGTGTGGGCTACAGAGTGAGATTGTCTCACCTCTGGGGGAAAA
**Atg16l1**	NM_001205391.1	CGTTCGAGGAGATCATTCTGCAGTATACCAAGTTGCTGGAAAAGTCAGATCTTCATTCAGTATTGACCCAGAAACTACAAGCAGAAAAGCATGACATGCC
**Atg5**	NM_053069.5	CATTACCAAAAACATCCAGAGGTTACTGGAACTCCTACCACAGTAAGGAAAGTTTGCTGCACTCTCTCGATGGCTGCTTGGAGACTCCTGCTGTTGATTT
**Atg7**	NM_028835.1	TGCAGGGAGCTAGAGACGTGACACATAGCATCATCTTTGAAGTGAAACTTCCAGAAATGGCATTTAGCCCAGATTGTCCTAAAGCTGTTGGCTGGGAGAA

**Atm**	NM_007499.2	TTCCTCAAAGTGAAAGTCATGACATTTGGATAAAGACACTGACGTGTGCCTTTCTGGACAGTGGAGGCATAAACAGTGAAATTCTCCAGTTATTAAAGCC
**Axl**	NM_009465.3	GAGATTCTAAAGGTCCACAGTCTAGAGTATTAGGTACGACTCCAAGGGTGGGCGCTTGTAGCCATCCTAAGTCCTTTCCCTCCTTAAGCACCTATGCTCC
**Batf**	NM_016767.2	ACTGGACTCTCCCAGGGAAGTCGAGCCAATGTACTGGACCCAAAAAATGACAAGTCAACCCTGGACTGTCATGAATGATGCCCAAAATACACAGCACAGA
**Bax**	NM_007527.3	CATAAATTATGACATTTTCCTGGGATGAATGGGGGAAGGGGAAAGGCATTTTTCTTACTTTTGTAATTATTGGGAGGGGTGGGAATGGTGGCCTGGGGAG
**Bcl10**	NM_009740.1	TTTAGCAGTAAGCTGTTGTGTTTCAGGAAAGCTGGACACGGGAAAGCTGCCGACACACTCAGCAGTGTCCCACTCCTTAGTTCTGAGAAGCCGTCGGGTT
**Bcl2**	NM_009741.3	GGCCTTCTTTGAGTTCGGTGGGGTCATGTGTGTGGAGAGCGTCAACAGGGAGATGTCACCCCTGGTGGACAACATCGCCCTGTGGATGACTGAGTACCTG
**Bcl2l1**	NM_009743.4	GAGCAACCGGGAGCTGGTGGTCGACTTTCTCTCCTACAAGCTTTCCCAGAAAGGATACAGCTGGAGTCAGTTTAGTGATGTCGAAGAGAATAGGACTGAG
**Bcl6**	NM_009744.3	ACGTTGTCATCGTGGTGAGCCGTGAGCAGTTTAGAGCCCATAAGACAGTGCTCATGGCCTGCAGCGGCCTGTTCTACAGTATCTTCACTGACCAGTTGAA
**Bid**	NM_007544.3	AATCATGCACACGACTTATCTGTGTGGTGTCAGTTACACTCAGGCTCTTGCTACGGAATGCAAAGAACAACTCACATACCAGTGTCAAACAGAATGCACA
**Birc5**	NM_009689.2	GGCTTCATCCACTGCCCTACCGAGAACGAGCCTGATTTGGCCCAGTGTTTTTTCTGCTTTAAGGAATTGGAAGGCTGGGAACCCGATGACAACCCGATAG
**Blk**	NM_007549.2	AAGGATTGTGTTAGAACTTACCTCCAGTTGTGTTCAGACCCCGATAGTCCCGAAATCAGTCTGACCAGTTAGCGATGGACAGGATGTCAGGCTGTCATAA
**Blnk**	NM_008528.4	CTGAAGAGGCCTTGCACAGATCCAACAAGGATGGATCGTTTCTTATTCGGAAGAGCTCTGGCCATGATTCCAAGCAGCCGTACACCCTAGTTGCGTTCTT
**Bmi1**	NM_007552.4	TCTCGAGGTTTTCATGGTGTTACCTAAGACAAAAGACATCTCACCCTCTATGATGGACTTACTTCTGAGAGTGCGTTTGAGGCACTTATGGCTTACTAAG
**Bst1**	NM_009763.3	TCATGAACTCAGCCTCAGCATCTATGCGGAGGGAGAGCGCATCTCTTCATGCAATAGGAGACGCCAGCCTTCTCATTTCTCTCTTAGTAGCTTTGGCTTC
**Bst2**	NM_198095.2	CAGCTCCATGGTGGTCTCCAGCCTACTGGTGCTCAAAGTGTCACTGTTCCTGCTCTTTTGAGGACTCATTAGTTGGCAGGTCACAGTTGTTTGAAGTCAC
**Btk**	NM_013482.2	GAAGAGCCCTGGACATCTAGGAATGCCTTTCTTCTCTCGTTCCCTGGCATACTGCTCTAAGCAAAGGTCAAGGGATTTCTGTGCCTAGTATTACCCATAA
**Btla**	NM_001037719.2	AGTGCCTGCCATGCTTGGGACTCCTCGGTTATTTAGGGAATTCTTCATCCTCCATCTGGGCCTCTGGAGCATCCTTTGTGAGAAAGCTACTAAGAGGAAT
**C1qa**	NM_007572.2	CAGTGCCCGGCTTCTATTACTTCAACTTCCAAGTGATCTCCAAGTGGGACCTTTGTCTGTTTATCAAGTCTTCCTCCGGGGGCCAGCCCAGGGATTCCCT
**C1qb**	NM_009777.2	GTGCCAACAGCATCTTCACTGGCTTTCTGCTTTTCCCTGACATGGATGCGTAATCACGGGGTCAAATTACACCTATCCAACACCATCTTCCTGCCTCCCT
**C1qbp**	NM_007573.2	TCCTGAGGATGAGATTGGACACGAAGATGAGGCCGAGAGTGATATTTTCTCTATCAAGGAAGTTAGCTTTCAGGCCACTGGTGACTCTGAGTGGAGGGAT
**C1ra**	NM_023143.3	CAATGAGACCTTCTATGGCCAAGGCCTCATGGGTTATGTCAGCGGATTCGGGATAACAGAAGATAAGTTAGCTTTCGATCTCAGGTTCGTCAGACTGCCT
**C1s1**	NM_144938.2	GGCAGGGTTGATCACTGAGCCGTGTTGGTTATTCAGTTACTATTGCTAACAACATGGCAGAAACCCTTCTATCTTGCGCTATTCCACAGGGATATCTTAT
**C2**	NM_013484.2	ATGGTGTCCTGGACTTTCTGCCACTTTAACATGGTCACTGACTCCTTTATTAGTCTGAACTTCCTGTCTAATACCTCTGAGCGTTCTCACTCCTGGATAC
**C3**	NM_009778.2	AAGACTTCCTAAAGAGGCAAGTGCTGACCAGTGAGAAGACAGTGTTGACAGGAGCCAGTGGACATCTGAGAAGCGTCTCCATCAAGATTCCAGCCAGTAA
**C3ar1**	NM_009779.2	ATAAGCCAATCTGGTGCCAGAATCATCGAAACGTGAGAACCGCCTTCGCCATCTGTGGATGTGTCTGGGTGGTAGCCTTTGTGATGTGTGTGCCCGTATT
**C4b**	NM_009780.2	GTGCAGACCGATCAGCCTATCTATAATCCGGGGCAGCGGGTTCGTTATCGGGTCTTTGCACTGGATCAAAAGATGCGCCCATCCACTGATTTCCTCACCA

**C5ar1**	NM_007577.3	CATTGCTCCTCACCATTCCATCCTTCGTGTACCGGGAGGCATATAAGGACTTCTACTCAGAGCACACTGTATGTGGTATTAACTATGGTGGGGTAGCTT
**C6**	NM_016704.2	CTGATTGACAAGAGTGAAGCCTGTTTCTGTGACCACTACCCATGGACTCACTGGTCCAGCTGTTCTAAGTCCTGCAATTCTGGAACCCAGAGCAGACAGA
**C7**	XM_356827.6	AGACAAATGTGAAAATGTGGCTAACCCTTCCTGCAACATTGATAAGCCTCCTCCCAACATAGAACTTACCGGTTATGGTTACAACGTAGTTACTGGCCAG
**C8a**	NM_146148.2	TGGCATGTACGCCAAGTTCATCAATGACTATGGCACCCACTACATCACCTCTGGAACCATGGGTGGCATTTATGAATATGTTATGGTGCTTGACAAAGAG
**C8b**	NM_133882.2	GTTACTGCCTGTGCTGGAGGAAGTTTCGGTATTGGTGGTATGGTCTATAAAGTCTATGTCAAAGTGGGCGTTTCCGCAAAGAAATGCAGTGACATTATGA
**C8g**	NM_027062.1	GAGCGGCGAGTCAGGGAAGCCAACCTGACAGAAGATCAGATTCTTTTCTTTCCCAAGTATGGTTTCTGCGAGACTGCGGACCAATTACACATCCTGAACG
**C9**	NM_013485.1	TCCTTGCCTCAAACAAAGGTTTCGCTCAAGAAGCATTTTAGCCTTCGGACAGTTTAATGGGAAAAGCTGTGTTGATGTTTTGGGAGACAGACAAGGCTGT
**Camp**	NM_009921.2	AACCCGGCCGCTGATTCTTTTGACATCAGCTGTAACGAGCCTGGTGCACAGCCCTTTCGGTTCAAGAAAATTTCCCGGCTGGCTGGACTTCTCCGCAAAG
**Card11**	NM_175362.2	ATTGTTGGACATTCTTCACACCAAGGGACAAAGGGGCTATGTGGTCTTCCTGGAGAGCCTGGAGTTTTACTACCCAGAACTTTACAAACTGGTGACTGGA
**Card9**	NM_001037747.1	ACAGGCCACAAGGGCTACGTGGCTTTCCTCGAGAGCCTGGAGCTCTACTACCCTCAGTTATACCGGAAAGTCACTGGCAAGGAGCCAGCACGCGTCTTCT
**Casp1**	NM_009807.2	GACAATAAATGGATTGTTGGATGAACTTTTAGAGAAGAGAGTGCTGAATCAGGAAGAAATGGATAAAATAAAACTTGCAAACATTACTGCTATGGACAAG
**Casp3**	NM_009810.2	GAGGCTGACTTCCTGTATGCTTACTCTACAGCACCTGGTTACTATTCCTGGAGAAATTCAAAGGACGGGTCGTGGTTCATCCAGTCCCTTTGCAGCATGC
**Casp8**	NM_009812.2	TTTCATTCAGGCTTGCCAAGGAAGTAACTTCCAGAAAGGAGTGCCTGATGAGGCAGGCTTCGAGCAACAGAACCACACTTTAGAAGTGGATTCATCATCT
**Ccl1**	NM_011329.2	ATGCTTACGGTCTCCAATAGCTGCTGCTTGAACACCTTGAAGAAAGAGCTTCCCCTGAAGTTTATCCAGTGTTACAGAAAGATGGGCTCCTCCTGTCCTG
**Ccl11**	NM_011330.3	CAACCTCCTCTCTTGACACTAACCCAGAGCCTAAGAACTGCTTGATTCCTTCTCTTTCCTAAGACGTGCTCTGAGGGAATATCAGCACCAGTCGCCCAAG
**Ccl12**	NM_011331.2	ATGAAGATTTCCACACTTCTATGCCTCCTGCTCATAGCTACCACCATCAGTCCTCAGGTATTGGCTGGACCAGATGCGGTGAGCACCCCAGTCACGTGCT
**Ccl17**	NM_011332.2	TCTGTGCAGACCCCAAAGACAAACATGTGAAGAAGGCCATCAGATTGGTGAAAAACCCAAGGCCATGACCTTCCCGCTGAGGCATTTGGAGACGCCAGGG
**Ccl19**	NM_011888.2	CTTCTGCCAAGAACAAAGGCAACAGCACCAGAAGGAGCCCTGTGTCTTGAGTAAAGAGATGTGAATCACTCTGGCCCAGGAAACCAAGGACCAGAAGAGA
**Ccl2**	NM_011333.3	TCTTCAGCACCTTTGAATGTGAAGTTGACCCGTAAATCTGAAGCTAATGCATCCACTACCTTTTCCACAACCACCTCAAGCACTTCTGTAGGAGTGACCA
**Ccl20**	NM_016960.1	GCAGCAAGCAACTACGACTGTTGCCTCTCGTACATACAGACGCCTCTTCCTTCCAGAGCTATTGTGGGTTTCACAAGACAGATGGCCGATGAAGCTTGTG
**Ccl21a**	NM_011124.4	TTAAGTACAGCCAGAAGAAAATTCCCTACAGTATTGTCCGAGGCTATAGGAAGCAAGAACCAAGTTTAGGCTGTCCCATCCCGGCAATCCTGTTCTCACC
**Ccl22**	NM_009137.2	CCAAGAATCAACTTCCACCCCTCTTCAACCACATGCTAGGGTCTTTTACTTTCTCTGCCCCACACCTTTGACTCCTTGCCTGTGTAGCTGATAGTCGAAG
**Ccl24**	NM_019577.4	CTGGATGCCAAGAAAAACCAGCCTTCTAAAGGGGCCAAGGCAGTGAGAACCAAGTTTGCTGTCCAGAGACGCCGTGGCAATAGCACCGAGGTTTAGCCGC
**Ccl25**	NM_009138.3	TTCCGCCAGAAAGTAGTGTGTGGGAATCCAGAGGACATGAATGTGAAGAGGGCGATGAGAATCTTGACAGCTAGGAAAAGGCTAGTCCACTGGAAGAGCG
**Ccl26**	NM_001013412.2	CAGCTATAAGTTCACCGACAAGAGCTGCACCAGTGACGGTGTGATATTCTTTACAAAAACAGGTAAGCAATTCTGTGTCCAGCCAGGGGCCAAATGGGTG
**Ccl27a**	NM_001048179.1	GCCTCTGCCCTCCAGCACTAGCTGCTGTACTCAGCTCTATAGACAGCCACTCCCAAGCAGGCTGCTGAGGAGGATTGTCCACATGGAACTGCAGGAGGCC

**Ccl28**	NM_020279.3	GCTGCTGTCATCCTTCATGTTAAACGTAGAAGAATCTGCATCAGCCCGCACAATCGTACTTTGAAGCAGTGGATGAGAGCCTCAGAGGTAAAGAAGAATG
**Ccl3**	NM_011337.1	TCTGTCACCTGCTCAACATCATGAAGGTCTCCACCACTGCCCTTGCTGTTCTTCTCTGTACCATGACACTCTGCAACCAAGTCTTCTCAGCGCCATATGG
**Ccl4**	NM_013652.1	TTCTCAGCACCAATGGGCTCTGACCCTCCCACTTCCTGCTGTTTCTCTTACACCTCCCGGCAGCTTCACAGAAGCTTTGTGATGGATTACTATGAGACCA
**Ccl5**	NM_013653.3	GTCGTGTTTGTCACTCGAAGGAACCGCCAAGTGTGTGCCAACCCAGAGAAGAAGTGGGTTCAAGAATACATCAACTATTTGGAGATGAGCTAGGATAGAG
**Ccl6**	NM_009139.2	ATGTAGCCACAGGACTGTCTCTTTCTTCAGATTCATAGAACTACGGGGCCAGTCTGAAACTGGGCTCTTGGGGTGAAATTATCTCACTCACTCACCTTGA
**Ccl7**	NM_013654.2	ACAGAAGGATCACCAGTAGTCGGTGTCCCTGGGAAGCTGTTATCTTCAAGACAAAGAAGGGCATGGAAGTCTGTGCTGAAGCCCATCAGAAGTGGGTCGA
**Ccl8**	NM_021443.2	ACCTGCTGCTTTCATGTACTAAAGCTGAAGATCCCCCTTCGGGTGCTGAAAAGCTACGAGAGAATCAACAATATCCAGTGCCCCATGGAAGCTGTGGTTT
**Ccl9**	NM_011338.2	ATGGCAACCCAGGTACATTCAACTAGGATGAAATAAATTCTGCCTTAGCCCAGTAGTATGTCTGTGTTTGTAAGGACCCAGCTGATTTTCCCACCACCCC
**Ccnd3**	NM_007632.2	GCCATGATGGTCAGAGAAATACAAACAGGTAAAATCCACACACCAGCATTTCTTTTGAGTCCCTCTTCTGTCCGGGGCTCCAACCTTCTCAGTTGCCAAA
**Ccr1**	NM_009912.4	CTTCTGATTCAGACCATAGGTGTCAACCAAGGAAGGTCTAAGAAGAGAATGAGGAGACAGTATATAGCTCTCCAAGACTGATACTGACAGTTCTTACAGT
**Ccr2**	NM_009915.2	ATGAACTAACATAGACAGCTCAGGATTAACAGGGACTTGTGGTTTGTGGTCTGTGGGCTTATCCAAGCATGGTGATTTAGACTCTAAGGTCCGTCTGGAT
**Ccr3**	NM_009914.4	TGACTTTTGCTACTATCACCAGTATCATTACCTGGGGCCTTGCAGGACTGGCAGCATTGCCTGAATTTATCTTCCATGAGTCTCAAGACAGCTTTGGAGA
**Ccr4**	NM_009916.2	CTACTACGCCGCCGACCAGTGGGTTTTTGGACTAGGTCTGTGCAAGATCGTTTCATGGATGTACCTGGTGGGCTTCTACAGCGGCATCTTCTTCATCATG
**Ccr5**	NM_009917.5	GGAGCAGGGAGAACGAGTCTTTTATCAGGGCCGGGAAATATGCACAAAGAGACTTGAGGCAGGTGCCATGACCCATATGCAAAGGGACGGACACAGGGCC
**Ccr6**	NM_001190333.1	CTACCGTTCTGGGCAGTTACTCATGCCACCAACACTTGGGTTTTCAGCGATGCACTGTGTAAACTGATGAAAGGCACATATGCGGTCAACTTTAACTGTG
**Ccr7**	NM_007719.2	CCCAGATGGTTTTTGGGTTCCTAGTGCCTATGCTGGCTATGAGTTTCTGCTACCTCATTATCATCCGTACCTTGCTCCAGGCACGCAACTTTGAGCGGAA
**Ccr8**	NM_007720.2	GTTCTTCATCACCCTAATGAGTGTGGACAGGTATCTGGCTATTGTCCACGCTGTCTATGCCATCAAGGTGAGGACGGCCAGCGTGGGCACAGCCCTGAGT
**Ccr9**	NM_009913.6	TATACCATCATCATTCATACCTTGGTACAGGCCAAGAAGTCATCCAAGCACAAGGCCCTCAAGGTGACCATCACTGTCCTCACTGTCTTCATTATGTCTC
**Ccrl2**	NM_017466.4	TGTTTGCGGTGGGTCTCTTGGACAACGTGCTGGCGGTGTTTATCTTGGTGAAATACAAAGGACTCAAGAATCTGGGGAACATCTACTTCCTAAACCTGGC
**Cd14**	NM_009841.3	CTAGACGAGGAAAGTTGTTCCTGCAACTTCTCAGATCCGAAGCCAGATTGGTCCAGCGCTTTCAATTGTTTGGGGGCGGCAGATGTGGAATTGTACGGCG
**Cd160**	NM_001163496.1	CAACTTAGAACAGCTTAGGGTTAAAAGGGATCCTGAGACAGATGGCATCACTGAAAAGTCATCTCAGTTGGTGTTCACCATAGAACAAGCTACACCATCA
**Cd163**	NM_053094.2	TCACGGCACTCTTGGTTTGTGGAGCCATTCTATTGGTCCTCCTCATTGTCTTCCTCCTGTGGACTCTGAAGCGACGACAGATTCAGCGACTTACAGTTTC
**Cd164**	NM_016898.2	ATATTGAAGAAGTCCCACATGGAAGACGCTGTTCCAGGATCTTTACCTTTCCAAAGGATGCATACAGGATATTTTGGAGCATCATCCTCGAAGAAAAGTC
**Cd180**	NM_008533.2	AGGGTTTGAAGAACTCTACGATCCAGTCTCTTTGGCTGGGGACATTTGAGGACATGGATGACGAAGATATTAGTCCTGCCGTGTTTGAGGGTCTCTGTGA
**Cd19**	NM_009844.2	CTCAGCGTTGGGCTGCTGGCCTAGGGAGTGTCCCTGGGTCCTATGGAAATCCACGCATTCAAGTCCAGGATACTGGAGCTCAGAGCCATGAAACAGGACT
**Cd1d1**	NM_007639.3	GTTTTATGGGATTTGCACTGAACTAGAAAGCATACTTCCTGCCCAAACAGACGCTCTGAGGTTAGTTGGCAAGTGTAAAGTCCAACACCAACCTGGCTGT

**Cd1d2**	NM_007640.2	GGAATGCTTCGGAAAGCTTTTTTCATGTAGCATTTCAAGGAAAATATGCCGTGAGATTCCGGGGAACATCTTGGCAGAGGGTCCTAGGGGCCCCATCTTG
**Cd2**	XM_006500959.1	ACTGTTTTCCTGTTACTCTTCCCTATTCTGGTGGAAGAAGGGATCAGAACTCTTATGCCACTGATAACTGTTGAGTGTCTAGGCGGCTTTCCTGAACCCA
**Cd200**	NM_010818.3	CCCTGCCATCTCCTGGAAGGGTACTGGGACAGGAATTGAGAATAGTACCGAGAGTCACTTCCATTCAAATGGGACTACATCTGTCACCAGCATCCTCCGG
**Cd200r1**	NM_021325.3	AAGCTACTTCAGCTATTGAGGAGGATGAAATGCAGCCTTATGCTAGCTATACAGAGAAGAGCAATCCACTCTATGATACTGTGACTAAGGTGGAGGCATT
**Cd207**	NM_144943.3	ATGTCAACAAGCTGCTCAAACAACAGAGTGACATTCTGGAGATGGTGGCTCGAGGCTGGAAGTATTTCTCGGGGAACTTCTATTACTTTTCACGCACCCC
**Cd209e**	NM_130905.2	CCAGTTGGGGAGTGGACTTTCACTCATGCGTTTCCCAGTTCCTACTCCCTGTCTATGGGCATGGAAATCTTAGTATTACCTCTGGGACTCATCAGTCTCC
**Cd22**	NM_001043317.2	CTCTGACCTGTGGACTGAATTTCTCCTGCTTTGGGTATGACATCCTCTTGAAGTGGTTCCTAGAAGATTCTGAGATCACCTCCATCACCTCTTCTGTCAC
**Cd244**	NM_018729.2	TACAGACAAAAGATGTTTCTGTTCAATGGAAGAAGACAGAACAGGGCTCACACAGAAAAATTGAGATCCTGAATTGGTATAATGATGGTCCCAGTTGGTC
**Cd247**	NM_0011133391.2	GCTTTGGTCTGCTGGATCCCAAACTCTGCTACTTGCTAGATGGAATCCTCTTCATCTACGGAGTCATCATCACAGCCCTGTACCTGAGAGCAAAATTCAG
**Cd27**	NM_001033126.2	CTGGGGGAGGACTCTGCTGCCGGATGTGTGAGCCAGGTACATTCTTTGTGAAGGACTGTGAACAAGACAGAACAGCTGCTCAGTGTGATCCCTGTATACC
**Cd274**	NM_021893.2	TGAACTAATATGTCAGGCCGAGGGTTATCCAGAAGCTGAGGTAATCTGGACAAACAGTGACCACCAACCCGTGAGTGGGAAGAGAAGTGTCACCACTTCC
**Cd276**	NM_133983.4	TGGTCCATAGACCACCCACAGCCTTACTTTTCCAATGGACTTAATTCCCATCATCCTGCAGCCTCATTTCTCCAGTGACACGATACACGAACCATCCTGC
**Cd28**	NM_007642.4	TCTGTCTCTCTCTTTCTCTCCTGTGCATATGTCTCCCCTCCCTCACTTCTCTGTCTCTTCCTCTCTCCCTCTCTCTGTCTTTCTCTGTGTGTCTCTCTGT
**Cd33**	NM_001111058.1	ATGACACAGGAATGTACTTCTTCAGAGTGGTCAGAGAACCTTTTGTGAGATATTCTTACAAAAAAAGCCAGCTGTCACTGCATGTGACCTCTCTATCACG
**Cd34**	NM_001111059.1	CTGATTATTCGCCTAATAATAGCAGCTTTGAGATGACATCACCCACCGAGCCATATGCTTACACATCATCTTCTGCTCCGAGTGCCATTAAGGGAGAAAT
**Cd36**	NM_007643.3	GGGACCATTGGTGATGAAAAAGCAGAAATGTTCAAAACACAAGTGACTGGGAAAATCAAGCTCCTTGGCATGGTAGAGATGGCCTTACTTGGGATTGGAG
**Cd37**	NM_007645.4	GTACTAGGCGGCCTGATTTTCTGCTTCGGCACCTGGATCCTCATTGACAAGACCAGCTTCGTGTCCTTTGTGGGTTTGTCCTTCGTGCCACTGCAGACTT
**Cd38**	NM_007646.4	TATTCACATGGTAAACCTGAGGTCATAGGGTCATTATAGGGAAGGTGCTGTGTGGGAACTACCCACGTGCCCTGTGCTTTAATCTTTAACTCAACACATC
**Cd3d**	NM_013487.2	CTGTGTGGAGCTAGACTCGGGCACCATGGCTGGTGTCATCTTCATTGACCTCATCGCAACTCTGCTCCTGGCTTTGGGCGTCTACTGCTTTGCAGGACAT
**Cd3e**	NM_007648.4	TGAGTACTGTGTGGAGGTGGACCTGACAGCAGTAGCCATAATCATCATTGTTGACATCTGTATCACTCTGGGCTTGCTGATGGTCATTTATTACTGGAGC
**Cd3eap**	NM_145822.2	ACCTGTCATTAAAGCTGTCGCTGGCCCGCCTGTTTGGAATGGGGTTCTGCCTCTTATTCTCCTGGCTGAGGAGCTTCGGGTGTCTGGGTTCTACATGAGG
**Cd3g**	NM_009850.2	GCATTGAGCTAAACATAGGCACCATATCCGGCTTTATCTTCGCTGAGGTCATCAGCATCTTCTTCCTTGCTCTTGGTGTATATCTCATTGCGGGACAGGA
**Cd4**	NM_013488.2	AAGAGGTGTCCGTACAAAAGTCCACCAAAGACCTCAAGCTCCAGCTGAAGGAAACGCTCCCACTCACCCTCAAGATACCCCAGGTCTCGCTTCAGTTTGC
**Cd40**	NM_011611.2	GCTGCCCAAACCCGGTTACCTGATTTTGATCCCTGGGACTTCATGGTAAAAGGGAGAGAACCAAATCCAGAGGGTTGTCATTTGACCTCCATGTGTGCTC
**Cd40lg**	NM_011616.2	AGCAGTGGATCTGAGAGAATCTTACTCAAGGCGGCAAATACCCACAGTTCCTCCCAGCTTTGCGAGCAGCAGTCTGTTCACTTGGGCGGAGTGTTTGAAT
**Cd44**	NM_009851.2	TCGCTTTGGGTGTGTCCTTCGCGCGCTCCCTCCCTCTTAGGTCACTCACTCTTTCAAAGCCTGGAATAAAAACCACAGCCAACTTCCGAAGCGGTCTCAT

**Cd46**	NM_01077 8.3	TGAGGCCCTTCTGTTTCTGCTGTCTCATTTATCTGATGCCTGTGAACTACCACGGCCATTTGAAGCT ATGGAACTCAAGGGTACACCTAAACTCTTTTAT
**Cd47**	NM_01058 1.3	CTCCTGCTGCTGCGGTTCAGCTCAACTACTGTTTAGTAACGTCAACTCCATAGAGTTCACTTCATGC AATGAAACTGTGGTCATCCCTTGCATCGTCCGT
**Cd48**	NM_00764 9.4	TGCGTGAAACTGAGAACGAGTTGAAGATAACCCTGGAAGTATTTGATCCTGTGCCCAAGCCTTCC ATAGAAATCAATAAGACTGAAGCGTCGACTGATTC
**Cd5**	NM_00765 0.3	ACCAGAACATGTCTTTCCATCGAAGCCACACAGCAACTGTGCGGTCCCAAGTTGAGAACCCAACA GCCTCTCATGTGGACAATGAATACAGCCAGCCTCC
**Cd53**	NM_00765 1.3	ATGGCATCAGTTATTCTGGGTCAAAAGTTCAATTTCCACCCAGGGCAAAATTACACAACAGGCATA TGACTTCTAGGAAGTTGAGATCATTGATGGCCAC
**Cd55**	NM_01001 6.2	ACAGTTAAAGTTTCAGCAACCCAGCATGTACCTGTTACCAAGACAACAGTACGTCATCCAATAAGA ACATCTACAGACAAAGGAGAGCCTAACACAGGTG
**Cd59b**	NM_18185 8.1	CGATTAGACGTGGCAGGCATACAAAGCAAATGCTGTCAGTGGGACTTGTGTAACAAAAATCTTGA CGGATTAGAAGAGCCAAACAACGCAGAAACTTCCT
**Cd6**	NM_00103 7801.2	GAAGTGCCTTCCAGAGTTCCAGTCACGATAGAATCTTCTGTGCCGGTGAGCGTGAAGGATAAGGA CTCACAAGGGCTGACACTTCTCATTCTCTGTATTG
**Cd63**	NM_00104 2580.1	GTGGGATTGATCGCCATTGGTGTAGCGGTTCAGGTTGTCTTGAAGCAGGCCATTACCCATGAGAC TACTGCTGGCTCGCTGTTGCCTGTGGTCATCATTG
**Cd68**	NM_00985 3.1	AGCCCGAGTACAGTCTACCTGGACTACATGGCGGTGGAATACAATGTGTCCTTCCCACAGGCAGC ACAGTGGACATTCATGGCGCAGAATTCATCTCTTC
**Cd69**	NM_00103 3122.3	GCAGATTTCAAAGTGCTGGAAAGAAAAGTGCAATACGTGTAGTGGCAGATCTCTGTCAGGACAC ACCCTGTGGTTTGACCTTGGAATAACTCCAGTCTTG
**Cd7**	NM_00985 4.1	CTCAGGCTTACAATGTGATTTACTTTGAAGACCGGCAGGAGCCCACAGTAGACAGGACCTTCTCA GGCCGAATTAATTTCTCTGGTTCCCAGAAGAACCT
**Cd70**	NM_01161 7.1	CGCCTGACATACCTGGTCCACGGAGATGTCCTCTGTACCAACCTCACCCTGCCTCTGCTGCCGTCC CGCAACGCTGATGAGACCTTCTTTGGAGTTCAGT
**Cd74**	NM_00104 2605.1	ATGGATAACATGCTCCTTGGGCCTGTGAAGAACGTTACCAAGTACGGCAACATGACCCAGGACCA TGTGATGCATCTGCTCACGAGGTCTGGACCCCTGG
**Cd79a**	NM_00765 5.3	AAATAACAGGAAGTCTCTTGGGGAAGTCCCCACCCCAATTGAAATGCTGGTTTATCTCCCTTATAG GGCCTCACCCTCCTCATGTCCCAAAGCAAGGGGG
**Cd79b**	NM_00833 9.2	CAGACCCAGAATGGCTCTGTCTACACCCTCACTATCCAAAACATCCAGTACGAGGATAATGGTATC TACTTCTGCAAGCAGAAATGTGACAGCGCCAACC
**Cd80**	NM_00985 5.2	TGGCTTTCCCCATCATGTTCTCCAAAGCATCTGAAGCTATGGCTTGCAATTGTCAGTTGATGCAGG ATACACCACTCCTCAAGTTTCCATGTCCAAGGCT
**Cd81**	NM_13365 5.2	GGCATCTGGGGCTTCGTAAACAAAGACCAGATCGCCAAGGATGTGAAGCAGTTCTATGACCAGG CCCTTCAGCAAGCTGTGATGGATGATGATGCCAACA
**Cd83**	NM_00985 6.2	CCAGATAGTTTAGTCTGGGTTGAATACAATGGATGTGAAGTTGCTTGGGGAAAGCTGAATGTAGT GAATACATTGGCAACTCTACTGGGCTGTTACCTTG
**Cd84**	NM_00125 2472.1	CGCCATCTGTGGATCTGGTTCCTTTGCCTACAAACCTGGTCTGAAGCAGCAGGAAAAGATGCAGA CCCGGTGGTAATGAATGGGATTCTTGGGGAGTCAG
**Cd86**	NM_01938 8.3	CAAAACATAAGCCTGAGTGAGCTGGTAGTATTTTGGCAGGACCAGCAAAAGTTGGTTCTGTACGA GCACTATTTGGGCACAGAGAAACTTGATAGTGTGA
**Cd8a**	NM_00108 1110.2	CCACCTTCGTTGTCTATATGGCTTCATCCCACAACAAGATAACGTGGGACGAGAAGCTGAATTCGT CGAAACTGTTTTCTGCCATGAGGGACACGAATAA
**Cd8b1**	NM_00985 8.2	GCCACCTCATGAATAAAGTTTTCTGCAAACAGTTTCAGGGCTCTCAGCAGCCATCTTACTCTCTCAG GCCTTGCCGTGGGTTTGGGAGGCTGTTGGCTCA
**Cd9**	NM_00765 7.3	CAGCTTTTGGAAAGTTTCCAGGTTAAGCCCTGCCCTGAAGCCATCAGTGAGGTCTTCAACAACAA GTTCCACATCATTGGAGCAGTGGGTATCGGCATCG
**Cd96**	NM_03246 5.2	AACCCTGTGGTAGAGTTGAGGTTTTTGAGCACGAATAGGGTGGCTAGCGTTTGCTTAGCTAGAAA CAGCAATCGCCTCCTGTGATCATTACAGAAATGCT
**Cd97**	NM_01192 5.1	CGTGGCCATCTCAATGGCAGTTGTCAAAATGGATGGCTATGGGCATGCAACATACTGCTGGCTGG ACTTTAGGAAGCAGGGCTTTCTCTGGAGCTTTTCG

**Cd99**	NM_025584.2	CCGCCGTGAAATCACCGCCTGCACCGCCCCTCGCCGCTGCATCAGTGATGTCACTACTGCCAAAGACTCCGCCCACAACTGACCTCTGACCCCGGTGACA
**Cdh1**	NM_009864.2	TCGAAGTGCCCGAAGACTTTGGTGTGGGTCAGGAAATCACATCTTATACCGCTCGAGAGCCGGACACGTTCATGGATCAGAAGATCACGTATCGGATTTG
**Cdh5**	NM_009868.3	CAGCAACTTCACCCTCATAAACAACCATGACAACACCGCCAACATCACGGTCAAGTATGGGCAGTTTAATCGGGAGCATGCCAAGTTCCACTACCTGCCC
**Cdk1**	NM_007659.3	CCAGATCTCTGTTGGGAAGTAACTGGGTAACAAAAGCCGTTGCACTGGTTTGACAGCTAACAACTGTTGGTACTTTGTATTCAGAAGGAATGAGGTAGCG
**Cdkn1a**	NM_007669.4	AATACCGTGGGTGTCAAAGCACTTAGTGGGTCTGACTCCAGCCCCAAACATCCCTGTTTCTGTAACATCCTGGTCTGGACTGTCTACCCTTAGCCCGCAC
**Ceacam1**	NM_001039185.1	CAAGGGAAACACTACGGCTATAGACAAAGAAATTGCACGATTTGTACCAAATAGTAATATGAATTTCACGGGGCAAGCATACAGCGGCAGAGAGATAATA
**Cebpb**	NM_009883.3	CGGGCACCGCGCGCACGCACCTGCACAGCGCACCGGGTTTCGGGACTTGATGCAATCCGGATCAAACGTGGCTGAGCGCGTGTGGACACGGGACTGACGC
**Cfb**	NM_008198.2	GGTCGGGCCTCTGGTGGACTCCGTGAACATCAATGCCTTAGCTTCCAAAAAGGACAATGAGCATCATGTGTTTAAAGTCAAGGATATGGAAGACCTGGAG
**Cfd**	NM_013459.1	AGTGTCAATCATGAACCGGACAACCTGCAATCTGCGCACGTACCATGACGGGGTAGTCACCATTAACATGATGTGTGCAGAGAGCAACCGCAGGGACACT
**Cfh**	NM_009888.3	GAAATTCTCTGCACACCACCGCGAGTGGAAAATGGAGATGGTATAAATGTGAAACCAGTTTACAAGGAGAATGAAAGATACCACTATAAGTGTAAGCATG
**Cfi**	NM_007686.2	TTCCTTAATTTATGGGAGAACAAAAACAGAGGGACTTGTTCAAGTCAAACTTGTGGACCAAGATGAGAGAATGTTCATATGTAAAAACAGCTGGAGCATG
**Cfp**	NM_008823.3	CGGCCGCAAATTTAATGGGAAGCCATGTGCTGGAAAACTCCAGGATATTCGACACTGCTATAACATCCATAACTGTATCATGAAAGGTTCATGGTCACAG
**Chil3**	NM_009892.2	ATTGTGGGATTTCCAGCATATGGGCATACCTTTATCCTGAGTGACCCTTCTAAGACTGGAATTGGTGCCCCTACAATTAGTACTGGCCCACCAGGAAAGT
**Chit1**	NM_027979.1	GGAGGCAGTTCGGTTCTTTCCCAGGGATGTGGATCCCAACCTGTGTACCCACGTCATCTTTGCTTTTGCTGGAATGGACAACCATCAGCTCAGCACTGTG
**Chuk**	NM_001162410.1	TCGTTTAGAGCTAAGTTCCAAAAACAGAGAGCGATGGTGCCATGAAATCCAGATCATGAAAAAGTTGGACCATGCGAATGTTGTAAAGGCCTGTGATGTC
**Ciita**	NM_007575.2	TGCAGCCACTGGCCATGGATGACGAGGTCCTTGATTATATCGTGAGGCAGCCAGACCGTGTTCTGCTCATCCTAGATGCTTTCGAGGAGCTAGAGGCCCA
**Cklf**	NM_029295.2	GAAAGAGCCATTAATCGAGAAGGCTTGAAAGAGAGGCGTGACTGTTGGGTCTGTGGGCAAGATGGAGACTCCACGGCCGGTCGTAAGCCGTCGGCCCTTC
**Clec4a2**	NM_001170332.1	ATGGGCATTCGATTCATTGTTATCCAACCATTACACAGACACCTGGGAAATTCTACAGGTTCACAGAATTTAAGTGGGCAGCAAATGGTTATGCATACAC
**Clec4n**	NM_001190320.1	CTGAAGCGGAGCAGAATTTCATCACCCAGCAGCTGAATGAGTCACTTTCTTACTTCCTGGGTCTTTCGGATCCACAAGGTAATGGCAAATGGCAATGGAT
**Clec5a**	NM_001038604.1	CGCTGGATCAACAACTCTGTGTTCAATGGCAATGTTACCAATCAGGACCAGAACTTCGACTGTGTCACTATAGGTCTGACGAAGACATATGATGCTGCAT
**Clec7a**	NM_020008.2	GGATGAAGAAGCTGAGACTTTTGTACTTGTCATCTTCACAAAGATGGTGGCACTATCTTCCAGTTAGGAAGTCACTAGACATGGAGTGAGGGCAGCTCAA
**Clu**	NM_013492.2	TTAATAAGGAGATTCAGAACGCCGTCCAGGGAGTGAAGCACATAAAAACTCTCATAGAAAAAACCAACGCAGAGCGCAAGTCCTTGCTCAACAGTTTAGA
**Cma1**	NM_010780.2	GAGGGAGAATTAACTCTGGAGCTTTTGCCAGCCTGTGAGGAAATCTGGAACTGGAATAGTGCAGGTTTTGTGTGCCATGCGATCTGGCCTGTCTGTAGTT
**Cmah**	NM_001111110.2	CATCGAGGATTTAGATGGAAGGTCCGTTAAATGCACAAAGCACAACTGGAAGTTAGACGTGAGCACCATGAAATATATCAACCCTCCAGGGAGCTTCTGT
**Cmklr1**	NM_008153.3	TCGCCACCTTCAAGATGAAGAAGACCGTGAACACTGTGTGGTTTGTCAACCTGGCTGTGGCCGACTTCCTGTTCAACATCTTTTTGCCGATGCACATCAC
**Cmpk2**	NM_020557.4	AAAAACTGGGGACTGAGCAGTTTGCTTTCCTTCTGACTGACTGAGCCAGCTGCGGCTGCTGATTCATTTCTGCCTGGCCATGTACCAACTTCTTGGGATA

**Col1a1**	NM_007742.3	CAATGGTGAGACGTGGAAACCCGAGGTATGCTTGATCTGTATCTGCCACAATGGCACGGCTGTGTGCGATGACGTGCAATGCAATGAAGAACTGGACTGT
**Col3a1**	NM_009930.1	CAAGACAGTCTTTGAATATCAAACACGCAAGGCAATGAGACTACCCATCATAGATATCGCACCCTATGACATTGGGGGTCCTGATCAAGAATTTGGTGTG
**Col4a1**	NM_009931.2	CTCCAGGGATCACAGGTTCAAAGGGAGATATGGGACTGCCCGGAGTTCCAGGATTTCAAGGTCAGAAAGGGCTTCCTGGTCTGCAGGGAGTGAAAGGAGA
**Colec12**	NM_130449.2	TAACCCAGGTTCAGCAGAGGAACCTTATCTCAAATCTGCAGCAGTCTGTGGATGACACAAGCCTGGCCATCCAGCGAATTAAGAATGATTTCCAAAATCT
**Cr2**	NM_007758.2	TGGTTTATAGAAATCCGTCTTTGTAAAGAAATCACCTGCCCACCACCTCCTGTTATACACAACGGGACACATACATGGAGTTCCTCAGAAGATGTCCCAT
**Creb1**	NM_001037726.1	TTCTGTCTGGACAGTTCACCAGATTCTCCAGAAGGCTTTCAAACGGCTAAAGTTTGATCTTTGTCCTGCTGAGCTTGCTGGGAAGGAGATAGCATAAAAG
**Creb5**	NM_172728.2	CATGGAGGTGGCCAGGCTGGTGCGCTCATCTGAGTAGTTCTGATTTATATTTTCAGCAATGTCCACGGACTTGCCCATTACGGAAAGCAGATCAAACCCA
**Crebbp**	NM_001025432.1	TTGGATATTGCTGTGGACGAAAGTATGAGTTCTCCCCACAGACTTTGTGCTGTTACGGAAAGCAGCTGTGTACAATTCCTCGTGATGCAGCCTACTACAG
**Crp**	NM_007768.4	TTGTATTTCCCAAGGAGTCAGATACTTCCTATGTGTCTCTGGAAGCAGAGTCAAAGAAGCCACTGAACACCTTTACTGTGTGTCTCCATTTCTACACTGC
**Csf1**	NM_001113530.1	TCCAGCTGCTGGAGAAGATCAAGAACTTCTTTAATGAAACAAAGAATCTCCTTGAAAAGGACTGGAACATTTTTACCAAGAACTGCAACAACAGCTTTGC
**Csf1r**	NM_001037859.2	GTACAAGCAGAAGCCGAAGTACCAGGTGCGCTGGAAGATCATCGAGAGATACGAAGGCAATAGCTACACCTTCATTGACCCTACTCAGTTGCCCTACAAT
**Csf2**	NM_009969.4	CTGAATCCAGCTTCTCAGACTGCTGCTTTTGTGCCTGCGTAATGAGCCAGGAACTTGGAATTTCTGCCTTAAAGGGACCAAGAGATGTGGCACAGCCACA
**Csf2rb**	NM_007780.4	ATTGCGTGTCTGCATTGCTATGAGATGGGTTGCCGAGCCACACCTGTCTTTGGGTCCCTGTTTGCAGGGGTCTTTGCTGTAAGTGCACACGTGCCTTTGT
**Csf3**	NM_009971.1	TTGTTCTCCTGCTTAGAGCAGAGAGAGAAGGCTCTTGTGTCCTCCTGTGGAGGCCAGGGAAGGAGATGGGTAAATACCAAGTATTGATTCCTGCTGCTGC
**Csf3r**	NM_001252651.1	CCCAGCCTTCTGCCTCAGGGCTATCTCATTGAGTGGGAAATGAGTTCTCCCAGCTACAATAACAGCTATAAGTCCTGGATGATAGAACCTAACGGGAACA
**Cspg4**	NM_139001.2	TGGAGCTAGACATCCTGGGAGCCCAAACCCGGAAAATGTTTACCCTGTTGGACGTGGTGAACCGTAAGGCCCGCTTTGTTCACGATGGCTCTGAAGACAC
**Ctla4**	NM_009843.3	TGGACTCGAGGTCCTGCACCAACTGGCTTGGAAACTAGATGAGGCTGTCACAGGGCTCAGTTGCATAAACCGATGGTGATGGAGTGTAAACTGGGTCTTT
**Ctsg**	NM_007800.1	CTATGGAAGCAACAATGGTAACCCTCCAGCTGTATTCACCAAAATCCAGAGCTTCATGCCCTGGATCAAAAGAACAATGAGACGCTTTGCACCAAGATAT
**Ctsh**	NM_007801.2	TGTCATAAAACTCCAGATAAAGTAAACCATGCAGTCCTGGCGGTTGGCTATGGAGAACAGAATGGATTACTCTACTGGATTGTGAAAAACTCTTGGGGCT
**Ctsl**	NM_009984.3	TTCTTGTGCGCAGCTAGCCGCCTCAGGTGTTTGAACCATGAATCTTTTACTCCTTTTGGCTGTCCTCTGCTTGGGAACAGCCTTAGCTACTCCAAAATTT
**Ctss**	NM_021281.2	AGACGCTTCCTATCCCTACAAAGCCACGGATGAAAAGTGTCACTATAACTCAAAAAATCGAGCTGCCACGTGTTCAAGGTACATTCAGCTCCCGTTTGGT
**Ctsw**	NM_009985.4	GCCGTCTGAGCATCTTTGCCCACAATCTGGCTCAGGCTCAAAGGCTACAGCAAGAAGACTTGGGTACAGCTGAGTTTGGAGAGACTCCATTCAGTGACCT
**Cx3cl1**	NM_009142.3	CGTTCTTCCATTTGTGTACTCTGCTGCCGGGTCAGCACCTCGGCATGACGAAATGCGAAATCATGTGCGACAAGATGACCTCACGAATCCCAGTGGCTTT
**Cx3cr1**	NM_009987.3	TCCTGTGTTGGTTGTGATAACCATTTCAGAAGTCTCTCCCAGCCTGTTGCTCACGGCGGCATGTCTGATATCTCCTTGGCAGTCTGTATGTTTGTGTCGA
**Cxcl1**	NM_008176.1	TGCTAGTAGAAGGGTGTTGTGCGAAAAGAAGTGCAGAGAGATAGAGTTTAGTATTATGTTTTGTATGTATTAGGGTGAGGACATGTGTGGGAGGCTGTGT
**Cxcl10**	NM_021274.1	AGGACGGTCCGCTGCAACTGCATCCATATCGATGACGGGCCAGTGAGAATGAGGGCCATAGGGAAGCTTGAAATCATCCCTGCGAGCCTATCCTGCCCAC

**Cxcl11**	NM_01949 4.1	TTAAGGCGTCAAAACATGTGACATCCTGGGAACGTCTGACTGTGAGCCCTCCAATAAGAACTCTG TGCCAGGAACCTGACCCTCTGCTGTCTTGGAACAT
**Cxcl12**	NM_02170 4.3	CTGAAAATCCTCAACACTCCAAACTGTGCCCTTCAGATTGTTGCACGGCTGAAGAACAACAACAGA CAAGTGTGCATTGACCCGAAATTAAAGTGGATCC
**Cxcl13**	NM_01886 6.2	CTGAGAGGGAATGCTCAAGCTCCGTTGCATACCCAACCCACATCCTTGTTCCTTAAGAAAGGCTAT TTGAGAACAGGCATTTAGTGACAACCCACTTCAG
**Cxcl14**	XM_006517 307.1	GTGGACGGGTCCAAGTGTAAGTGTTCCCGGAAGGGGCCCAAGATCCGCTACAGCGACGTGAAGA AGCTGGAAATGAAGCCAAAGTACCCACACTGCGAGG
**Cxcl15**	NM_01133 9.2	AGAGAATATTTCCCTTTCCAATTCGGGAGACCTCTAGACACTTTGCTGATTTAGCTCACAACAGTG ATAGGAATTTTCTACGGGACTCCAGTGAAGTCAG
**Cxcl16**	NM_02315 8.6	CGCAGGGTACTTTGGATCACATCCGAAAATACCTGAAAGCATTTCATCGTTGTCCATTCTTTATCA GGTTCCAGTTGCAGTCCAAAAGCGTGTGTGGGGG
**Cxcl2**	NM_00914 0.2	GGTGGGGGTGGGGACAAATAGATGCAGTCGGATGGCTTTCATGGAAGGAGTGTGCATGTTCACA TCATTTTTTTGTAAGCACCGAGGAGAGTAGAACAGC
**Cxcl3**	NM_20332 0.2	ACTCTCAAGGATGGTCAAGAAGTTTGCCTCAACCCCCAAGGCCCCAGGCTTCAGATAATCATCAA GAAGATACTGAAGAGCGGCAAGTCCAGCTGAGCCG
**Cxcl5**	NM_00914 1.2	CCCAGTGAAGATAAGAAGAAAGGGCTGATTCTCTCCACCCACGGATTTTCTTTATGAACTCCCTGC TTTGATGAGAAAAGGGAAACCATTGTCCCTGAAG
**Cxcl9**	NM_00859 9.2	TAGAACTCAGCTCTGCCATGAAGTCCGCTGTTCTTTTCCTCTTGGGCATCATCTTCCTGGAGCAGTG TGGAGTTCGAGGAACCCTAGTGATAAGGAATGC
**Cxcr1**	NM_17824 1.4	TCTCTTAGGAGCCCACTTGATTGAAGATACTTGCGAACGCCGCAATGACATTGACCAGGCCCTGTA TATTACTGAGATCCTGGGCTTTTCTCATAGTTGT
**Cxcr2**	NM_00990 9.3	CCTTGCCTGTCTGGGCTGCATCTAAAGTAAATGGATGGACTTTTGGCTCAACCCTGTGCAAGATAT TCTCATACGTGAAGGAGGTTACCTTCTACAGCAG
**Cxcr3**	NM_00991 0.2	GTTGTATGGGGTCTCTGTCTGCTCTTTGCCCTCCCAGATTTCATCTACCTATCAGCCAACTACGATC AGCGCCTCAATGCCACCCATTGCCAGTACAACT
**Cxcr4**	NM_00991 1.3	GTTTCAATTCCAGCATATAATGGTGGGTCTCGTCCTGCCCGGCATCGTCATCCTCTCCTGTTACTGC ATCATCATCTCTAAGCTGTCACACTCCAAGGGC
**Cxcr5**	NM_00755 1.2	ACCGTGCCTTTCTCTTAACCAAGCAGAAAGCTGAAACCGATCTACTTTAGGTAGCTGTCTGGTTCC AACCTAACCAGCATTGGGTCAGCCCCATGTTACT
**Cxcr6**	NM_03071 2.4	AGTGAGGAGATATCCACTATGGTTCTTGTTATACAGATGACTCTGGGGTTCTTCCTGCCATTGCTC ACTATGATTCTGTGCTACTCAGGCATTATCAAGA
**Cybb**	NM_00780 7.2	ACAGAAGACTCTGTATGGACGGCCCAACTGGGATAACGAGTTCAAGACCATTGCAAGTGAACACC CTAACACCACAATAGGCGTTTTCCTGTGTGGCCCT
**Cyfip2**	NM_00125 2459.1	TATGAGAATAAGATGTACCTGACGCCCAGTGAAAAGCACATGTTGCTCAAGGTGATGGGCTTTGG TCTCTATCTGATGGACGGAAATGTCAGTAACATTT
**Cyld**	NM_00127 6279.1	CTATGACGATCCGGACATCTCAGCTGGGAAGATCAAGCAGTTCTGTAAGACCTGCAGCACTCAGG TTCACCTTCATCCCAGGAGGTTGAATCATTCTTAT
**Ddx58**	NM_17268 9.3	GTACACATCACATTTGCGGAAATACAACGATGCACTCATCATCAGTGAGGATGCACAGATGACAG ACGCTCTAAATTACCTCAAAGCCTTCTTCCACGAT
**Ddx60**	NM_00108 1215.1	TGCCTTGGTGGAGAAATTGGAGCGGAAATCTGGGAGCATCTCCTTGTTATGATCCGATGTCCCTTT TTGGCCCTTTCAGCTACCATTAGCAACCCTCAGC
**Defb1**	NM_00784 3.3	GGTGTTGGCATTCTCACAAGTCTTGGACGAAGAACAGATCAATACAAATGCCTTCAACATGGAGG ATTCTGTCTCCGCTCCAGCTGCCCATCTAATACCA
**Dll4**	NM_01945 4.2	TGGCAATGTCTCCACGCCGGTATTGGGCACCAACTCCTTCGTCGTCAGGGACAAGAATAGCGGCA GTGGTCGCAACCCTCTGCAGTTGCCCTTCAATTTC
**Dmbt1**	XM_006507 298.1	GTTCAGCTTCTCAATCAAGCAGTCCCACACCTGATGTGTTCTACCCAACTGACCAAACCACAGCAG AGCAGACAACCGTTCCTGATTATACAACAATAGG
**Dock9**	XM_976068 .1	AGCTGATCGAGCCACTGGACTACGAAAATGTCATCGTGCAGAAGAAGACGCAGATCCTAAACGA CTGCCTGCGGGAGATGCTCCTCTTCCCTTATGATGA
**Dpp4**	NM_00115 9543.1	AAGAGGGGATCACTATTTATGTGATGTGGTGTGGGCTACAGAAGAAAGAATTTCACTACAGTGG CTCAGGAGGATTCAGAACTATTCCGTGATGGCTATC

**Dusp4**	NM_176933.4	ACTGGGTGCCGTTCAGATTTTCCTGCCGTGAATCATTTGGTAAGGCCAGATCAGAACCAGATCACTGAAGATGAACTAAAAATGAATCTCCCTCTCGCGG
**Dusp6**	NM_026268.2	TCAGTTCCTCTTGAGCAGCATCGACCAGGCTGCTTTCTTTCTGTGTGTGGCCCCGGGTGTCAAAAGTGTCACCAGCTGTCTGTGTTAGACAAGGTTGCCA
**Ebi3**	NM_015766.2	ATGTACTGGGCTGCTCCGAAGCACTGGATAATTCACTTGACTTCTTCAGACCTCAATTTCCAACCCTGTGGGATGATCTTTCTTCTTCTGCCGGTGCGGG
**Ecsit**	NM_001253897.1	GCCCTTTCAGGAACTCCCTTTGCTCAGGTGTCCCTCCAGGCTCTACGAGGCCTTCACTGTAGTGCAGCTACACACAAGGATGAGCCGTGGTTGGTTCCTC
**Egfr**	NM_207655.2	TGCTACAAACATCAAACACTTCAAATACTGCACTGCCATCAGCGGGGACCTTCACATCCTGCCAGTGGCCTTTAAGGGGGATTCTTTCACGCGCACTCCT
**Egr1**	NM_007913.5	CGGCAGCAGCGCCTTCAATCCTCAAGGGGAGCCGAGCGAACAACCCTATGAGCACCTGACCACAGAGTCCTTTTCTGACATCGCTCTGAATAATGAGAAG
**Egr2**	NM_010118.2	CAGCTCTGTCCCACCTTCTCACGGACGGCCTTCCGAAAACTTAGGCCATTTGAAGGGAGTTGACTGTCACTCCAAGAAATGGGGGAGCAAAAAGAGGGCT
**Egr3**	NM_018781.2	CCAGGATTACCAATCGGCCAAGCCGGCCTTGGACAGCAATCTTTTCCCCATGATTCCTGACTACAACCTGTACCACCATCCCAACGACATGGGCTCCATT
**Elane**	NM_015779.2	AGGTTGGGCACAAACAGACCATCACCCAGTGTGCTACAAGAGCTCAATGTGACAGTGGTGACTAACATGTGCCGCCGTCGTGTGAACGTATGCACTCTGG
**Elk1**	NM_007922.4	AGGGTCTCATCTTTGCCTAAACTTACCAGTTGGGATACACTTGCTGGCCATCAAGTCTCAAGGATCTACCTGGTTCTACCTTGGGATTACAAGCATGTAC
**Emr1**	NM_010130.1	TGATGAGTGCACCCAAGATCCATTACAATGTGGACTGAATTCTGTCTGCACCAATGTACCAGGCTCCTACATCTGTGGCTGCCTCCCTGACTTTCAAATG
**Eng**	NM_001146350.1	ATTTTCCCGGAATGCTGTCACATCTGGAGCTGACTCTTCAGGCATCCAAGCAAAATGGCACGGAGACCCAGGAGGTGTTCCTGGTCCTCGTTTCGAACAA
**Entpd1**	NM_009848.3	CAAACCCAGTCCAAGCCTGCCCTTTGGATAGGAGAGTTTTCTAAAGTAGATAAATATGTGCTAAAGCCAAAGAGTCCTTGCCAAGGAGCTTATGTGCTCG
**Eomes**	NM_010136.2	ACTGAAAAGGTCGTTCAAGGTGCTGGATTGATTCATTTATGGGAAACGAGAAATGTTCAGAAAAAGCAGGCTATGAAGAACGAGTGCCCGGTGCTATTAA
**Ep300**	NM_177821.6	TGGGACCTTTCTGGAGAATCGAGTGAATGACTTTCTGAGGCGACAAAATCACCCTGAATCAGGAGAGGTCACTGTTCGGGTTGTTCATGCTTCTGACAAA
**Epcam**	NM_008532.2	TGAAATTTGTACTCGTAAATACAAGCAGCTGGACACCGGCATTACCGATCGTAAAATTAGACGAACGTCTTATAGGTGCAGGTCCAGTGTGGTACTCAGA
**Epsti1**	NM_029495.2	CTCAGCCCAGGAAGCTAACCTGTGAGAAAGTCCAAGGGTGTCCGGGTAGAGAATGAATGAGGAAGTTCTCTGAACCCAGCGGGTGGTTAAAGAGAAATCG
**Erbb2**	NM_001003817.1	AGATCACAGGTTACCTATACATTTCAGCATGGCCAGAGAGCTTCCAAGACCTCAGTGTCTTCCAGAACCTTCGGGTCATTCGGGGACGGATTCTCCATGA
**Ets1**	NM_001038642.1	GAAAGAGGATGTGAAACCATATCAGGTTAATGGAGCCAACCCTACCTACCCAGAATCCTGTTACACCTCGGATTACTTCATCAGCTACGGTATCGAGCAT
**Ewsr1**	NM_007968.3	ATATGCACAGACCACCCAGGCATATGGGCAACAAAGCTATGGAACCTATGGACAGCCTACTGATGTCAGCTATACTCAGGCTCAGACCACTGCCACCTAC
**F12**	NM_021489.2	CCTCACTGGGAAACATTGCCAGAAAGAGAAATGCTTTGAGCCTCAGCTTCTCAAGTTCTTCCACGAGAATGAGCTATGGTTTAGAACGGGGCCAGGAGGT
**F13a1**	NM_001166391.1	TTTCTTCTTGGCAAAGCCAGAGAAAGGTCTTTCATCTTGCACCTGCAGCCAAGGAACTGCCTGCCAAATTTCACAGATTACCTTGTGAGAAGATGTGGCC
**F2rl1**	NM_007974.4	GCTGGCCATGTACTTCATCTGCTTTGCTCCTAGCAACCTTCTGCTCGTAGTGCATTATTTCCTAATCAAAACCCAGAGGCAGAGCCACGTCTACGCCCTC
**Fadd**	NM_010175.5	TTAAGTGGCAGTATTGAGTTACTACTGTTGCAGGGCTTTTGGGCCACCAAAGAAACTTATCCCTTTGTACAGAGTCCCTTCAACCACGTTGGGGATTAAA
**Fap**	NM_007986.2	CTCTGGCGATATTCATACACAGCGACATACTACATCTACGACCTTCAGAATGGGGAATTTGTAAGAGGATACGAGCTCCCTCGTCCAATTCAGTATCTAT
**Fas**	NM_007987.2	CCACTTGTATTTATATATCGAAAGTACCGGAAAAGAAAGTGCTGGAAAAGGAGACAGGATGACCCTGAATCTAGAACCTCCAGTCGTGAAACCATACCAA

**Fasl**	NM_010177.3	CATTTAACAGGGAACCCCCACTCAAGGTCCATCCCTCTGGAATGGGAAGACACATATGGAACCGCTCTGATCTCTGGAGTGAAGTATAAGAAAGGTGGCC
**Fcer1a**	NM_010184.1	GAGAAATCTGTACTGACCTTGGACCCACCATGGATTAGAATATTTACAGGAGAGAAAGTGACCCTTTCCTGCTATGGGAACAATCACCTTCAAATGAACT
**Fcer1g**	NM_010185.4	CTATAGCCAGCCGTGAGAAAGCAGATGCTGTCTACACGGGCCTGAACACCCGGAGCCAGGAGACATATGAGACTCTGAAGCATGAGAAACCACCCCAGTA
**Fcer2a**	NM_001253737.1	CCCAATCAAAATACACCATCACATCATAGCCCAGTCTAACAGACCGCCCTTTTTCTCTTCATAAAATTACACCTGCAACCAGGCGTAGTGGTGCAGGCCT
**Fcgr1**	NM_010186.5	GAGACAGTTCCACACAATGGTTTATCAACGGAACAGCCGTTCAGATCTCCACGCCTAGTTATAGCATCCCAGAGGCCAGTTTTCAGGACAGTGGCGAATA
**Fcgr2b**	NM_001077189.1	TTGGTTCCCAATGGTTGACTGTACTAATGACTCCCATAACTTACAGCTTCCCAACTCAAGACTCTTCTGCTATCGATCCACACTGCCACTAAAATTAATC
**Fcgr3**	NM_010188.5	TCTGACCTCCACCATCCACCATGGCAGGTGCACACAATAAATTAAAATGTCATGTATATTTTTAAACAAGAGACAGGGGCAGGCTAAGGGTTGATGGCAT
**Fcgr4**	NM_144559.1	GGCGATCCAGGGTCTCCATCCATGTTTCCACCGTGGCATCAAATCACATTCTGCCTGCTGATAGGACTCTTGTTTGCAATAGACACAGTGCTGTATTTCT
**Fez1**	NM_183171.4	GAAGCTCTGAATGGCAACAGCTCTGACATTGAGATTCATGAGAAGGAAGAAGAAGAGTTCAATGAGAAGAGTGAAAATGACTCTGGCATCAATGAGGAGC
**Flt3**	NM_010229.2	CGCCCTACAGGCCGTTGCTTCGCTGGACTTTTCTCTAGATGCTGTCTGCCATTACTCCAAAGTGACTTCTATAAAATCAAACCTCTCCTCGCACAGGTGG
**Flt3l**	NM_013520.2	GGTCTAAGATGCAAACGCTTCTGGAGGACGTCAACACCGAGATACATTTTGTCACCTCATGTACCTTCCAGCCCCTACCAGAATGTCTGCGATTCGTCCA
**Fn1**	NM_010233.1	TCCAGACCCTACCGTGGACCAGGTTGATGATACTTCCATTGTTGTTCGGTGGAGTAGACCCCAGGCACCTATCACAGGGTATAGAATTGTCTATTCACCT
**Fos**	NM_010234.2	AGCTGGTGCATTACAGAGAGGAGAAACACGTCTTCCCTCGAAGGTTCCCGTCGACCTAGGGAGGACCTTACCTGTTCGTGAAACACACCAGGCTGTGGGC
**Foxj1**	NM_008240.3	TAAGCCCGCTAGGGCCTTTCCCTGGTCCATCACCTGCACAAGGGAGGCTACAAACCTTCCAGCCTGACTTGAGGCTAAGAGCATCAGGAAGATGGGGTGT
**Foxp3**	NM_054039.2	TGCCTTCAGACGAGACTTGGAAGACAGTCACATCTCAGCAGCTCCTCTGCCGTTATCCAGCCTGCCTCTGACAAGAACCCAATGCCCAACCCTAGGCCAG
**Fpr2**	NM_008039.2	GTGAGAGATTTATTCATTCCCTGCCTTATAGTCTTGAGAGAGCCCTGAGTGAGGATTCTGGTCAAACCAGTGATTCAAGCACCAGTTCTACTTCACCTCC
**Fut7**	NM_001177367.1	GATGCCTTTGTACACGTGGACGACTTCAGCTCTGCCCGTGAACTGGCTGTCTTCCTCGTCAGCATGAATGAGAGTCGTTATCGTGGCTTCTTTGCTTGGC
**Fyn**	NM_008054.2	TTCTTATCCGCGAGAGCGAAACCACCAAAGGTGCCTACTCACTTTCCATCCGTGATTGGGATGATATGAAAGGGGACCACGTCAAACATTATAAAATCCG
**Gata3**	NM_008091.3	CATGCGTGAGGAGTCTCCAAGTGTGCGAAGAGTTCCTCCGACCCCTTCTACTTGCGTTTTTCGCAGGAGCAGTATCATGAAGCCCGAAAGCGACAGATCT
**Gbp2b**	NM_010259.2	TCAAGAACATGCCTCCACCTCGATCATGCACCATACTTTAAAATCTGAACAGACTAAAGCTCTCTACCCTGTTTCCACTCATCAAGGAAAAAACTTCGGG
**Gbp5**	NM_153564.2	TCGTTCAAGGATGAGGGTGAATTTTTCCAGAAAGAATTGGAGAGCCTACTAAGTGCAAAGCAGGATGAGATTTGTAAGAAGAACGCGGATGCTTCTGCAG
**Gfi1**	NM_010278.2	CAAAGCTCATCATGGTTAGTCCCCTTCACCTTCCTTCCCGGAGCTGCTGGAGGAGATGAACTCCCGTTTCTAAGGTCAACCCAGAGTGGGAACCGCAGCA
**Glycam1**	NM_008134.2	ACTTCAAATGAAGACTCAGCCCACAGATGCCATTCCAGCTGCCCAGTCCACTCCCACCAGCTACACCAGTGAGGAGAGTACTTCCAGTAAGGACCTTTCC
**Gpi1**	NM_008155.4	CGACCAACTCTATTGTGTTTACCAAGCTGACACCCTTCATTCTGGGGGCCTTGATTGCCATGTATGAGCACAAGATCTTTGTTCAGGGCATCATGTGGGA
**Gpr183**	NM_183031.2	CAGCCAGGGTATTAATGCCTCTGCATTACAGCCTGGTCTTCATCATTGGGCTGGTGGGAAACCTGCTGGCCTTGGTTGTCATTGTTCAAAACAGAAAAAA
**Gpr44**	XM_006526695.1	ATCAGAATGGGTGGTTCTGAAGAGTGCAGGAGCGCTGTCTTCTGAGCATCCCATGACACTGACAGTGCAGCTTGAAGGGATGTCTCACAGCAGAAAGAGA

**Gtf3c1**	NM_20723 9.1	ACCAACGGCATGCTAGACCAGCCTGATCATTTTTCTTTCAAGGACCTGGATAGCAGTGACCCTTCA AATGACCTGGTGGCATTTTCTTTGGACAGCCCTG
**Gzma**	NM_01037 0.2	CTGTGCTGGCGCTTTGATTGAAAAGAACTGGGTGTTGACTGCTGCCCACTGTAACGTGGGAAAGA GATCTAAGTTCATTCTTGGGGCTCACTCAATCAAT
**Gzmb**	NM_01354 2.2	TTCTGCCACCATGCTGTGACAACCCAACTGACATCTTCCTATGGAAGTTTGCCCTCTCCACAAAAG AAGTAGAATGTTTGCATTGGAGCTGGGCATGCTC
**Gzmk**	NM_00819 6.1	CATCCCATTCTCACGACTTCAGTCCGGTTCCGCATCGCATGACATCATGCTGATAAAGCTTCGCACT GCTGCAGAACTAAACAAGAATGTCCAACTGCTT
**Gzmm**	NM_00850 4.2	CAGAACCTGAAGCTGGTGCTTGGCCTGCACAACCTCCATGATCTCCAAGATCCTGGCCTCACCTTC TACATCCGGGAAGCCATTAAACACCCTGGCTACA
**H2-Aa**	NM_01037 8.2	TCAGAAATAGCAAGTCAGTCGCAGACGGTGTTTATGAGACCAGCTTCTTCGTCAACCGTGACTATT CCTTCCACAAGCTGTCTTATCTCACCTTCATCCC
**H2-Ab1**	NM_20710 5.2	AAGGCATTTCGTGTACCAGTTCATGGGCGAGTGCTACTTCACCAACGGGACGCAGCGCATACGAT ATGTGACCAGATACATCTACAACCGGGAGGAGTAC
**H2-D1**	NM_01038 0.3	GTGACAGACGATGTGTTCAGGTCTCTCCTGTGACATCCAGAGCCCTCAGTTCTCTTTACACAACAT TGTCTGATGTTCCCTGTGAGCTTGGGTTCAGTGT
**H2-DMa**	NM_01038 6.3	AGCTGTCGATGGGCTGACCTTCCAGGCTTTCTCTTATTTAAACTTCACACCGGAACCCTTTGACCTT TACTCCTGCACTGTGACACACGAGATTGACCGC
**H2-DMb1**	NM_01038 7.2	CTCTATTCAGGTCCCCTTCCTGAAATAAATATTAGTAGTTTGGGGGGGTATTTATAATGAAGTCTG TCCCAGGTGGGGGAGCTGAGGTGAGCTGAAATGA
**H2-DMb2**	NM_01038 8.4	TTTGGGGTGCTGTCTAGATTGGCTGAAATAATTTCAAATATCCTCAACGAACAGGAGAGCCTTATT CATCGCTTGCAAAACGGGCTTCAGGACTGTGCCA
**H2-Ea-ps**	NM_01038 1.2	ATCATGAAGGGTATTAAAAAACGCAATGTTGTAGAACGCCGACAAGGAGCCCTGTGAGATACCT GGAGGCAATGCCTTCAGTTAAAGTTCAGTGAAGAAA
**H2-Eb1**	NM_01038 2.2	AAACATGTCCTGCTTGGCCCACATCCCTCCAGAGACACTGCTCTTCCAGGACCTGGCTCCTCCTGA TTCTCCACCCTGGAGATCTGTGCTCCTGATGGCT
**H2-K1**	NM_00100 1892.2	TCTTCCCTTCCACAGCCAACCTTGCTGGTTCAGCCAAACACTGAGGGACATCTGTAGCCTGTCAGC TCCATGCTACCCTGACCTGCAACTCCTCACTTCC
**H2-M3**	NM_01381 9.2	GAGTCTCCTCTCCATACCTGTGTCTCCTCCTTTTCTCACCTGTACCACAGGGGGATCCAGGACTTCT TCATCCCTTGTGGAACTTTAGGTTTCTATGGGC
**H2-Ob**	NM_01038 9.3	TTTCTATGAGTTAGCATCTGATGCAGACTTGGGGTGAGACTCTAGTCTGATTGTTTTGGAGTCTGG AGGAGGCCATGACCTTTTCTGGAGTTACAGTGTA
**H2-Q1**	NM_01039 0.3	TACTACAACCAGAGCAAGGGCGGCATTCACACCTTCCAGAAGTTGTCTGGCTGTGATCTGGGGTC AGATGGGCGCCTTCAAAGCGGGTACCTGCAGTTCG
**H2-Q10**	NM_01039 1.4	TGAGCCCCTCACCCTGAGATGGGAACCTCCTCCTTCCACTGACTCTATCATGTCACACATTGCTGAT CTGCTGTGGCCATCATTAAAGCTCTGGTGGTAT
**H2-Q2**	NM_01039 2.2	ACCTGGCAGCACTGAAGACCCGAAGCAAGTTGGAGCAGGCTGGTCTTGCAGAGAAGCGCAGGG CCTACCTGGAGGTCGATTGCTTGACGTGGCTCCGCAG
**H2-T23**	NM_01039 8.3	CCTACGATGGCCAGGATTACATCTCCCTGAACGAGGACCTGCGTTCCTGGACCGCGAATGACATA GCCTCACAGATCTCTAAGCACAAGTCAGAGGCAGT
**H60a**	NM_01040 0.2	GGGCAAGGAGTTGGCCAGTTTTCAAAGGTAATTAGGAATATATGGCAAGTACAGCCAGTGATTT GGATGGAGCTGGCAAAGTAAGACACAATTTAATGTG
**Hamp**	NM_03254 1.1	AAGAGAGACACCAACTTCCCCATCTGCATCTTCTGCTGTAAATGCTGTAACAATTCCCAGTGTGGT ATCTGTTGCAAAACATAGCCTAGAGCCACATCCT
**Havcr2**	NM_13425 0.2	TGGAAAATGCTTATGTGTTTGAGGTTGGTAAGAATGCCTATCTGCCCTGCAGTTACACTCTATCTA CACCTGGGGCACTTGTGCCTATGTGCTGGGGCAA
**Hc**	NM_01040 6.1	CTACACACTGAATTTGGTCGCTACTCCTCTTTTCGTGAAGCCCGGGATTCCATTTTCCATCAAGGCA CAGGTTAAAGATTCACTCGAGCAGGCGGTAGGA
**Hck**	NM_01040 7.3	GAGGACTTCACAATCTCTTTCTGACTCTAGTCATCTGCAATCCGCCACTCTCAGGGCCTCCAAGTTG GTATGTCTCATTTGCCTGGAATGACTGAATTCA
**Hcst**	NM_01182 7.3	CATCGGCAGGTTCCTGCTCCGGATGTGGGACTCTGTCTCTGCCACTCCTGGCAGGCCTAGTGGCT GCAGATGCGGTCATGTCACTCCTAATTGTAGGGGT

**Herc6**	NM_025992.2	CCACAACTCATAGAAGGAATTGGTGGCCGTGTTTCACAGATAGAGTGCGCAAGTTATCACACCATTGCATATGTCTACACCACTGGTCAGGTGGTATCTT
**Hif1a**	NM_010431.2	ACCATGATATGTTTACTAAAGGACAAGTCACCACAGGACAGTACAGGATGCTTGCCAAAAGAGGTGGATATGTCTGGGTTGAAACTCAAGCAACTGTCAT
**Hmgb1**	NM_010439.3	GTGGGACTATTAGGATCAAGCAATCTGAACGTCTGTCCTTGAAGGACTGATAGAAAAGTACCTTCTAATCCTTACACGAGGACTCTCCTTTAACCGCCAT
**Hras**	NM_008284.2	CAAATGTTCAGCTTCCCTTGTCTGTGTGTATCTGGGGCTCCTGAGGTATCATCTGGAGCGTCAGTGACACCTTCGGAGCCTTGTTGGTCATGAACTTAAG
**Hsd11b1**	NM_008288.2	CAGGTCCCTGTTTGATGGCAGTTATGAAAAATTACCTCCTCCCGATCCTGGTGCTCTTCCTGGCCTACTACTACTATTCTACAAATGAAGAGTTCAGACC
**Hspb2**	NM_024441.3	CTGTCTACCTCCCGTGGTGATTCCATAAATCCACCACACCCCAGAGGGAGCAGCATCCCTGGGAGATGGCATCGGTGCATGGTCCACAGTGTATGGTTTG
**Icam1**	NM_010493.2	CACATGGGTCGAGGGTTTCTCTACTGGTCAGGATGCTTTTCTCATAAGGGTCGACTTTTTTCACCAGTCACATAAACACTATGTGGACTGGCAGTGGTTC
**Icam2**	NM_010494.1	GAAGTGGAAACAGTTCTTAGTCTCAAACGTCTCCAAAGACACGGTCTTCTTTTGCCATTTCACGTGTTCGGGAAAGCAGCACTCGGAGAGTCTCAACATC
**Icam4**	NM_023892.2	GGATTCTTGGTGGTGAGCCTGAGAAGAGGTGGCCGAGTGATTTATCATGAAAGCCTGGAGCGCTTCACCGGTTCAGATTTGGCTAATGTCACTTTGACCT
**Icos**	NM_017480.1	TTCACAATGGAGGTGTACAGATTTCTTGTAAATACCCTGAGACTGTCCAGCAGTTAAAAATGCGATTGTTCAGAGAGAGAGAAGTCCTCTGCGAACTCAC
**Icosl**	NM_015790.3	CCCCACAGACGCCATTTCAACTTGAGTGGTCTGTATGTCTATTGGCAAATCGAAAACCCAGAAGTTTCGGTGACTTACTACCTGCCTTACAAGTCTCCAG
**Ido1**	NM_008324.1	ACATGGACATTCTGTTCTCATTTCCTGGTGGGGACTGCGACAAGGGCTTCTTCCTCGTCTCTCTATTGGTGGAAATCGCAGCTTCTCCTGCAATCAAAGC
**Ifi27**	NM_026790.2	GGTTCCTGTTTGGAAACTTAACTGGTCCTCATGGCGTTTTCGGGCACAGGGACACTGGTGGCTTCCATTGTCTCCAAGATGACATCTTCAGCAGCCATGG
**Ifi35**	NM_027320.4	GTCTCTCCCTATGTGAGTGGTGAGATCCAGAAAGCCGAGATCAAATTCCAGCAAGCCCCTCATTCAGTGCTGGTGACAAATATTCCTGATGTCATGGATG
**Ifi44**	NM_133871.2	CTGACAGATACCAGTTCGATTCCATGAAACCAATCACATCAAACCATCCGAACTATACCCATGACCCACTGCTGAAGGACAGAATTCACTGTGTGGTTTT
**Ifi44l**	NM_031367.1	ACTGGTAACCTGGCTCCTTTCTAAAGCACTGCCTTTTGGGAAACCCTCAGCTGTAGTTCACTGGGGTGTTTGGGGACCATTTTCCTAGCCCTGACATGTA
**Ifih1**	NM_027835.2	GCTGCTAAAGACGGAAATCGCAAAGATCGCGTCTGTGCAGAACATTTGAGGAAGTACAACGAAGCCCTACAAATCAACGACACGATCCGAATGATTGATG
**Ifit1**	NM_008331.2	AACAGGGCCTTGCAGGCATCACCTTCCTCTGGCTACTTACATTATCAAAAAGGGCTCTGCTACAAGCAACAAATCTCCCAACTGAGGACATCCCGAAACA
**Ifit2**	NM_008332.2	ACAACGAGTAAGGAGTCACTGGAGAGCAATCTGCGACAGCTAAAATGCCATTTCACCTGGAACCTGATAGCAGAAGATGAGTCCTTGGATGAGTTTGAGG
**Ifit3**	NM_010501.1	AATTGTGGTGGATTCTTGGCAGTTGCAGGGATAAAGGAGTGGCTGAATGGTTTTGGGGTTTGGGAGGCAACGCACTTTGGGGCACAGGCAGGCTTTTCCT
**Ifitm1**	NM_001112715.1	CCTCCACCGCCAAGTGCCTGAACATCAGCTCCCTGTTCTTCACCATCCTCACGGCCATCGTCGTCATCGTTGTCTCTGTGCCATTAGATGATGTGAGATGTC
**Ifitm2**	NM_030694.1	GCTTCCTCCAAGCTATGAGACAATCAAAGAGGAGTACGGGGTGACTGAGCTGGGGGAACCCAGCAACTCAGCTGTTGTGAGGACCACCGTGATCAACATG
**Ifna1**	NM_010502.2	CTGGCGGTGCTGAGCTACTGGCCAACCTGCTCTCTAGGATGTGACCTTCCTCAGACTCATAACCTCAGGAACAAGAGAGCCTTGACACTCCTGGTACAAA
**Ifna2**	NM_010503.2	TAACCTCAGGAACAAGAGGGCCTTGAAGGTCCTGGCACAGATGAGGAGGCTCCCCTTTCTCTCCTGCCTGAAGGACAGGCAGGACTTTGGATTCCCCCTG
**Ifna4**	NM_010504.2	CAGAAGGCTCAAGCCATCCTTGTGCTAAGAGATCTTACCCAGCAGATTTTGAACCTCTTCACATCAAAAGACTTGTCTGCTACTTGGAATGCAACTCTAC
**Ifnar1**	NM_010508.1	TGGGAAAACACTTCCAATACTAAGATAAGCATGGAGAAGGATGGCCCAGAGTTCACCCTCAAGAACCTGCAGCCGCTGACTGTGTACTGTGTCCAGGCCA

**Ifnar2**	NM_001110498.1	TCCTCTTGAGCCGCCAGAATTTGAGATCGTTGGCTTTACAGACCACATAAACGTGACGATGGAATTTCCACCTGTCACTTCCAAAATAATCCAGGAAAAG
**Ifnb1**	NM_010510.1	GATGAACTCCACCAGCAGACAGTGTTTCTGAAGACAGTACTAGAGGAAAAGCAAGAGGAAAGATTGACGTGGGAGATGTCCTCAACTGCTCTCCACTTGA
**Ifng**	NM_008337.1	CTAGCTCTGAGACAATGAACGCTACACACTGCATCTTGGCTTTGCAGCTCTTCCTCATGGCTGTTTCTGGCTGTTACTGCCACGGCACAGTCATTGAAAG
**Ifngr1**	NM_010511.2	AAGCATAATGTTACCTAAGTCCTTGCTCTCTGTGGTAAAAAGTGCCACGTTAGAGACAAAACCTGAATCGAAGTATTCACTTGTCACACCGCACCAGCCA
**Ifnl2**	NM_001024673.2	GCCACCAGGCTCCCAGTGGAAGCAAAGGATTGCCACATTGCTCAGTTCAAGTCTCTGTCCCCAAAAGAGCTGCAGGCCTTCAAAAAGGCCAAGGATGCCA
**Igf1r**	NM_010513.2	CGTATGAGAACTTCATGCATCTGATCATTGCTCTGCCGGTTGCCATCCTGCTGATCGTTGGGGGCTGGTTATCATGCTGTATGTCTTCCATAGAAAGAG
**Igf2r**	NM_010515.1	TGCATCTGCTTGCCAGATGAAATATGAAAATCATGAGGGCTCCTTGGCAGAGACTGTATCCATCAGTAACTTGGGAGTTGCAAAGATAGGCCCTGTGGTG
**Igll1**	NM_001190325.1	GTGGAGAAGAGTGTGTCACCTGCTGAGTGTTCTTAGACCACAATCCTCCCTGAAGCCTCAGGGGCCTGGATCTGAAGTGCCAGAAAAAGTTGTTTTTTGT
**Ikbkb**	NM_010546.2	CTTACCCTGCTGAGTGACATCGCATCGGCTCTTAGATACCTTCACGAAAACAGAATCATCCATCGAGACCTGAAGCCAGAAAACATCGTTCTGCAGCAAG
**Ikbke**	NM_019777.3	TGGGAGCCTGCTGAGCGTGCTGGAAGACCCTGAGAACACGTTCGGGCTTTCTGAAGAGGAGTTCCTAGTGGTGCTGCGCTGTGTGGTGGCTGGCATGAAC
**Ikbkg**	NM_178590.2	TGCCAACAGATGGCTGAGGACAAGGCCTCTGTGAAAGCTCAGGTGACATCATTGCTCGGAGAACTCCAGGAGAGCCAGAGCCGTTTGGAGGCTGCCACCA
**Ikzf1**	NM_001025597.1	GATTCCAGTGCCCTCAAGTTGTGTTTTAAGTAGCTATCAGAGGCAAGAGGGTTCCTAAGAGCAGGTTGACCTGTTGGCGACAGATGGCAATCACCATTTC
**Ikzf2**	NM_011770.4	AATTATGGAACTGTGATCTAAAAACGGTTTGACAGTGGAGCACCAGAGAGGATGTCCACCACTCACTTGCCCTTCGGTACGACTAAGCTCTGAGTTTGCG
**Il10**	NM_010548.1	GGGCCCTTTGCTATGGTGTCCTTTCAATTGCTCTCATCCCTGAGTTCAGAGCTCCTAAGAGAGTTGTGAAGAAACTCATGGGTCTTGGGAAGAGAAACCA
**Il10ra**	NM_008348.2	TGTTGTCGCGTTTGCTCCCATTCCTCGTCACGATCTCCAGCCTGAGCCTAGAATTCATTGCATACGGGACAGAACTGCCAAGCCCTTCCTATGTGTGGTT
**Il11**	NM_008350.2	GCGCTGGGACATTGGGATCTTTGCAGCTTCCTGGTGTGCTGACAAGGCTTCGAGTAGACTTGATGTCCTACCTCCGGCATGTACAATGGCTGCGCCGTGC
**Il11ra1**	NM_010549.3	GGGTCCCACAACTCAGTGGAGCGGGAGACAGTTCTTAGCCTGTAGGAGGAAGTCTTGGAGGCCATGGGCACTCAGTCACTGTGATTATCAAGATGAGCAG
**Il12a**	NM_008351.1	TCATGAAGACATCACACGGGACCAAACCAGCACATTGAAGACCTGTTTACCACTGGAACTACACAAGAACGAGAGTTGCCTGGCTACTAGAGAGACTTCT
**Il12b**	NM_008352.1	TCGTAGAGAAGACATCTACCGAAGTCCAATGCAAAGGCGGGAATGTCTGCGTGCAAGCTCAGGATCGCTATTACAATTCCTCGTGCAGCAAGTGGGCATG
**Il12rb1**	NM_008353.2	TGAACCTCTCCGGGGCCACCTATGACCTGAATGTGCTCGCCAAAACTCGTTTCGGTCGCAGCACCATCCAGAAGTGGCACCTTCCTGCCCAAGAGCTCAC
**Il12rb2**	NM_008354.3	CTTCTGCACCCACTCACATTAACATAGTGGACCTATGTGGCACTGGGTTGCTGGCTCCTCACCAGGTCTCTGCAAAGTCGGAGAACATGGACAACATTCT
**Il13**	NM_008355.2	AGCTACACAAAGCAACTGTTTCGCCACGGCCCCTTCTAATGAGGAGAGACCATCCCTGGGCATCTCAGCTGTGGACTCATTTTCCTTTCTCACATCAGAC
**Il13ra1**	NM_133990.4	CTCAAACCGACCGACATAATATTTTAGAGGTTGAAGAGGACAAATGCCAGAATTCCGAATCTGATAGAAACATGGAGGGTACAAGTTGTTTCCAACTCCC
**Il13ra2**	NM_008356.3	GACCCATTCCACCAAGGTGTTACACTTATGAAATTGTGATCCGAGAAGACGATATTTCCTGGGAGTCTGCCACAGACAAAAACGATATGAAGTTGAAGAG
**Il15**	NM_008357.2	CTTGCAAACAGCACTCTGTCTTCTAACAAGAATGTAGCAGAATCTGGCTGCAAGGAATGTGAGGAGCTGGAGGAGAAAACCTTCACAGAGTTTTTGCAAA
**Il15ra**	NM_008358.2	CTTTCCTGGCCTGGTACATCAAATCAAGGCAGCCTTCTCAGCCGTGCCGTGTTGAGGTGGAAACCATGGAAACAGTACCAATGACTGTGAGGGCCAGCAG

**Il16**	NM_01055 1.3	TGCGAGACAAAGCTGTTGGATGAAAAGGCCAGTAAGCTTTACTCCATCAGCAGCCAGCTATCATC TGCTGTCATGAAATCCCTGCTGTGCCTTCCATCTT
**Il17a**	NM_01055 2.3	ACCTCAAAGTCTTTAACTCCCTTGGCGCAAAAGTGAGCTCCAGAAGGCCCTCAGACTACCTCAACC GTTCCACGTCACCCTGGACTCTCCACCGCAATGA
**Il17b**	NM_01950 8.1	CCATGGGGCTACAGCATCAACCACGACCCCAGCCGCATCCCTGCGGACTTGCCCGAGGCGCGGTG CCTATGTTTGGGTTGCGTGAATCCCTTCACCATGC
**Il17f**	NM_14585 6.2	TGTGAAGCCCGATCTCCAAGTCTTTATGCTTTCTAGGACTCTCAGTAAGGTGTGCATGGCATTCTT GCAGCTCTGCAGTAGATATAGCTTGAACTTTCTG
**Il17ra**	NM_00835 9.1	CCCAAAAACCTGACCCCGTCTTCCCCAAAAAACATCTATATCAATCTTAGTGTTTCCTCTACCCAGC ACGGAGAATTAGTCCCTGTGTTGCATGTTGAGT
**Il17rb**	NM_01958 3.3	GCCAGGGAGAACTCTCAGGATCTGTTCCCTCTTGCCTTTAACCTCTTTTGTAGTGATTTCAGCAGCC AGACGCATCTGCACAAATACCTGGTGGTCTATC
**Il18**	NM_00836 0.1	ACTTTGGCCGACTTCACTGTACAACCGCAGTAATACGGAATATAAATGACCAAGTTCTCTTCGTTG ACAAAAGACAGCCTGTGTTCGAGGATATGACTGA
**Il18r1**	NM_00116 1842.1	AGATGGTTCAAAGGCAGTGCTTCACATGAGTATAGAGAGCTGAACAACAGAAGCTCGCCCAGAG TCACTTTTCATGATCACACCTTGGAATTCTGGCCAG
**Il18rap**	NM_01055 3.2	CTTGAAGTCGGTCCACGCCAGTTCCAGGTTCTGGACCCAAATTCGTTACCACATGCCTGTGAAGAA CTCCAACAGGTTTATGTTCAACGGGCTCAGAATT
**Il19**	NM_00100 9940.1	TCTAGGAGAACTGAACATACTTTTAGCCTGGATTGACAGGAATCATCTGGAAACTCCTGCAGCCT GACACGAAACGCCTCGTCTGATTATCTAAATAACG
**Il1a**	NM_01055 4.4	ACCTCTGAAACGTCAAAGATGTCCAACTTCACCTTCAAGGAGAGCCGGGTGACAGTATCAGCAAC GTCAAGCAACGGGAAGATTCTGAAGAAGAGACGGC
**Il1b**	NM_00836 1.3	GTTGATTCAAGGGGACATTAGGCAGCACTCTCTAGAACAGAACCTAGCTGTCAACGTGTGGGGG ATGAATTGGTCATAGCCCGCACTGAGGTCTTTCATT
**Il1r1**	NM_00112 3382.1	CTTCTTCGGAGTAAAAGATAAACTGTTGGTGAGGAATGTGGCTGAAGAGCACAGAGGGGACTAT ATATGCCGTATGTCCTATACGTTCCGGGGGAAGCAA
**Il1r2**	NM_01055 5.4	AGAAACGCATCCCACTGTGAGCAAATGTCTGTGGAACTCAAGGTCTTTAAGAATACTGAAGCATC TCTGCCTCATGTCTCCTACTTGCAAATCTCAGCTC
**Il1rap**	NM_00836 4.2	ACAGTCAAACTGCAGCTGTTCCTCGTGACTGATTAAGCGACATACTGCCTTCCTGCTACTAAACCC CATTCGCTCTCATCTCAGGTAATGGAGATCATAT
**Il1rapl2**	NM_03068 8.1	TGGATGGCTGCATTGATTGGTCGGTGGATCTCAAGACATACATGGCTTTGGCAGGTGAACCTGTC CGAGTGAAATGTGCCCTTTTCTACAGTTATATCCG
**Il1rl1**	NM_00102 5602.2	ATAGGAAAACCAGCAAGTATTGCCTGTTCAGCTTGCTTTGGCAAAGGCTCTCACTTCTTGGCTGAT GTCCTGTGGCAGATTAACAAAACAGTAGTTGGAA
**Il1rl2**	NM_13319 3.3	AGGAAGTTGAGTATGGAAGAAGGATCCCTAACATCACGTATCCAAAGAACAACTCCATTGAAGTT CCACTTGGCTCCACCCTCATCGTGAACTGCAATAT
**Il1rn**	NM_03116 7.5	CAACCAGCTCATTGCTGGGTACTTACAAGGACCAAATATCAAACTAGAAGAAAAGATAGACATGG TGCCTATTGACCTTCATAGTGTGTTCTTGGGCATC
**Il2**	NM_00836 6.3	TCTTCAGTGCCTAGAAGATGAACTTGGACCTCTGCGGCATGTTCTGGATTTGACTCAAAGCAAAA GCTTTCAATTGGAAGATGCTGAGAATTTCATCAGC
**Il21**	NM_02178 2.2	ATGGCCTGGGGGATGGTTTTGATCTAAGGAAAAAGGTGTCTGTACCTCACAGTGCCTTTAAAACA AGCAGAGATCCCGTGTACCGCCCTAAGATAGCACA
**Il21r**	NM_02188 7.1	TATGGTACACGTGCCATATGCGCTTGTCTCAATTCCTGTCCGATGAAGTTTTCATTGTCAATGTGAC GGACCAGTCTGGCAACAACTCCCAAGAGTGTGG
**Il22**	NM_01697 1.1	AGAAGAATGTCAGAAGGCTGAAGGAGACAGTGAAAAAGCTTGGAGAGAGTGGAGAGATCAAG GCGATTGGGGAACTGGACCTGCTGTTTATGTCTCTGAG
**Il22ra1**	NM_17825 7.1	ACTCCTTCTCGGGCGCCGTGCTCTTTTCCATGGGTTTCCTCGTCGGCTTGCTCTGTTATCTGGGCTA CAAATACATCACCAAGCCACCTGTACCTCCTAA
**Il22ra2**	NM_17825 8.5	TAAGCATTGCCTTCTAGGTCTCCTCATCATACTCTTGAGCAGTGCAACAGAAATACAACCAGCTCG TGTATCTCTGACGCCCCAGAAGGTCCGATTTCAG
**Il23a**	NM_03125 2.1	CAAGGACAACAGCCAGTTCTGCTTGCAAAGGATCCGCCAAGGTCTGGCTTTTTATAAGCACCTGCT TGACTCTGACATCTTCAAAGGGGAGCCTGCTCTA

**Il23r**	NM_14454<br>8.1	AAGTATTTGGTATGGGTCCAAGCTGTCAATTCCCTAGGCATGGAGAACTCACAACAACTACACGTC<br>CATCTGGATGATATAGTGATACCTTCTGCGTCCA
**Il24**	NM_05309<br>5.2	CATAGTCATCATGTCACAACTACAGCCCAGTAAGGACAATTCCATGCTTCCCATTAGTGAGAGTGC<br>ACACCAGCGGTTTTTGCTGTTCCGCAGAGCATTC
**Il25**	NM_08072<br>9.2	TTGGGGAGAAACTCTGACTTTTGCACTTTTTGGAAGCACTTTTGGGAAGGAGCAGGTTCCGCTTGT<br>GCTGCTAGAGGATGCTGTTGTGGCATTTCTACTC
**Il27**	NM_14563<br>6.1	GCCATGAGGCTGGATCTCCGGGACCTGCACAGGCACCTCCGCTTTCAGGTGCTGGCTGCAGGATT<br>CAAATGTTCAAAGGAGGAGGAGGACAAGGAGGAAG
**Il2ra**	NM_00836<br>7.2	AAGGAATTGGTCTATATGCGTTGCTTAGGAAACTCCTGGAGCAGCAACTGCCAGTGCACCAGCAA<br>CTCCCATGACAAATCGAGAAAGCAAGTTACAGCTC
**Il2rb**	NM_00836<br>8.3	TTATTTTATGTGTTCTTGGGCGATGCTGGTCTATGTAAGGGGTGGGGGCGCGGGGTTGATTGAAG<br>TGAGGGCTCCTTCTATGTTGCTTTGGGGACCTTGT
**Il2rg**	NM_01356<br>3.3	ATCCAATGCTCACTGCCTTCCCTTGGGGCTAAGTTTCGATTTCCTGTCCCATGTAACTGCTTTTCTGT<br>TCCATATGCCCTACTTGAGAGTGTCCCTTGCC
**Il3**	NM_01055<br>6.4	TGCAGCTCTATTGTCAAGGAGATTATAGGGAAGCTCCCAGAACCTGAACTCAAAACTGATGATGA<br>AGGACCCTCTCTGAGGAATAAGAGCTTTCGGAGAG
**Il34**	NM_00113<br>5100.1	TGGTGAGTCTCAATGCCACTGAGTCTGTGATGGATGTACTTCTCGAGGGCCACCCGTCCTGGAAG<br>TATCTACAGGAGGTTCAGACATTGCTGGAGAACGT
**Il3ra**	NM_00836<br>9.1	GGGTGACGTGCACTACCGGATGTTCTGGCGTGATGTGCGGCTCGGTCCTGCCCACAACCGCGAGT<br>GTCCACACTACCACAGCCTTGATGTCAACACCGCT
**Il4**	NM_02128<br>3.1	TGCTTGAAGAAGAACTCTAGTGTTCTCATGGAGCTGCAGAGACTCTTTCGGGCTTTTCGATGCCTG<br>GATTCATCGATAAGCTGCACCATGAATGAGTCCA
**Il4ra**	NM_01055<br>7.1	CCCACAGCAGTGCTGACGTTCCTAAGTCCTGGGCTTTCCTAGCTGATGTTGTCCTACCTACTCAGTC<br>CCATTTTGTCCACCGAATAGACCTGTCACTCAA
**Il5**	NM_01055<br>8.1	CAATGAGACGATGAGGCTTCCTGTCCCTACTCATAAAAATCACCAGCTATGCATTGGAGAAATCTT<br>TCAGGGGCTAGACATACTGAAGAATCAAACTGTC
**Il5ra**	NM_00837<br>0.2	ACATGTCCCTGTGTTCCATAGGCCTGTTCAAGGAAATGTCTAAGCCAAAGTAAGTTTAAGTCACCG<br>TGCCTGGGGTGAAAAAGATGGTTCAGATGACGAA
**Il6**	NM_03116<br>8.1	CTCTCTGCAAGAGACTTCCATCCAGTTGCCTTCTTGGGACTGATGCTGGTGACAACCACGGCCTTC<br>CCTACTTCACAAGTCCGGAGAGGAGACTTCACAG
**Il6ra**	NM_01055<br>9.2	CACCTCACTGCCACCTTGGCCTTCCTTGCTTTACGTTTGACTGAGTGGCCTCAGATGCTTTCCCCTG<br>GGGCTTTGAGGAATCCAGTGATGTTAGTGGTCA
**Il6st**	NM_01056<br>0.2	AGAAGCAAACAACAAGAAGCCTTGTCCAGATGACCTGAAGTCCGTGGACCTGTTCAAGAAGGAG<br>AAAGTGAGTACAGAAGGGCACAGCAGTGGCATCGGG
**Il7**	NM_00837<br>1.2	AAACATTCATTGGTGAACCACTGGGGGAGTGGAACTGTCCTGTTTTAGACTGGAGATACTGGAGG<br>GCTCACGGTGATGGATAATGCTCTTGAAAACAAGA
**Il7r**	NM_00837<br>2.3	GTTTCCTGGACTGCCAGATTCATGAGGTGAAAGGCGTTGAAGCCAGGGACGAGGTGGAAAGTTT<br>TCTGCCCAATGATCTTCCTGCACAGCCAGAGGAGTT
**Il9**	NM_00837<br>3.1	TCTGTTTTGCTCTTCAGTTCTGTGCTGGGCCAGAGATGCAGCACCACATGGGGCATCAGAGACAC<br>CAATTACCTTATTGAAAATCTGAAGGATGATCCAC
**Ilf3**	NM_01056<br>1.2	GAGGTTGAGGTGGACGGACAGAAGTTTCAAGGTGCTGGTTCAAACAAAAAGGTGGCAAAGGCTT<br>ATGCTGCACTTGCCGCATTAGAAAAACTTTTCCCTG
**Inpp5d**	NM_00111<br>0192.1	GCCGTCCTGGTGCGACCGAGTCCTCTGGAAGTCTTACCCGCTGGTGCATGTGGTCTGTCAGTCCTA<br>TGGCAGTACCAGTGACATCATGACGAGTGACCAC
**Irak1**	NM_00836<br>3.2	AGGTGGAACAGCTATCAAGGTTTCGTCACCCAAATATCGTAGACTTTGCTGGCTACTGTGCAGAG<br>AGTGGCTTATACTGCCTTGTTTATGGCTTCTTGCC
**Irak2**	NM_00111<br>3553.1	AGAAAGGCTTTTGGGTTTGCCCGGAGACAGGCTTTTCTGGAGTGAAGCAGATGTCGTCCAAGCAA<br>CTGAGGACTTTGACCAAAGCCACCGAATCAGCGAG
**Irak3**	NM_02867<br>9.3	AGCCCACCATCATTACCACCGGCACTTAATGCTAGTCTTTCTGCTAGGGATACTGACAGTCTATTTG<br>CTTCCCATGGTCATAGGGAAGTTGCTCAAATGC
**Irak4**	NM_02992<br>6.5	TTGATCCTCAAGAAGGGTGGAAGAAATTAGCAGTAGCTATCAAAAAGCCGTCCGGCGACGACAG<br>ATACAATCAGTTCCATATAAGGAGATTCGAAGCCTT

**Irf1**	NM_008390.1	TGTTCCGGAGCTGGGCCATTCACACAGGCCGATACAAAGCAGGAGAAAAAGAGCCAGATCCCAAGACATGGAAGGCAAACTTCCGTTGTGCCATGAACTC
**Irf2**	NM_008391.2	GCCATGAATTCCCTGCCCGACATTGAGGAAGTGAAGGACAGAAGCATAAAGAAAGGAAACAACGCCTTCAGAGTCTACCGGATGCTGCCCTTATCCGAAC
**Irf3**	NM_016849.4	GAAAGAAGTGTTGCGGTTAGCTGCTGACAATAGCAAGGACCCTTATGACCCTCATAAAGTGTATGAGTTTGTGACTCCAGGGGCGCGGGACTTCGTACAT
**Irf4**	NM_013674.1	AAGGGACTTGGTTCTTAGGTCCAGTCTAGGCTATCATGTCCTGATCCTGCACTCTCTCTGAGTAGACCCTACATCCTGTTTGCTCCCTCTTCTTGCCCTT
**Irf5**	NM_012057.3	CCTTTAGTACCACGGAGTCCAATTACCCCAGGGTAGCCAACTTTTAAGGTTGCCCCAATTCTTCTAGCAAAACCTGCCAAGGAGTGTTGTGGGGGCTTTC
**Irf7**	NM_016850.2	CGCTGTGCACTCCACAGCACAGGGCGTTTTATCTTGCGCCAAGACAATTCAGGGGATCCAGTTGATCCGCATAAGGTGTACGAACTTAGCCGGGAGCTTG
**Irf8**	NM_008320.3	GCAGGGAGAGTCATCATCTGCTTTTGTCTGAGAGAAGGAGAGCTTCTCCGTTTGTTCAACTTTGTAACAAGCTGGGTTACATGCTCCACGCAGCTAGAGA
**Irgm2**	NM_019440.2	TCCAGAGACTCCTTCATAGACACGCCCAAAGGTCTCTTTGCATAGTGATTCTAGATCCTGCCAACCTGCCAACCAATATTAACTACACAGGCACTCAGGG
**Isg15**	NM_015783.1	GGTCTTTCTGACGCAGACTGTAGACACGCTTAAGAAGAAGGTGTCCAGCGGAACAAGTCACGAAGACCAGTTCTGGCTGAGCTTCGAGGGAAGGCCCATG
**Isg20**	NM_020583.5	AAGCTGGTGGTGGGCCATGACCTGAAGCACGACTTCAATGCCCTGAAGGAGGATATGAGCAAGTACACCATCTATGACACGTCCACAGACAGGCTGCTGT
**Itch**	NM_008395.2	GACAAAGAGCCAACAGAGACGATGGGAGATTTGTCAGTTTGTCTTGATGGGCTGCAAGTAGAAGCTGAGGTTGTTACTAACGGTGAAACGTCATGCTCCG
**Itga1**	NM_001033228.3	ATTGAAGTCTAAAGAAGACTCAGTTTACGAGGCTGATCTGCAGTACCGTGTCACCCTTGATTCGCTGAGGCAGATATCACGGAGCTTTTTTTCTGGAACT
**Itga2**	NM_008396.2	ATTTACAATGGTCACCAGGGTACCATTCGCACCAAGTACTCCCAGAAAATCCTTGGGTCCAATGGAGCCTTTAGGAGACATCTCCAGTTCTTTGGAAGGT
**Itga2b**	NM_010575.2	CCCGTTGCTCTTCGACCTCAGGGATGAGACACGAAACCTAGGCTTCCAAATTTTCCAAACCTTCAAGACCGGGCAAGGACTTGGGGCGTCGGTCGTCAGC
**Itga4**	NM_010576.3	AATTTGTTGAGACCACTGGTCACTTGGACAGTCCCTTACTTCAACAGGGTGGAGCTTTGATCTTCAGGCTACTGGCCTTAGTCATCTGACAAGACTTAGA
**Itga5**	NM_010577.3	CAGGCACCAGTCTCTGGGGTGGCCTTCGGTTCACTGTTCCTCATCTTCAAGACACAAAGAAAACCATCCAGTTTGACTTTCAGATCCTCAGCAAGAACCT
**Itga6**	NM_008397.3	CAGTGGGGCTGTAGTTTTGCTAAAAAGAGACATGAAGTCCGCGCATCTGCTCCCTGAGTATATATTTGACGGAGAAGGCCTGGCTTCCTCGTTTGGCTAT
**Itgae**	NM_008399.1	CCAGACTGAGTTTGATCTTCAAGAGAGCCGGGACATTAACGCCTCCCTTGCCAAAGTTCAGAGCATCGTTCAAGTGAAGGAAGTCACTAAGACCGCCTCA
**Itgal**	NM_008400.2	TAACCCGCTACATCATCGGGATTGGCAAGCATTTTGTGAGCGTACAAAAGCAAAAGACGCTCCACATATTTGCCTCAGAACCTGTAGAGGAATTTGTGAA
**Itgam**	NM_008401.2	ATCTGGACACTGAACATCCCATGACCTTCCAAGAGAATGCAAAAGGCTTTGGACAGAGTGTGGTCCAGCTTGGCGGAACCAGTGTGGTTGTTGCAGCCCC
**Itgax**	NM_021334.2	GGGCCTGTCCCTTGCTGCTGCCACCAACCCTTCCTGGCTGTTGGCTTGTGGTCCTACTGTGCACCACACATGCAGAGAGAATATATACTTGACAGGGCTC
**Itgb1**	NM_010578.1	CTGTGATAGGTCTAATGGCTTAATTTGTGGAGGCAATGGCGTGTGCAGGTGTCGTGTTTGTGAATGCTATCCCAATTACACTGGCAGTGCATGTGACTGT
**Itgb2**	NM_008404.4	GCTCGGTTTCTTTCCGCCATTATATCAAGTCTGCCAGGGTTTCCAGGGACTTGTCTTCCGACCTGCACAATCTTGCCGCAGAGCCCTAAGAATTGTCCCG
**Itgb3**	NM_016780.2	TGGGAATGTATCTCTCTGTGTGTACTTATGTGTGTGACGAGTGTGGGGAAATGTGTAATTTAAAACTTGTGATGTGTCTCACAGGCAGAGCTCCACAGCC
**Itgb4**	NM_001005608.2	TGCCACCCTTGGTCGCCGTCTGGTAAACATCACCATTATCAAGGAACAAGCTAGTGGGGTAGTGTCCTTCGAGCAGCCTGAATACTCGGTGAGTCGTGGA
**Itk**	NM_010583.3	GCGCTGGGTGCTGACCCTTAAAGAAGAAACGAGGAATAACAACAGCCTGGTATCCAAGTATCACCCTAATTTCTGGATGGATGGGCGGTGGAGGTGCTGC

**Jak1**	NM_146145.2	CATAGCAAAGGACTGTGCCGCTGGCATATTGATCTCAGATAAAAACTTGTGGACTTGGCTGACACTCTCCCTTGCCCTGAAATCTCAATGTCTATTCAGT
**Jak2**	NM_008413.2	GATCCAAGACTATCACATTTTAACCCGGAAGCGAATCAGGTACAGATTTCGCAGATTCATTCAGCAATTCAGTCAATGTAAAGCCACTGCCAGGAACCTA
**Jak3**	NM_010589.5	TTCTCTTTTGGGGACTACTTGGCTGAGGATTTATGTGTGCGAGCTGCCAAGGCCTGTGGCATCCTGCCTGTTTATCATTCGCTTTTCGCTCTGGCCACTG
**Jam3**	NM_023277.4	GCAGTGAATCTCAAATCCAGCAACCGAAACCCAGTGGTACATGAATTTGAAAGTGTGGAATTGTCTTGCATCATTACGGACTCACAGACAAGTGACCCTA
**Jun**	NM_010591.2	CGCGACCAGAACGATGGACTTTTCGTTAACATTGACCAAGAACTGCATGGACCTAACATTCGATCTCATTCAGTATTAAAGGGGGGTGGGAGGGGTTACA
**Kdr**	NM_010612.2	TTAAATTGTACAGCGAGAACAGAGCTCAATGTGGGGCTTGATTTCACCTGGCACTCTCCACCTTCAAAGTCTCATCATAAGAAGATTGTAAACCGGGATG
**Kit**	NM_001122733.1	CAGTTTCCCTGCATGTCGCTCACTGTCTAGAATTTACTCAAAGCCGCCACAGAGGCTTAGCGGAGTGAAGTGCCGAAGGACCTCTTTATTTGGAGTCCTC
**Klra1**	NM_016659.3	ACAAAGTCCTCACTTCTCTCTGTGTTAAATCCTTCATTGCTGAAACAGCTTCTTTCCCAGGAATTCTCATCACAAACACATGTACAAAACTCATAGAAGA
**Klra15**	NM_013793.2	ATCAATCATTCATCTGTATTTGTGGGAAGAGATTGGACAAATTCCCTCATTGACTCTCTGACAAGTGTTAAAGGTAAAAAAATGAATTTTTCTGACTCTC
**Klra17**	NM_133203.4	CCCATAACTGCAGGATCTATTATCTTCACCAGAATCTCTTCAGGGTAGAGACACAGGCACCATTTTAACAGAGAACACACTCTACATACACCCATGATGA
**Klra2**	NM_001170851.1	CCGTCAAGAGTACCAGGTCATGAAAAATGACAGCTCCTTAATGGAAGAAATGTTAAGAAATAAGTCTTCAGAGTGTAAGGCCCTCAATGATAGCCTGCAC
**Klra20**	NM_053150.2	CCCTGGAAGCTCATTGTGATAGCTTGTGGAATCCTCTGTTTTCTTCTCCTGGTAACTGTTGTATTGTTGGCAATAAAAATCAACAAAAAACATGAACTGC
**Klra21**	NM_053151.1	AGGCATCCATCCTTTCTACAGGCATCACTTCAGGGTGGAGACACAGAACATACCTCTACTTATCCCATAAGATGAGTGAGCAGGAGGTCACTTTCCCAAC
**Klra27**	XM_916590.3	ATGAAGATGAACTGTCTTTTATCTCCATTGAGTGCCAAGTAAACACCTCTTTTTTAGGATTTGCTTTTTGGAACCTGGAAGTGAGGCTGCCTGAGATGAG
**Klra3**	NM_010648.2	GAGGGGCAGAAAATCATGAGGTTGAGTATCACCCGGTGGAAATTTAGTTCCGACTTTCAATTTTGAAACTCGTAGGAGATCTAAACCAGAAAACGCCAAC
**Klra4**	NM_010649.3	GAAGACACTTTCTCAGCTGTGAGATTCCATAAGTCTTCAGGGTTGCAGAACGAGATGAGGCTCAAGGAGACACGGAAGCCTGAAAAAGCTCGCCTCAGAG
**Klra5**	NM_008463.2	GTGAGCCATGAGGAGATTCAAGGGCCCGGAGAAGCTGGCTACAGAAAGTGTTCAGTACCCTGGCAGCTCACTGTGAGATCGCTTGGAATCTTCTGTTTCC
**Klra6**	NM_008464.2	ACTTTCCACCAAAGTTAAAGAACATAGACTGCTGTGAATAGAGACAAAGCTGCAAGAGCATTGGGACTGTTCTCTGGTATCTAACTTGCAGAACAGAGGC
**Klra7**	NM_001110323.1	GTCCCCTGGAAGCTCATTGTGATAGCTTGTGGAATCCTCTGTTTCCTTCTCCTGGTAACTGTTGCATTGTTGGCAATAACGATTTTTCAGCATAGTCAAC
**Klrb1**	NM_001099918.1	AGACCTTGGGCTGACGGTCTGGTTGACTGTAATTTGAAAGGAGCCACTTTGCTGCTCATTCAAGATGAAGAAGAATTGAGATTATTGCAGAACTTCTCAA
**Klrb1c**	NM_008527.2	GGTGTGGGAACAGAGCAGAGCATTCACAGAGGACTTTCTTTTGCTTATTTCACCTCAAAGGCCTTGGAGTTTGTCCCTTCTCACCACCAGTTAAGTGTTG
**Klrc1**	NM_001136068.1	ACCAAGGGGTCCTCGCAGCTCCATTTCAGTCATCGAGCAGGAAATCATCTATTCGGACTTCAGCTTTCAAAATCCTTCTCAGGAGCATCCCTGGATCTGC
**Klrc2**	NM_010653.4	TTGTTTCTCCATATGCTGAACTGAAGAAGCAGATCCACCCCTTCTTGGTTAGAACCCAGAAAGGCTCACACGCCTTGCTCTCCAAAGCACAGCCTTGTCC
**Klrd1**	NM_010654.2	AGTTATCAGGCCAGAGCACTGCATTGTTTACAGTCCAAGCAAAAGCGTTTCTGCTGAATCCTGTGAAAATAAAAACCGTTACATCTGTAAGAAACTGCCT
**Klrg1**	NM_016970.1	TGGATAAAGTTTATTTTAATGGATACCCACCCTGCAGATAGCCACCCTGGCTCTCTCATCCCTTCCTCTGCCATCTCTGTCAAAGATATGTAGAAAACTA
**Klrk1**	NM_001083322.1	ATAAGAGGAAGATACCCTATAGAAAAACTCAAAATATCTCCAATGTTCGTTGTTCGAGTCCTTGCTATAGCCTTGGCAATTCGATTCACCCTTAACACAT

**Lag3**	NM_008479.1	GGTGCCCTCTCCCTGTTCCTTTTGGTGGCCGGGGCCTTTGGCTTTCACTGGTGGAGAAAACAGTTGCTACTGAGAAGATTTTCTGCCTTAGAACATGGGA
**Lamp1**	NM_010684.2	AGTGATTTCCAGTCTTGTGTTGGCGTTCAGGGTGGCCCTGTCTCTGCACTGTGTACAATAATAGATTCACACTGCTGACGTGTCTTGCAGCGTAGGTGGG
**Lamp2**	NM_001017959.1	GTGCCTTTCATTTTTAACATCAACCCTGCCACAACCAACTTCACCGGCAGCTGTCAACCTCAAAGTGCTCAACTTAGGCTGAACAACAGCCAAATTAAGT
**Lamp3**	NM_177356.3	AGACAGTAGTTGGGCATTCCTTCAAGTGTGTCAGTGAGCAGAGCATCCAGCTGTCAGCTCAGCTTCAAATGAAAACAATGAATATCCACCTTCAGGCCTT
**Lbp**	NM_008489.2	CCATCTCAGATTATGCCTTCAACATAGCCAGCCGGGTTTATCACCAGGCTGGGTACCTGAATTTTTCCATCACAGATGACATGTTACCACATGACTCCGG
**Lck**	NM_010693.2	TCGGGCATCAAGTTGAATGTCAACAAACTTTTGGACATGGCAGCCCAGATTGCAGAGGGCATGGCGTTCATCGAAGAACAGAATTACATACATCGGGACC
**Lcn2**	NM_008491.1	GCAGGCAATGCGGTCCAGAAAAAAACAGAAGGCAGCTTTACGATGTACAGCACCATCTATGAGCTACAAGAGAACAATAGCTACAATGTCACCTCCATCC
**Lcp1**	NM_001247984.1	TGCCCAGCCATATTACCAAGCCTCACTTTGCTGGTACTACGGAAGCTTTTTGCCACCCTAAAATTCCTGGAATTGGGGCATTGATATGTGCCAATAATCT
**Lgals3**	NM_001145953.1	ACAGGAGAGTCATTGTGTGTAACACGAAGCAGGACAATAACTGGGGAAAGGAAGAAAGACAGTCAGCCTTCCCCTTTGAGAGTGGCAAACCATTCAAAAT
**Lif**	NM_008501.2	ACATCTTTCACCTGGAAGCATTGACTTCCACCGAGCATAGTAGGTAGTGTGTCTGGACCAGAGAAAAAGGGATGGGGCATTTTGCAGTTTATCCAGAGAG
**Lilra5**	NM_001081239.2	AACACCTGGGTACTTGGGTGGAAAATTCTTCTATGGCCAATCATCCTGTTATCACTTCACCTAGATGTATAACTTTGGCTGACAGTCTCCTCTGTCTCCC
**Litaf**	NM_019980.1	TCCTGTCTCAGCTCCTGGCCTGTGAAGGGTCTCCTTTCTCATACAGTTTCTGGGAACTCCCTTTGTTCATAAACACAGATGAAAGTTACTTGGCAGGGAG
**Lrp1**	NM_008512.2	GAGCAGATGGCAATCGACTGGCTGACGGGAAACTTCTACTTTGTCGACGACATTGACGACAGGATCTTTGTCTGTAACCGAAACGGGGACACCTGTGTCA
**Lrrn3**	NM_010733.2	GCATTTCAGGGATATGATGGAAATTTGCCTCCCTCTTATAGCTCCTGAGAGCTTTCCTTCTGACCTGGATGTAGAAGCTGACAGCTATGTGTCGCTTCAC
**Lta**	NM_010735.1	AAGAGGGGAAAAATAGAAAGCCGTCAGATGACAACTAGGTCCCAGACACAAAGGTGTCTCACCTCAGACAGGACCCATCTAAGAGAGAGATGGCGAGAGA
**Ltb**	NM_008518.2	ATCAGGGACGTCGGGTTGAGAAGATCATTGGCTCAGGAGCACAGGCTCAGAAAAGACTGGATGACAGCAAACCGTCGTGCATCTTGCCCTCACCCTCTAG
**Ltbr**	NM_010736.3	AGAGAAGGGGAGGATGGGGCCTTGGGTAGCGTTAGCATGTGTGGAGGAGAGTTTGGGAACAGGAGAAAAACGACAAGTGTATTTATGAGTTGTAATCACA
**Ltf**	NM_008522.3	TGCGTATGGGTGACCATGTATGCCTCAACATAAGTTTGCACATGACTGTGTGCACTCCTTGCACTGATGTTTGCATAGGACTGTGTACTTGCACTAATGC
**Ltk**	NM_008523.2	GAGGTTTCACCAGCCAATGTCACTCTACTCAGAGCCCTTGGCCATGGTGCCTTTGGGGAAGTGTACGAGGGACTAGTGACTGGTCTTCCTGGGGACTCCA
**Ly86**	NM_010745.2	CCAGGGACAGATGTTCCCAGACCCAACAGATGTAATAAACCCTCAAAAACTATCTATTTCTGAGGACCCTGAGTAGTCTTGAAGCCCTATTGTAGTACCT
**Ly9**	NM_008534.2	ACGGTGGTGGAAACAATGTGACATACACATGGATGCCTCTACAAAACAAAGCTGTCATGTCCCAAGGGAAGTCGCACCTCAACGTCTCCTGGGAAAGTGG
**Ly96**	NM_001159711.1	ATTCTCTTTTCGACGCTGCTTTCTCCCATATTGACTGAATCTGAGAAGCAACAGTGGTTCTGCAACTCCTCCGATGCAATTATTTCCTACAGTTATTGTG
**Lyn**	NM_010747.1	CATTGAGACTAGCCTATTGTAGGTAGTTGGGAAAGCAGCCTTGGAGGAGCTAACTATCTCATGCCAGGACCCTCTGTGTTCCTGGTTCCTGATGGACGTG
**Lyve1**	NM_053247.4	AGGTGCCGACCTCGTGCAAGACCTTTCCATTTCTACATGCAGAATCATGGGCGTTGCCCTTGTGGGCAGAAACAAAAACCCACAGATGAATTTCACAGAA
**Lyz2**	NM_017372.3	TTGTGAGTTTGCCAGAACTCTGAAAAGGAATGGAATGGCTGGCTACTATGGAGTCAGCCTGGCCGACTGGGTGTGTTTAGCTCAGCACGAGAGCAATTAT
**Maf**	NM_001025577.2	CTGGCAATGAACAATTCCGACCTGCCCACCAGTCCCCTGGCCATGGAATATGTTAATGACTTCGATCTGATGAAGTTTGAAGTGAAAAAGGAACCGGTGG

**Map2k1**	NM_008927.3	CTTTGTGCTTGGGGCTATTTGTCTGTTCATCAAACACATGCCAGGCTGAACTACAGTGAAACCCTAGTGACCTGGGTGGTCGTTCTTACTGATGTTTGCA
**Map2k2**	NM_023138.4	GTCGGCACAGTGGCCGCTGCCTCTGGGGACAGTGATGCTGTTTGTTGTGGCAGGGGACCTATGCCCATGCATTTGAAAACCAAACAAAATGAAGAAAGAG
**Map2k4**	NM_009157.4	CTCGCCCAGACACCATGTGCAATAAGACTGGTGTTCGTTTCCATCATGTCTGTATACTTCTGTCATCTAGACTGTGCATCCCTGTAATACCTGACTGATC
**Map3k1**	NM_011945.2	CAGCAGATTCCTTCCGCTTACAAAGATTTGGCCGAGCCATGGATTCAGGTGTTTGGAATGGAACTCGTTGGCTGCTTATTCTCTAGAAACTGGAACGTAA
**Map3k5**	NM_008580.4	CATCATCCTCTACTGCGATACTAATTCCGATTCACTCCAGTCCCTGAAGGAAATTATTTGCCAGAAGAATACTGTGTGCACCGGGAACTACACCTTCATC
**Map3k7**	NM_009316.1	ATAACACGCCGGAAACCCTTCGATGAGATCGGTGGCCCAGCTTTCAGAATCATGTGGGCTGTTCATAATGGCACTCGACCACCACTGATCAAAAATTTAC
**Map4k2**	NM_009006.2	TTGATGCTCATGTCGAAAAGCAGCTTCCAGCCACCCAAGCTAAGAGATAAGACGCGTTGGACCCAGAATTTCCACCACTTCCTCAAGCTGGCCCTAACCA
**Mapk1**	NM_011949.3	TGTGCAGAGACCAATGTCCAAGTGTCACATCCTTTGATTGAACGAAATCTGTTGTGACCTCTGAGTTGTATTCCATGAAGAGAATGCTACCCAGAAGATA
**Mapk11**	NM_011161.5	GTGTACCTCGTGACGACCCTGATGGGCGCCGACCTGAATAACATCGTCAAGTGTCAGGCCCTGAGCGATGAGCATGTTCAATTCCTTGTCTACCAGCTGC
**Mapk14**	NM_001168513.1	GTGCTGCTTTTGATACAAAGACGGGGCATCGTGTGGCAGTTAAGAAGCTGTCGAGACCGTTTCAGTCCATCATTCACGCCAAAAGGACCTACCGAGAGTT
**Mapk3**	NM_011952.2	ACCTTAATTGCATCATTAACATGAAGGCCCGAAACTACCTGCAGTCTCTGCCCTCGAAAACCAAGGTGGCTTGGGCCAAGCTCTTTCCTAAATCTGACTC
**Mapk8**	NM_016700.3	GAAAACAGGCCTAAATACGCTGGATATAGCTTTGAGAAACTGTTCCCCGATGTGCTTTTCCCAGCTGACTCAGAGCATAACAAACTTAAAGCCAGTCAGG
**Mapkapk2**	NM_008551.1	GTAGCCTCTGCTTGCCCTTCTTGCCACGATTAACCCACTCTTGTCGAGTTCTCTGAAATTTTTAGCCATTTCTCAATGGGCTGTCCACTCCCACGGTGAG
**Marco**	NM_010766.2	GCACAGAAGACAGAGCCGATTTTGACCAAGCTATGTTCCCTGTGATGGAGACCTTCGAAATCAATGATCCAGTGCCCAAGAAGAGAAATGGGGGGACCTT
**Masp1**	NM_008555.2	CCCGGAGGGGTTTCGAATCAAGCTTTACTTCATGCACTTCAACTTGGAATCCTCCTATCTTTGTGAATACGACTATGTGAAGGTAGAAACAGAAGACCAG
**Masp2**	NM_010767.3	TCACTGGGTCCCAGCCTAAAGGTCACCTTCCACTCCGACTACTCCAATGAGAAGCCGTTCACAGGGTTTGAGGCCTTCTATGCAGCGGAGGATGTGGATG
**Mavs**	NM_144888.1	CAGAACTCAGGCTGGGCTTGCATCAGTTCCACCCTTTCCCTGGCCTTTAGCAGATGTCTAGCTTCTCTAGTCTTGTTCCCAATGTAGATAGCGGGCAGCT
**Mbl2**	NM_010776.1	CCCTGAGAAACTGGGTGCTCTTCTCTCTGAGTGAAAAAGTTGGAAAGAAGTATTTTGTGAGCAGTGTTAAAAAGATGAGCCTTGACAGAGTGAAGGCCCT
**Mcam**	NM_023061.2	CCAGTGGCTTGTACACCTTGAAGAGTGTTCTGAGTGCACGCCTAGTTAAGGAAGACAAAGATGCCCAGTTTTACTGTGAACTCAGCTACCGGCTACCCAG
**Mef2c**	NM_001170537.1	TTCTACTACTAAAGGTATCAATGGAACATGAAGACGAGTATTTAGGCAGAAGCAAAACAGGAAACCATCCTTACAAACATGCTTACCTGCACATCTGTTT
**Mefv**	NM_001161790.1	CACAGGGATTCCAGAGCATTCCATGGTGCTGGATGAAAAAACATTCAGAAACATGTCTTCCAAAACATCGTTGATTGGGGAGGAGAGATGCCCTACATCA
**Mertk**	NM_008587.1	AGGGACTTACAAAGAGCTTTCTGAAGAAGTCAGCCAGAATGGCAGCTGGGCTCAGATTCCTGTCCAAATCCACAATGCCACCTGCACAGTGAGAATCGCG
**Mfge8**	NM_008594.2	GCAACTTGGACAACAACTCCCACAAGAAGAACATCTTCGAGAAACCCTTCATGGCTCGCTACGTGCGTGTCCTTCCAGTGTCCTGGCATAACCGCATCAC
**Mif**	NM_010798.2	AACTATTACGACATGAACGCTGCCAACGTGGGCTGGAACGGTTCCACCTTCGCTTGAGTCCTGGCCCCACTTACCTGCACCGCTGTTCTTTGAGCCTCGC
**Mill2**	NM_153760.2	AAAAATGAAAGCCAGTTCTGGAAAGCCCAGAGAATTCAGACCAGCAGTCCTACTGTTGATCTTAGGGCTGCTCCTTAGAGACTCACGGGGATCCAGCATC
**Mme**	NM_008604.3	TGCTCGACTGATTCAGAATATGGATGCCTCTGTTGAGCCATGTACAGACTTCTTCAAGTATGCTTGTGGAGGCTGGTTGAAACGCAACGTCATTCCCGAG

**Mmp9**	NM_013599.2	CCTCTACAGAGTCTTTGAGTCCGGCAGACAATCCTTGCAATGTGGATGTTTTTGATGCTATTGCTGAGATCCAGGGCGCTCTGCATTTCTTCAAGGACGG
**Mnx1**	NM_019944.1	AAGTGACCTAGAAACTTGAACCCTCCTTTGGAGACAACCATTCTGCTGAAAATTTAAAACAGAGGGAGGAAAAAGGTGTTATGCCTATGTATGGGGGGGG
**Mpo**	NM_010824.2	AGTACCGATCTTACAACGACTCAGTAGACCCTCGAATCGCCAATGTCTTCACCAACGCTTTCCGTTATGGCCACACCCTCATCCAACCCTTCATGTTCCG
**Mpped1**	NM_172610.3	CCTGGCTACACACGTTTTGTCTGTGTTTCTGACACTCACTCAAGGACAGATCCCATTCAAATGCCCTACGGCGATGTGCTGATCCACGCTGGGGACTTCA
**Mr1**	NM_008209.4	TTACAAGTAGATCACAGAGTATCAGAGCCGCAAGCAACTTTCTCACCTAAGCAGGAGATGTCTTCTAAAATGTCATAGACTTGAGCATTAGCCTTCACTC
**Mrc1**	NM_008625.1	GTTCCGAAATGTTGAAGGGAAGTGGCTTTGGTTGAACGACAATCCTGTCTCCTTTGTCAACTGGAAAACAGGCGATCCCTCTGGTGAACGGAATGATTGT
**Ms4a1**	NM_007641.5	GCAACCTGCTCCAAAAGTGAACCTCAAAAGGACATCTTCACTGGTGGGCCCCACACAAAGCTTCTTCATGAGGGAATCAAAGGCTTTGGGGGCTGTCCAA
**Ms4a2**	NM_001276330.1	ACAGAAAATAGGAGCAGAGCAGATCTTGCTCTCCCAAATCCACAAGAATCCTCCAGTGCACCTGACATTGAACTCTTGGAAGCATCTCCTGCCAAAGCAG
**Msln**	NM_018857.1	AACAAGCCAAGGGGCTGGCTATGGCTGTAAGACAGAAGAACATTACACTCCGGGGACATCAGCTGCGTTGTCTGGCACGTCGCCTTCCTAGGCACCTCAC
**Msr1**	NM_001113326.1	GATTTCGTCAGTCCAGGAACATGGGAATTCACTGGATGCAATCTCCAAGTCCTTGCAGAGTCTGAATATGACACTGCTTGATGTTCAACTCCATACAGAA
**Mst1r**	NM_009074.1	GATACTCCTTATTGCTCTTCTGGTCTTGATCCTGCTTGTGGCTGTGCTGGCCGTTGCCCTGATCTTTAACTCCCGAAGACGGAAAAAGCAGCTAGGTGCT
**Muc1**	NM_013605.1	AACGGAGATTTTCTGGGGATCTCTAGCATCAAGTTCAGGTCAGGCTCCGTGGTGGTAGAATCGACTGTGGTTTTCCGGGAGGGTACTTTTAGTGCCTCTG
**Mx1**	NM_010846.1	TGGCAGAGAGACTGACTGAGGAGCTCACCTCCCACATCTGTAAATCACTGCCACTATTGGAAGATCAAATAAATAGCAGTCATCAGAGTGCAAGCGAGGA
**Mx2**	NM_013606.1	TGTCCTTCCCTGTGTTTCCTGGATTGTGATTCAGGGACAGAAGGGCTCCTGCCTTTCCTGGTAGCTATACCACCAGCCTTTATGCTCTGATAAGTGCTAG
**Myc**	NM_010849.4	CCCTCAACGTGAACTTCACCAACAGGAACTATGACCTCGACTACGACTCCGTACAGCCCTATTTCATCTGCGACGAGGAAGAGAATTTCTATCACCAGCA
**Myd88**	NM_010851.2	GCTGCAGGCTCAGCTGTTTTCTCCCCAGCAGCGAGGTTTGCATCTTCTTATTCCTTTCACGTTCTCTACCATAGAGGCAATGTCATGGTCCCTCTCAGGG
**Ncam1**	NM_001113204.1	ATGCTGTGATTGTCTGTGATGTGGTCAGCTCCCTGCCTCCAACCATCATCTGGAAACACAAAGGCCGAGATGTCATTCTGAAAAAAGACGTCCGGTTCAT
**Ncf4**	NM_008677.2	TCATGGATCGCATGGAAGCGCCAAGAGCAGAGGCCTTGTTTGACTTCACTGGGAACAGCAAATTGGAGCTAAGTTTCAAAGCTGGAGATGTGATCTTCCT
**Ncr1**	NM_010746.3	ACACCCAACCTCTGGGTTTATCCACAGCCTGAGGTAACCTTGGGGGAGAATGTTACCTTCTTTTGCCAACTGAAGACTGCCACAAGCAAATTCTTTCTGC
**Nefl**	NM_010910.1	ATAACCAGCGGCTACTCTCAGAGCTCGCAGGTCTTCGGCCGTTCTGCTTACAGTGGCTTGCAGAGCAGCTCCTACTTGATGTCTGCTCGCTCTTTCCCAG
**Nfatc1**	NM_016791.4	ATCCCGTTGCTTCCAGAAAATAACATGCGAGCCATCATCGACTGTGCTGGGATCCTGAAGCTCAGAAACTCTGATATTGAGCTGAGGAAAGGGGAGACAG
**Nfatc2**	NM_001037177.1	GGATCCTTAAGCCGCACGCCTTCTACCAAGTACACAGGATCACTGGGAAAACGGTCACCACCACGAGCTATGAGAAGATCGTAGGCAACACCAAGGTCCT
**Nfatc3**	NM_010902.1	GGGCTCACATTGTCCTTGAAGTTCCTCCCTATCATAACCCAGCAGTTACATCTGCCGTGCAGGTGCACTTTTATCTTTGCAATGGCAAGAGGAAAAAAAG
**Nfatc4**	NM_023699.3	ACAAGCGGGTGTCCCGACCAGTCCAGGTCTACTTTTACGTCTCCAATGGACGGAGGAAGCGCAGTCCTACCCAAAGTTTCAAGTTCCTACCTGTGGTCTT
**Nfkb1**	NM_008689.2	GTCTTACACTTAGCCATCATCCACCTCCACGCTCAGCTTGTGAGGGATCTGCTGGAAGTCACATCTGGTTTGATCTCTGATGACATCATCAACATGAGAA
**Nfkb2**	NM_019408.2	CCTATCACAAGATGAAGATCGAGAGGCCTGTAACGGTGTTCCTGCAGCTGAAACGCAAGCGTGGGGGCGATGTCTCGGACTCCAAACAGTTCACATATTA

**Nfkbia**	NM_01090 7.2	CTGCAGGCCACCAACTACAATGGCCACACGTGTCTGCACCTAGCCTCTATCCACGGCTACCTGGCC ATCGTGGAGCACTTGGTGACTTTGGGTGCTGATG
**Nlrc5**	NM_00103 3207.3	CCCAGGCTTAGGAGTTAACCTGACCTTGGGAGAGTTCGCACTCATCTCTTCTTTCCTGGTTTAAGG GACAAAGCCGAGCTACAGGTTTAGGGATAAGTAC
**Nlrp3**	NM_14582 7.3	ACGTGTACATCACATTCCTCTATGGTATGCCAGGAGGACAGCCTTGAAGAAGAGTGGATGGGTTT GCTGGGATATCTCTCCCGCATCTCCATTTGTAAAA
**Nod1**	NM_17272 9.2	TGGATCATCTTCCGTTGTTTCCAGCACTTCCAGACGGTCTTCGAGGGCTCCTCTTCACAGTTGCCGG ACTGTGCTGTGACCCTGACCGATGTCTTTCTGC
**Nod2**	NM_14585 7.2	TCCTTCTTGACCATCCTGACCGTGTCCTGTTAACCTTTGATGGCTTGGACGAGTTCAAGTTCCGGTT CACCGACCGGGAGCGCCACTGCTCTCCAATTGA
**Nos2**	NM_01092 7.3	CCCCCCTCCTCCACCCTACCAAGTAGTATTGTACTATTGTGGACTACTAAATCTCTCTCCTCTCCTCC CTCCCCTCTCTCCCTTTCCTCCCTTCTTCTCC
**Notch1**	NM_00871 4.2	TGAGATTGATGTTAATGAGTGCATCTCCAACCCATGTCAGAATGATGCCACTTGCCTGGACCAGAT TGGGGAGTTCCAATGCATATGTATGCCAGGTTAT
**Nrp1**	NM_00873 7.2	GGATCCGCTCCTCTTCAGGCGTTCTATCCATGGTCTTTTACACTGACAGCGCAATAGCAAAAGAAG GTTTCTCAGCCAACTACAGTGTGCTACAGAGCAG
**Nt5e**	NM_01185 1.3	AAGCATGACTCTGGTGATCAAGATATCAGCGTGGTTTCTGAATACATCTCAAAAATGAAAGTAGTT TACCCAGCCGTTGAAGGGCGGATCAAGTTCTCTG
**Nup107**	NM_13401 0.2	CCAACCAAGAGGACAATTTTGGTACTGCTACACCAAGAAGCCAGATCATTCCTCGAACGCCAAGC TCCTTTCGACAGCCTTTTGTTACTCCATCGAGCCG
**Oas2**	NM_14522 7.3	GCCCTAAGAGGCTGCTCCGATGGTACCCTTGTTCTCTTCATGGACTGCTTCCAACAGTTCCAGGAT CAGATAAAATACCAAGATGCATACCTTGACGTCA
**Oas3**	NM_14522 6.2	CCCGGGCCTGGCCTGACTTTTGAGTTTTCTCAGTCAAAGGCGTCCAGGATCTTACAGTTTCGTCTG GCATCGGCAGACGGAGAACACTGGATAGATGTTA
**Oasl1**	NM_14520 9.3	GGGTACCCAGGAAATTTCTCTCCATCCTTCAGCGAGCTGCAGCGAAACTTCGTGAAGCATCGGCC GACGAAGCTGAAGAGCCTCCTTCGGTTGGTCAAAC
**Osm**	NM_00101 3365.2	CACGGTCCACTACAACACCAGATGTCTTTAATACCAAGATAGGCAGCTGTGGCTTTCTCTGGGGAT ACCATCGCTTCATGGGCTCAGTGGGGAGGGTCTT
**Pax5**	NM_00878 2.2	TGAATCAGCTTGGGGGGGTTTTTGTGAATGGACGGCCACTCCCAGATGTAGTCCGCCAAAGGATA GTGGAACTTGCCCATCAAGGTGTCAGGCCCTGCGA
**Pdcd1**	NM_00879 8.1	AGCAGGCTTCCCGGTTTCCTATTGTCACAAGGTGCAGAGCTGGGGCCTAAGCCTATGTCTCCTGA ATCCTACTGTTGGGCACTTCTAGGGACTTGAGACA
**Pdcd1lg2**	NM_02139 6.2	ATTGTTAGGGCACTTGAGGGTGGGCGTTCTGGAAGTCCTTTCAGGTTAGTGTTTGGGGGCAGGG TTGCTCAGAATACATAAAGGTGCTAACTTAAACTGC
**Pdgfc**	NM_01997 1.2	GTTGTCACTATATCTGGTAATGGGAGCATCCACAGCCCGAAGTTTCCTCATACATACCCAAGAAAT ATGGTGCTGGTGTGGAGATTAGTTGCAGTAGATG
**Pdgfrb**	NM_00880 9.1	CTCAAGGACAACCGTACCTTGGGTGACTCCGGCGCTGGCGAGTTAGTTTTGTCTACTCGCAACAT GTCTGAGACCCGGTACGTGTCAGAACTGATCCTGG
**Pecam1**	NM_00881 6.2	ACACCTGCAAAGTGGAATCAAACCGTATCTCCAAAGCCAGTAGCATCATGGTCAACATAACAGAG CTGTTTCCCAAGCCGAAGTTAGAGTTCTCCTCCAG
**Pik3cd**	XM_003945 690.1	CCCACCAACTCTGAAATAGGAAACGTGTCTGTGTGTGTGTATGTATGTATGCCATGTTATTTATTG AAAAGAGTCTAGGTGCTCTCTGGTGTGGCTGCAG
**Pik3cg**	NM_02027 2.2	CTGAATCTCTGGACCTGTGCCTTCTGCCTTACGGTTGCATCTCAACTGGTGACAAAATAGGAATGA TCGAGATTGTAAAGGATGCCACAACGATCGCTCA
**Pin1**	NM_02337 1.3	CTTCTTAGACTTGACACAGGGGATTCTGGGTTATTGCTGCAGTAGGGAATGGCAGCCTCCTGATT ATAGCCAAACAGTCATCTATGATTTAGAGCAAGTT
**Pla2g1b**	NM_01110 7.1	GAGGACTTCATCTGCAACTGTGACCGTGAGGCCGCCATCTGCTTCTCCAAGGTCCCGTACAACAA GGAATACAAAAACCTTGACACCGGGAAATTCTGTT
**Pla2g6**	NM_00119 9023.1	CCCACAGAGGATGCAGTTCTTCGGACGCCTCGTCAACACCCTCAGTAGCGTCACCAACTTGTTCTC GAACCCATTCCGGGTGAAGGAGGTGTCCCTGACT
**Plau**	NM_00887 3.2	CAAGTCTAGGTATTTCCCTAACTCCAGACTGTGATGCGGGGCCATTTGGTCTTCCATGTGATGCTC CACGTGAATGTATCATTCCCGGGCGTGACCCGTG

**Plaur**	NM_011113.3	TTCTTTGGACCTCAGTTTTTCCATGAACCAGAAGAGAATTGGAACAAGGGCTGCGGGCAGCAGGGGCCTCTTAGTTGAGATAATATTGTTGCTGTTATTA
**Pmch**	NM_029971.2	GAAGATACTGCAGAAAGATCCGTTGTCGCCCCTTCTCTGGAACAATACAAAAACGACGAGAGCGGTTTCATGAACGATGATGACAATAAGAATTCAAAGA
**Pml**	NM_008884.5	TCCGCGACAATTCAGTGAGCAGCTTCCTCGACAGTACGCGCAAGTCCAATATCTTCTGCTCCAATACCAACCACCGCAACCCTGCGCTGACTGACATCTA
**Pnma1**	NM_027438.3	CGTGCAAGATGTTGCTCGTGTGCTTGGGTTTCAGAACCCTGCTCCAGCTCCAGGCCCGGAAATGCCAGCAGAGATGCTCAACTATATTTTGGATAATGTT
**Pou2af1**	NM_011136.2	TGCTGGAGAAGCATAACACTTGCTGTTGAGTGTTTTGCTGTCTCCTAGTGCTTTGCTCCTGAAGTGGATGTGTCTTTGGGAACTGGCCCATTGTTTACTA
**Pou2f2**	NM_001163556.1	GCCCCTGGAGGCCGAGAAGCAAAGTCTGGACTCCCCGTCAGAGCACACAGACACCGAAAGAAATGGACCCGACATTAACCATCAGAACCCCCAGAATAAA
**Pparg**	NM_011146.1	ACCAAGTGACTCTGCTCAAGTATGGTGTCCATGAGATCATCTACACGATGCTGGCCTCCCTGATGAATAAAGATGGAGTCCTCATCTCAGAGGGCCAAGG
**Ppbp**	NM_023785.2	TATCGAACTGCGCTGCAGATGTACGAATACCATCTCTGGAATCCCATTCAATTCTATCTCCCTTGTGAATGTGTACAGGCCAGGAGTTCACTGTGCTGAT
**Prdm1**	NM_007548.3	CCAATGGCTTGAGCACCATGAACAACATCAATGGTATCAACAACTTCAGCCTCTTCCCTAGGTTGTATCCCGTCTACAGTAACCTCCTTAGTGGCAGCAG
**Prf1**	NM_011073.2	ACAGCTACTGATGCCTACCTAAAGGTCTTCTTTGGTGGCCAGGAGTTCAGGACCGGTGTCGTGTGGAACAATAACAATCCCCGGTGGACTGACAAGATGG
**Prg2**	NM_008920.4	CTTCTGACTCCAAAAGCCCATTGATGGATGAGAACTTGCCTAGGGATGCAGAGATATCAGGACCAGAAGGAGAAGAGTGTCCTCCAGGGGAAGAGCTAAT
**Prkcd**	NM_011103.2	AGGACCACCTGTTCTTCGTGATGGAGTTTCTCAATGGGGGTGACCTGATGTTCCACATTCAGGACAAAGGCCGCTTCGAACTCTACCGGGCTACGTTTTA
**Prkce**	NM_011104.2	CCCGAAAATTTGATGAGCCTCGTTCTCGGTTCTATGCCGCAGAGGTCACATCAGCCCTCATGTTTCTCCACCAGCACGGAGTGATCTACAGGGATTTGAA
**Psen1**	NM_008943.2	GTAGCTAGTATCCAATAACCCAGGGGTTTCCTCATGTGATGCAAATACTACGTGTCCAACCAATCAGTGCTGTCAACGGGCTGCCATAGCTCCTTCGATG
**Psen2**	NM_001128605.1	CAAGTATCGATGCTACAAGTTCATCCATGGCTGGCTGATCATGTCCTCCCTGATGCTCCTCTTCTTGTTCACCTACATCTACCTCGGGGAAGTGCTCAAG
**Psma2**	NM_008944.2	CAGTGGGGATTAAAGCTGCAAATGGCGTAGTATTAGCAACCGAGAAAAAGCAGAAATCCATCCTGTATGATGAAAGGAGTGTTCACAAGGTGGAGCCCAT
**Psmb10**	NM_013640.3	AACCCACAGGAGGCTTCTCTTTCGAGAACTGCCAGAGGAATGCGTCCTTGGAACACGTCCTTCCGGGACTTCGGGTTCCTCATGCACGCAAGACCGGGAC
**Psmb7**	NM_011187.1	ACGAGAGCAACTGAAGGGATGGTTGTTGCTGACAAAAACTGTTCAAAAATTCACTTCATATCTCCTAATATTTATTGCTGTGGTGCTGGGACAGCTGCAG
**Psmb8**	NM_010724.2	CCTGAGGTCCTTTGGTGGTGACCAGGAAAGGAATGTTCAAATTGAGATGGCCCACGGCACAACCACACTCGCCTTCAAGTTCCAGCATGGCGTCATCGTG
**Psmb9**	NM_013585.2	TTCACCACAGATGCCATCACTCTGGCCATGAACCGAGATGGCTCTAGTGGGGGTGTCATCTACCTGGTCACCATCACAGCTGCTGGTGTGGACCATCGAG
**Psmd7**	NM_010817.2	ATTGCTAAATGGAATTCATGGATCCCATACAATGCCGCCCAACACTTGAGCTTGGACCCTTGTGAACTTTTGAGACCACACCACCTTTTGGACCTGTAAC
**Ptgdr2**	NM_009962.2	GAAGCCGCTCTGTCCACTCTTGGAGGAGATGGTCCAGCTTCCAAACCACAGCAACTCTAGCCTCCGCTACATCGACCACGTGTCGGTGCTGTTGCACGGG
**Ptgs2**	NM_011198.3	CCATCAGTTTTTCAAGACAGATCATAAGCGAGGACCTGGGTTCACCCGAGGACTGGGCCATGGAGTGGACTTAAATCACATTTATGGTGAAACTCTGGAC
**Ptprc**	NM_011210.3	AGTGGATGTCTATGGTTATGTTGTCAAGCTAAGGCGACAGAGGTGTCTGATGGTGCAAGTGGAGGCACAGTATATCCTGATTCATCAGGCTTTAGTGGAA
**Pvr**	NM_027514.2	CGAACACGGGTGACTTTCCCAACTCTGTTAAGCGCCAGGGCAATATGCTTCTAATCTCCACCGTAGAGGATGGTCTCAATAACACGGTCATTGTGTGCGA
**Pvrl2**	NM_001159724.1	GCCGATCCTGCTGCCAGTGACCCTCTCTGTGCGCTACCCTCCAGAAGTATCCATCTCCGGCTATGATGACAACTGGTACCTTGGCCGCAGTGAGGCCATA

**Pycard**	NM_023258.4	TGAAGATCGTATTGTGTGAATATAAACACGGGTGCATGTCTGCACAGAAGCTTGAAGACAAACTCCAGAGTTCTGTTTCTTACCTTGAGGTAGGGAGGAG
**Raet1c**	NM_009018.1	TACACAGAGAATATGAGCTGGAGATCAGCTAATGATGAATCAGGGGTTATCATGAATAAATGGAACGATGATGGGGACCTTGTGCAACGATTGAAATACT
**Rag1**	NM_009019.2	GAAGCACGGGAGTGGGCCCGCAGTTCCAGAAAAGGCCGTTCGTTTCTCTTTCACAGTCATGAGAATTACGATAGAGCATGGTTCACAGAACGTGAAGGTG
**Rel**	NM_009044.2	ATGGTACAGTTTTACCAGAAATGCCCAGGTCTTCAGGAGTTCCAGGGCAAGCTGAACCTTACTATTCTTCATGCGGGTCCATCTCGAGTGGATTGCCACA
**Rela**	NM_009045.4	GAGGCTGACCTCTGCCCAGACCGCAGTATCCATAGCTTCCAGAACCTGGGGATCCAGTGTGTGAAGAAGCGAGACCTGGAGCAAGCCATTAGCCAGCGAA
**Relb**	NM_009046.2	TTCTCTTTGAGCCCATTTTACAGAATGCTGAGTCCGAAGAGGAAAAGGGGCTCCTGCAGATGGACCCCTTCTCAGGACAGATTCTCAGAGATTGTACATA
**Reps1**	NM_001111065.1	ACACTGTAAAGGATCTCCCTCTCCCACGATTTGTTGCTTCAAAGAATGAACAGGAATCTCGCCTTGCAGCCTCCTATTCTTCAGATTCTGAAAACCAGGG
**Ripk2**	NM_138952.3	CCAGGGACATCGACCTGACACCAGTGAGGAGAATTTGCCATTTGATATACCTCATCGAGGTCTCATGATCTCTCTAATACAGAGTGGATGGGCGCAAAAC
**Rora**	NM_013646.1	GTGTCAAAATGATCAAATTGTGCTTCTAAAAGCAGGCTCGCTAGAGGTGGTGTTTATTAGGATGTGCCGTGCCTTTGACTCTCAGAACAACACCGTGTAC
**Rorc**	NM_011281.2	GGGGCCTCCTGCCACCTTGAGTATAGTCCAGAACGAGGCAAAGCTGAAGGCAGAGACAGCATCTATAGCACTGACGGCCAACTTACTCTTGGAAGATGTG
**Rps6**	NM_009096.3	GATTAAACCTAGGCCTCAGTCATGCTTCCCAGTCACTGGTACTGATTTGTATGCACCCGCTTAGGTGTGAAGGTAGTTTTGGTGTGTATCACAAGTTAGC
**Rrad**	NM_019662.2	GAACCCAGCTTGTGGACAGGACTCTGCCTTTCTCTATGCGGGTGTCTGTGCCCTGGAGGGAGGCTGTTCAATATGGTTGCTGTTTGTTTACATGCAGATT
**Rsad2**	NM_021384.4	AGCCTTATCCGGGAGAGATGGTTCAAGGACTATGGGGAGTATTTGGACATTCTTGCTATCTCCTGCGACAGCTTCGATGAGCAGGTTAATGCTCTGATTG
**Runx1**	NM_001111021.1	ACAGTGGAGGACTTGGTCAAAATCCAGTTGTTCTACAACGTATGAAGCCTAACCGCTGGTTCTGACATACATGTGCTCAAAATGATCTGGTTGTTTGGAT
**Runx3**	NM_019732.2	GATGTGCCGGATGGAACGGTGGTGACCGTGATGGCCGGCAATGATGAGAACTACTCCGCCGAGCTGCGCAACGCTTCCGCTGTCATGAAGAACCAAGTGG
**S100a8**	NM_013650.2	TGGACATCAATAGTGACAATGCAATTAACTTCGAGGAGTTCCTTGCGATGGTGATAAAAGTGGGTGTGGCATCTCACAAAGACAGCCACAAGGAGTAGCA
**S100b**	NM_009115.3	ACCGAGAATCAAAATTCTGCTCGGCAGACTTCTCCTTTCAGGATGATCGCTTTGTTCCTGGAGGACAGAGGAGGGGAATGGCCAGAGTCTTTTTCTAGTT
**Saa1**	NM_009117.3	GGCAAAGACCCCAATTACTACAGACCTCCTGGACTGCCTGACAAATACTGAGCGTCCTCCTATTAGCTCAGTAGGTTGTGCTGGGGGCCTGAGGGTGGGG
**Sbno2**	NM_183426.1	GAGTGCAGACCGTGCCATTCAACAGTTTGGCCGCACCCACAGGTCCAACCAGGTCTCAGCACCCGAGTACGTCTTCCTTATCTCAGAACTGGCAGGGGAG
**Sele**	NM_011345.2	TCAGTGGCTGCCGAAGTATTCTTGAACATTGTGTTCTGTGTCCTGGCACTGAAGCCAGCATGAGATCCATCATTCTTATGTCAGCTCAAGGGTCAAAAGG
**Sell**	NM_001164059.1	GTCCAAGTGTGCTTTCAACTGTTCTGAGGGAAGAGAGCTACTTGGGACTGCAGAAACACAGTGTGGAGCATCTGGAAACTGGTCATCTCCAGAGCCAATC
**Selplg**	NM_009151.3	GCTTTCCCAGGTCCCTGGCTTGGAGGGATGGTCCTTCCTTTGGGCCCGTGTGAACCAACGAGTTTCCGTACAGTGACAGAATGACCTCGCGCTGCGGCCT
**Serpinb2**	NM_001174170.1	TGGGCTTTATCCTTTCCGTGTGAACTCGCATGAGAGCATACCTGTCCAGATGATGTTCCTCCATGCAAAGCTGAACATTGGATACATAAAGGACCTGAAG
**Serping1**	NM_009776.3	TCTGCCATCTCCTTTGGCCGAAGCTTACCCATCTTTGAGGTGCAGCGACCTTTCCTCTTCCTGCTCTGGGACCAGCAACACAGGTTCCCAGTCTTCATGG
**Sh2b2**	NM_018825.3	GGAAATGCCTGAAAAGGACAACACATTTGTGCTCAAGGTGGAGAACGGAGCAGAATACATCCTGGAAACGATAGACTCACTGCAGAAGCACTCGTGGGTG
**Sh2d1a**	NM_011364.3	TTGTATCAAGGTTACATCTACACATATCGAGTGTCCCAGACAGAAACAGGTTCTTGGAGTGCCGAGACAGCACCTGGAGTACATAAAAGATTTTTCCGGA

**Sh2d1b1**	NM_012009.4	ATCTAAGGATACTGAAGGAGCAAACATGTCCTTCCTACCTGTGAGGTCGTGGTCCTGATGGGGGCTGTGACCCAACCTGAAACTGTAGGTTTTTTCACCT
**Sigirr**	NM_023059.3	CTGGGTCAGCGCCAACTTCTCAGAGATTGTGTCCAGTGTCCTGGTGCTCAACTTGACCAATGCAGAGGACTATGGAACCTTCACCTGTTCTGTCTGGAAT
**Siglec1**	NM_011426.3	ATTGGCTTCAGGAGGGACCGGCTTCCTCACTCCAGTTCCTGGTGACTACACGGGCTCACGCTGGTGCTTACTTTTGCCAGGTGCATGATACACAAGGCAC
**Slamf1**	NM_013730.4	AAGTGTGGTTCTCTGATTAAGTCAAAGCAACACTGTTTGGCAATGCTGCTGTAAAGTTGCCTGGAATACTCAGAGGAACTTGTCCCAGGGAGGTTTTTTT
**Slamf6**	NM_030710.2	CCAAGAATGCTGTCAGCAATTTGTCAGTCTCTGTTTCGACCCAGAGTCTCTGCAAAGGGGTTCTAACTAATCCACCCTGGAATGCAGTATGGTTTATGAC
**Slamf7**	NM_144539.5	TACCGTGCAGAGATTTACAGTACATCGAGTCAGGCTTCCTTAATCCAGGAGTATGTGCTGCATGTCTACAAGCATTTGTCAAGGCCCAAGGTCACCATAG
**Slc11a1**	NM_013612.2	CATGTACTTCCTGATTGAGGCCACCATCGCCCTATCGGTGTCCTTCATCATCAACCTCTTCGTCATGGCTGTTTTTGGTCAGGCCTTCTACCAGCAAACC
**Slc7a11**	NM_011990.2	CCGGGGTCGGTTTTCTTATCACCTTGACTGGGGTCCCTGCATATTATCTCTTCATTGTATGGGACAAGAAACCCAAGTGGTTCAGACGATTATCAGACAG
**Smad2**	NM_010754.4	TCACAGCTTGGATTTGCAGCCAGTTACTTACTCGGAACCTGCATTCTGGTGTTCAATCGCATACTATGAACTAAACCAGAGGGTTGGAGAGACCTTCCAT
**Smad3**	NM_016769.3	GTGTATCGCCACCTGACTCCTTGTTTAATGACAGAGGTCTGGGATGTCACAGTCCAAAAGGAAAGTGCCTTTCTCCATGGCTGGAGTATGGAGTTTACCT
**Smad4**	NM_008540.2	TCCTAGGGAGAAGGTTTTGTATAAAACACTAAAAGCAGTGTCACTCTGCCTGCTGCTTCACTGTTCTGCAAGGTGGCAGTACTTCAACTGAAATAATGAA
**Smn1**	NM_011420.2	GAAACCTGTGTCGTGGTTTATACTGGATATGGAAACAGAGAGGAGCAAAACTTATCTGACCTACTTTCCCCGACCTGTGAAGTAGCTAATAGTACAGAAC
**Smpd3**	NM_021491.3	TAAGTTGAAAGAGCAGCTACACGGCTACTTCGAGTACATCCTGTATGATGTTGGGGTCTACGGTTGTCATGGTTGCTGCAATTTCAAATGTCTCAACAGC
**Snai1**	NM_011427.2	TCACTGCCAGGACTCCTTCCAGCCTTGGTCCGGGGACCTGTGGCGTCCATGTCTGGACCTGGTTCCTGCTTGGCTCTCTTGGTGGCCTTTGCCGCAGGTG
**Socs1**	NM_009896.2	CAGCTTGTGTCTGGGGCCAGGACCTGAATTCCACTCCTACCTCTCCATGTTTACATATTCCCAGTATCTTTGCACAAACCAGGGGTCGGGGAGGGTCTCT
**Socs3**	NM_007707.2	CCGCGACAGCTCGGACCAGCGCCACTTCTTCACGTTGAGCGTCAAGACCCAGTCGGGGACCAAGAACCTACGCATCCAGTGTGAGGGGGGCAGCTTTTCG
**Spink5**	NM_001081180.1	TTCCAGCTCCTCTAGGGACTCTGACATGTGCAAAAACTACCGAATATTACCCAGAATGGGTTATCTTTGCCCAAAGAATCTAAACCCTGTCTGTGGTGAT
**Spn**	NM_001037810.1	GCAACCAGTTCTGTGGAGAGTTCCAGTGTGGCCCGTGGCACCTCAGTTTCCAGCAGAAAAACATCCACGACGTCTACCCAAGATCCCATAACCACCAGGT
**Spp1**	NM_009263.3	TGAATCTGACGAATCTCACCATTCGGATGAGTCTGATGAGACCGTCACTGCTAGTACACAAGCAGACACTTTCACTCCAATCGTCCCTACAGTCGATGTC
**St6gal1**	NM_001252506.1	GCCAGGCATCCTCATCCTTCTCCATAAGTCATTTTATGGCTGCTCTCCTGGTTACCACTGCTTGAAGGAGTGTTTTTATTCAACAGGCCCAGCCTGCTTC
**Stat1**	NM_009283.3	ACGCTGGGAACAGAACTAATGAGGGGCCTCTCATTGTCACCGAAGAACTTCACTCTCTTAGCTTTGAAACCCAGTTGTGCCAGCCAGGCTTGGTGATTGA
**Stat2**	NM_019963.1	AGTGGGACCACTACAGCTCAGACTCCAATCACTTTTTATTACAGCATAACTTGCGAAAATTCAGCCGGGATATTCAGACCTTTCCCAATGGCCCTACCCA
**Stat3**	NM_213659.2	GGGGTCACTTTCACTTGGGTGGAAAAGGACATCAGTGGCAAGACCCAGATCCAGTCTGTAGAGCCATACACCAAGCAGCAGCTGAACAACATGTCATTTG
**Stat4**	NM_011487.4	TAGAGACCAGCTCATTACCTGTCGTGATGATTTCTAATGTCAGCCAACTACCTAATGCATGGGCATCCATCATTTGGTACAATGTATCAACTAACGACTC
**Stat5b**	NM_011489.3	TGAGGGTGTACCTGGACATGGGAGAGGTTTTTAACTGGAAAGTGTGTCCCCTATCTGCATGCTGGTCTCTCTCTCTCTGCCCAACTCTTGCACCCAAA
**Stat6**	NM_009284.2	CATTGTATAAGACAGCAACCCAGTATCATTTGGGGAGTAACTATGTGGCTGTGACATGCATAAAGCTCTAGCCTGGGTAACTTGATGCTTCCAGTGTTTC

**Syk**	NM_001198977.1	TGAGGCTTCGCAATTACTACTACGACGTGGTTAACTAACAGCTCCGGCGCCTGTCCGTGCACACCACGGATTCCCAAGCGATCACAGGAAATTCATTCAG
**Syt17**	NM_138649.1	AACTGGAAAACGCCAGCCTAGTATTCACAGTGTTCGGCCACAACATGAAAAGCAGCAATGACTTCATCGGGAGGATCGTCATCGGCCAGTATTCCTCCGG
**Tab1**	NM_025609.2	TGTGCAAATCTACAGTGGATGGGTTACAGGTTACACAGCTAAACATGGACCACACCACCGAGAACGAGGACGAGCTCTTTCGGCTTTCGCAACTGGGTTT
**Tal1**	NM_011527.2	CCGATCTATCCTAAAGCTAGGCCTGCCTATAATGAAGTTCAAGCTCATGAAACAAAGTGCGCAGAAGTCCTGTGTATCTGTCATTGTATTGGTGTCACTC
**Tank**	NM_011529.1	CAATCAAAGGTGAGACGACAAGAAGTTTCTTCTGGAAAAGAATCCGCCAAGGGTCTCAACATCCCTCTGCATCACGAAAGGGATAATATAGAGAAGACTT
**Tap1**	NM_001161730.1	TGGCCATTCCCTTCTTCACGGGCCGCATCACTGACTGGATTCTTCAGGATAAGACAGTTCCTAGCTTCACCCGCAACATATGGCTCATGTCCATTCTCAC
**Tap2**	NM_011530.2	TCCAGGCCGGACCTGCCTTTCCTCATAGCTGCCTTCTTCTTCCTTGTGGTGGCTGTGTGGGGGGAGACATTAATCCCTCGCTATTCGGGTCGTGTAATTG
**Tapbp**	NM_009318.2	AGGGAGTTTCAGGTCAGTCGGGGTTACTTGGCGGGGCTTTGTTGAGAGAACTAAATATAAGAACACACATGCGTAGAGTCCCAGGGTTGAGAAGAACCAG
**Tbk1**	NM_019786.4	ATACACTGTTCTAGAGGAGCCGTCCAATGCGTATGGACTTCCAGAATCAGAATTTCTCATTGTCTTACGAGATGTGGTGGGCGGGATGAATCATCTCCGA
**Tbx21**	NM_019507.1	CACTAAGCAAGGACGGCGAATGTTCCCATTCCTGTCCTTCACCGTGGCTGGGCTGGAGCCCACAAGCCATTACAGGATGTTTGTGGATGTGGTCTTGGTG
**Tcf7**	NM_009331.3	CTTTCCCAAGAAGCTCACCAGCATTAACAACTAGTCAATATAGTTGGCCTAAACCCAGTGTGCACCCTTCCTATCAGGCTCTTCCCAGTTCCATTTCCAG
**Tdo2**	NM_019911.2	AGAGTCTACAGTTCCGGCTGCTGGAAAATAAGATTGGTGTTCTTCAGAGCTTGAGAGTCCCTTACAACAGGAAACACTATCGTGATAACTTTGGAGGAGA
**Tek**	NM_013690.2	GAGACCATCATTTGCCCAGATATTGGTGTCCTTAAACAGGATGCTGGAAGAACGGAAGACATACGTGAACACCACACTGTATGAGAAGTTTACCTATGCA
**Tfe3**	NM_172472.3	GAGGACTCTGTGCTGACCTTATTCTTGGGGAAGGGAACAGATTTTGACCTAGATCAAGTGTAACTTAGGTCCTCACCCTTGGGACGACCTTGCCTTTTCT
**Tfeb**	NM_001161723.1	CCTACCACCTGCAACAGTCCCAGCATCAGAAGGTTCGGGAGTATCTGTCTGAGACCTATGGGAACAAGTTTGCTGCCCACGTGAGCCCAGCCCAAGGTTC
**Tfrc**	NM_011638.3	CTGAACCAGTTCAAAACAGATATCAGGGATATGGGTCTAAGTCTACAGTGGCTGTATTCCGCTCGTGGAGACTACTTCCGTGCTACTTCTAGACTAACAA
**Tgfb1**	NM_011577.1	GGAGTTGTACGGCAGTGGCTGAACCAAGGAGACGGAATACAGGGCTTTCGATTCAGCGCTCACTGCTCTTGTGACAGCAAAGATAACAAACTCCACGTGG
**Tgfb2**	NM_009367.1	CCCAAAGCCAGAGTGGCCGAGCAGCGGATTGAACTGTATCAGATCCTTAAATCCAAAGACTTAACATCTCCCACCCAGCGCTACATCGATAGCAAGGTTG
**Tgfb3**	NM_009368.2	TCATGTAATTAGTTTCTGGGCCAGCAACTAGCTATCTCAGGTCCCTTAGAGATGCTGGACTCAAAAGCAGAGGTCAGAATTGGTTCTCTCATGTATTCCC
**Tgfbr1**	NM_009370.2	TCAGAAGTAGTGGCCAGCTGTGTCTCTAGTAGGACAGTAAAGGCATGAAGCTCAGCCTGTAATCCTGCTACTACAGTAGTACTCCAGAAGTGCCTTGAGG
**Tgfbr2**	NM_009371.2	TGTGCAAGTTTTGCGATGTGAGACTGTCCACTTGCGACAACCAGAAGTCCTGCATGAGCAACTGCAGCATCACGGCCATCTGTGAGAAGCCGCATGAAGT
**Thbd**	NM_009378.3	CCCTTTGACATGACAATAGGACATTGCTATCTTGAGACATACTGGGCCACATTCATAGCTTTCCAAGGATGTATGTGGTCCTGCCTCAACATATCAGAGC
**Thbs1**	NM_011580.3	TGGAGAGCGCGGAGCTGGATGTACCCATCCAGAGCATCTTCACCAGGGATCTGGCCAGCGTTGCCAGGCTCCGAGTTGCAAAGGGAGATGTCAATGACAA
**Thy1**	NM_009382.3	ACTTTTGTGAGCTTCAAGTCTCGGGCGCGAATCCCATGAGCTCCAATAAAAGTATCAGTGTGTATAGAGACAAGCTGGTCAAGTGTGGCGGCATAAGCCT
**Ticam1**	NM_174989.4	TCCAGTCTCTTCCCCACAGTCCCAATCCTTTCCATCAGCCTCCTCCCCAGCCCCACAGACTCCAGGACCTCAGCCTCTCATTATTCACCATGCCCAGATG
**Ticam2**	NM_173394.2	TTCTAAGAAATGCTTAGACGTGAGTCTGCTTCCAAGAAATCTGTGGCACACAAGAAAGGTACACTTCTGCAGCCCTAGGATTGGGCTACTTTCGAATGAC

**Tie1**	NM_011587.2	GGGCTCAAGATGAACGCAGCCATCAAGATGCTAAAAGAGTATGCGTCTGAAAATGACCATCGAGACTTTGCAGGTGAACTAGAAGTTCTGTGCAAACTAG
**Tigit**	NM_001146325.1	GCAGAAGATGACTATGCTGACCCACAGGAATACTTTAATGTCCTGAGCTACAGAAGCCTAGAGAGCTTCATTGCTGTATCGAAGACTGGCTAACGACAGC
**Timd4**	NM_178759.4	GAATCATCTCCAGGAAGTCAACAAAATATACACTTTTGGGGAAGGTCCAGTTTGGTGAAGTGTCCTTGACCATCTCAAACACCAATCGAGGTGACAGTGG
**Tirap**	NM_001177846.1	AGGAGGTGCAAACCCATGCAATCTACCTGGAATCGGCTGTCTTCACCATTTTATGACTTTGTTTGCCTAGCTGACGGATCTCAAATGCCCTCCTGGCCAG
**Tlr1**	NM_030682.1	TCAGCACTACGATCGGTTTGGAACTGTCTAACATCAAGTGTGTGCTTGAAGACCAGGGCTGCTCTTATTTCTTACGTGCTTTGTCAAAGCTTGGAAAGAA
**Tlr2**	NM_011905.2	GCAGGCGGTCACTGGCAGGAGATGTGTCCGCAATCATAGTTTCTGATGGTGAAGGTTGGACGGCAGTCTCTGCGACCTAGAAGTGGAAAAGATGTCGTTC
**Tlr3**	NM_126166.2	TTTCTCTGGGCTGAAGTGGACAAATCTCACCCAGCTCGATCTTTCCTACAACAACCTCCATGATGTCGGCAACGGTTCCTTCTCCTATCTCCCAAGCCTG
**Tlr4**	NM_021297.2	AACGGCAACTTGGACCTGAGGAGAACAAAACTCTGGGGCCTAAACCCAGTCTGTTTGCAATTAATAAATGCTACAGCTCACCTGGGGCTCTGCTATGGAC
**Tlr5**	NM_016928.2	CTGGGGACCCAGTATGCTAACTTGACCATTGGTCCAGGGGCTTTCAGAAACCTGCCCAATCTTAGGATCTTGGACTTGGGCCAAAGCCAGATCGAAGTCT
**Tlr6**	NM_011604.3	ACCTGGATGTCTCACACAATCGGTTGCAAAACATCTCTTGCTGCCCTATGGCGAGCCTGAGGCATCTAGACCTCTCATTCAATGACTTTGATGTACTGCC
**Tlr7**	NM_133211.3	TCTGCAGGAGCTCTGTCCTTGAGTGGCCTGCAAATCCACAGGCTCACCCATACTTCTGGCAGTGCCTGAAAAATGCCCTGACCACAGACAATCATGTGGC
**Tlr8**	NM_133212.2	TCCATAAAGCGAACTATTCCAGAAGCTATCCTTGTGACGAGATAAGGCACAACTCCCTTGTGATTGCAGAATGCAACCATCGTCAACTGCATGAAGTTCC
**Tlr9**	NM_031178.2	GGCCACAATTTCAGTTTTGTGACCCATCTGTCCATGCTACAGAGCCTTAGCCTGGCACACAATGACATTCATACCCGTGTGTCCTCACATCTCAACAGCA
**Tmed1**	NM_010743.3	GCGAGTTCACATTTCTGCTTCCCGCCGGGAGAAAGCAGTGTTTCTATCAGTCCGCACCGGCCAATGCTAGTCTTGAGACCGAGTACCAGGTGATCGGAGG
**Tmem173**	NM_028261.1	GCAGACTTCCAACCCTCCAGCTTCTGGTCACGTGTGTTCAATGGGAGCTTAAGTAGATGGCGAGAGGGAGAAGGAACATTTGTTCTGTTAGCTGTATACA
**Tnf**	NM_013693.1	TTCCTGAGTTCTGCAAAGGGAGAGTGGTCAGGTTGCCTCTGTCTCAGAATGAGGCTGGATAAGATCTCAGGCCTTCCTACCTTCAGACCTTTCCAGACTC
**Tnfaip3**	NM_009397.2	GGCTGAACAACTTCTTCCTCAGGCTTTGTATTTGAGCAATATGCGGAAAGCTGTGAAGATACGAGAGAGAACCCCAGAAGACATTTTCAAACCTACCAAT
**Tnfrsf10b**	NM_020275.3	CTGGTTCACATAAGATCCCATATAATGTGCAGGATGGCTAAACTTGCTGAGAGCTGACTCTGTGGTCTCCTGTCCCAGATTCTAGCGATATTCATTACTA
**Tnfrsf11a**	NM_009399.3	ATCTCTGTGGTAGTAGTGGCTGCCATCATCTTCGGCGTTTACTACAGGAAGGGAGGGAAAGCGCTGACAGCTAATTTGTGGAATTGGGTCAATGATGCTT
**Tnfrsf11b**	NM_008764.3	CCGGAGTGTCCCAAAGCCCCACCACTAGCTCCCAAGGTTCCTAAAAAGGAACAAGGTTTGTCCAGACAGAGACTAATAGATCAAAGGCAGGGCATACTTC
**Tnfrsf12a**	NM_001161746.1	AGCCTCAATCTGGGTCACAAAGCGACCCATACTAAGGAACTGCAGCATTTGCACAAGGGAATCTCTTGTTCCCCACAAGTCCTGGCAGTCTGGCTGACTT
**Tnfrsf13b**	NM_021349.1	GTCCAGGATTGAGGCTAAGTAGCGACCAGCTGACTCTCTACTGCACACTGGGGGTCTGCCTCTGCGCCATCTTCTGCTGTTTCTTGGTGGCCTTGGCCTC
**Tnfrsf13c**	NM_028075.2	AATGCACTGCGGGTGGCTCATTACTGAGCATAACTTTTGCTTCAGGGGACCTGATGCCTCTGGACTTCATGGGCATCTGTATTCACGTGCACATCCTACA
**Tnfrsf14**	NM_178931.2	ACAGACACCACCTGCTCCTCCCAGGTCGTCTACTACGTTGTGTCCATCCTTTTGCCACTTGTGATAGTGGGAGCTGGGATAGCTGGATTCCTCATCTGCA
**Tnfrsf17**	NM_011608.1	CCTCATGGCGCAACAGTGTTTCCACAGTGAATATTTTGACAGTCTGCTGCATGCTTGCAAACCGTGTCACTTGCGATGTTCCAACCCTCCTGCAACCTGT
**Tnfrsf18**	NM_009400.2	CTCCCCTCAACAGTGGCGGAAGTGGGTGTATGAGAGCGGTGAGTTACGATTGGGCCCTATGGCTGCCTTTCTCATTTGACAGCTCTGTTGGAGTAGGGTC

**Tnfrsf1a**	NM_011609.2	CTCCTTGCCAAGCTGACAAGGACACGGTGTGTGGCTGTAAGGAGAACCAGTTCCAACGCTACCTGAGTGAGACACACTTCCAGTGCGTGGACTGCAGCCC
**Tnfrsf1b**	NM_011610.3	GTGTGTGTCCATGTTTGCATGTATGTGTGTGCCAGTGTGTGGAGGCCAGAGGTTGGCTTTGGGTGTGTTTGATCACTCTCAGTTACTGAGGCAGGGCTCT
**Tnfrsf4**	NM_011659.2	ATGGTGAGCCGCTGTGATCATACCAGGGATACTCTATGTCATCCGTGTGAGACTGGCTTCTACAATGAAGCTGTCAATTATGATACCTGCAAGCAGTGTA
**Tnfrsf8**	NM_009401.2	GAGCCCCGGGTATCCACGGAACACACCAATAACAGGATTGAGAAAATCTACATCATGAAGGCCGACACAGTGATCGTGGGCTCTGTAAAAACTGAAGTCC
**Tnfrsf9**	NM_001077508.1	TCTTCAGAGCAGTTCAAGGGCCTGCTTCTCCTGTTTCCTCTGTGTCAGGCTTTTCAATAAAAAGGCCGTTTAGGAAAGGGACAAAGCACTGTGAGGTGGG
**Tnfsf10**	NM_009425.2	GTTCACCATGCTTGCTTTGTCCAGATTGCGACTGTCACCCAGTCCTCTGGCTCTTCCATCTGTCTGTCCACTCCACCTACCAAGATGTTGAACACTTGTT
**Tnfsf11**	NM_011613.3	CCAGCGAGGCAAGCCTGAGGCCCAGCCATTTGCACACCTCACCATCAATGCTGCCAGCATCCCATCGGGTTCCCATAAAGTCACTCTGTCCTCTTGGTAC
**Tnfsf12**	NM_011614.3	TCAAAGGCAGCCAGAGCTTGTTCACATGTTTTCCATTCCACAGACGTATCCTTGCTCTTCTTTAACATCCCATCCCACCACAACTATCCACCTCACTAGC
**Tnfsf13**	NM_023517.2	AAAAAGATATATGTTAGGTGCCTCGATATGCATGCCATTCATCCTCCCCATTCTCCTATACACTTCCGAGCTGGGCACTGAGCTTTACGCCTTAAATCAC
**Tnfsf13b**	NM_033622.1	TCTGCAAAGACCCTGCCACCACCGTGCCTCTGTTTTTGCTCCGAGAAAGGAGAAGATATGAAAGTGGGATATGATCCCATCACTCCGCAGAAGGAGGAGG
**Tnfsf14**	NM_019418.2	CAGGAAGAAAGAATCAAGCTGGGGTATTTATGCTTCTGATGCAAACACTGAGATTTCGGCTTTCTGGGTTTTGAGCTGGAGGCAAGAAACCTTCCCAGAG
**Tnfsf15**	NM_177371.3	AAGACTCACAAGCCCGAGTTGCAGTTACTTTTCTGAAGCAACATAGTATGTTAATGGAATGGCCAGAACTCTACTCTTGGCACATGGCACTGAATTTGAT
**Tnfsf18**	NM_183391.3	GCACAGTGAGAACATGGGTAGATGAACCCTAAGACTCTTACCTCAATTCAGAACTCGCAAGGAGTTAAGTGAGTGGGGTCTTCATTAGACCATTCACATG
**Tnfsf4**	NM_009452.2	CCAGATGTGAGGATGGGCAACTATTCATCAGCTCATACAAGAATGAGTATCAAACTATGGAGGTGCAGAACAATTCGGTTGTCATCAAGTGCGATGGGCT
**Tnfsf8**	NM_009403.2	ATCCAGGATGCAGGGGAAAATCCTTCTTGGAACAGAGCTGGGTACAGAACCGAATCAGATGAGGAGAGATAAGGTGTGATGTGGGACAGACTATATAAAG
**Tollip**	NM_023764.3	CTCAGCATCACTGTGGTACAGGCAAAATTGGCAAAGAATTATGGCATGACTCGTATGGACCCTTACTGCCGTCTGCGTCTGGGCTATGCTGTTTATGAAA
**Tpsab1**	NM_031187.4	GGCTGGGGTAACATCGACAATGGTGTAAACCTGCCGCCACCATTTCCTTTGAAGGAGGTGCAAGTTCCCATTATAGAAAACCACCTTTGTGACTTGAAGT
**Traf2**	NM_009422.2	GGCCTTTTAATCAGAAGGTAACATTGATGTTGCTGGACCATAACAACCGGGAGCATGTGATCGACGCATTCAGGCCCGATGTAACCTCGTCCTCCTTCCA
**Traf3**	NM_001048206.1	GGTTGGGGTCAGTGCAGACAGCTGCAGACTGTTCTAAAGTTCCGTTCTTATCTCTGATGGCACACCTCTCGTGTTCAGTTCTATTACCCAAAAAACAAAG
**Traf6**	NM_009424.2	GTGCTGTGTCCATGGCATATGAAGAGAAAGAGATCCATGATCAAAGCTGTCCTCTGGCAAATATCATCTGTGAATACTGTGGTACAATCCTCATCAGAGA
**Trem1**	NM_021406.5	GTGGCTCCTGTGCTTTCTCTAGGACACACTGGCATTTGCTTGTTTCCACCTCTTTGGGTGGGAAAAGGATGGTAGATCCATTCCTTCTACTTCAGCAGTG
**Trem2**	NM_031254.2	GAATCAAGAGACCTCCTTCCCACCCACCTCCATTCTTCTCCTCCTGGCCTGCGTTCTCCTGAGCAAGTTTCTTGCAGCCAGCATCCTCTGGGCTGTGGCC
**Trp53**	NM_011640.1	CCCTCTCTGAGTAGTGGTTCCTGGCCCAAGTTGGGGAATAGGTTGATAGTTGTCAGGTCTCTGCTGGCCCAGCGAAATTCTATCCAGCCAGTTGTTGGAC
**Twist1**	NM_011658.2	AATGGACAGTCTAGAGACTCTGGAGCTGGATAACTAAAAATAAATCTATATGACAAAGATTTTCATGGAAATTAGAAGAGCAGAGACCAAATTCACAAGA
**Txk**	NM_001122754.1	TTACCAGCCACATCTGGTTTTAGCTATGAAAAGTGGGAGATAGATCCATCAGAGTTGGCTTTTGTCAAGGAGATCGGAAGTGGTCAGTTTGGGGTTGTCC
**Txnip**	NM_023719.1	CCTGAGTGCTGCGATCAAAGGCCCAGCTTGGTTATTGCTTTTGAGGCTTTCTCCCAACGCACAGACTTGTGTAATTCTAACACTAATCCTGTGAAGGGTT

**Tyk2**	NM_001205312.1	ACAAGTGCTTGTTGCTGTGCCTCTGTTCCCAGGCTGAGGCCCTGTCCTTTGTGGCCCTGGTCGATGGCTATTTCCGCTTGACTGCTGACTCCAGCCACTA
**Ubc**	NM_019639.4	AAGACCATCACCCTGGACGTCGAGCCCAGTGTTACCACCAAGAAGGTCAAACAGGAAGACAGACGTACCTTCCTCACCACAGTATCTAAAAAGAGCCCTC
**Ulbp1**	NM_029975.2	GGCAACTGGACCCAACTGGATCATGAATTCGAGAAGTTTATAGAAATGTGCAAGGAAGACAAGGTTTTAGCTGCCTTTTTAAAGAAGACTACAGAGGGCG
**Usp18**	NM_011909.2	GGCTGACTTTGGTCATTACTGTGCCTACATCCGGAATCCCGTGGATGGAAAGTGGTTCTGCTTCAATGACTCACATGTTTGTTGGGTCACCTGGAAGGAT
**Usp9y**	NM_148943.2	TGGACAACCTCAGCCTTCTTTCCAACAGAACCAGATCTCATCATCTGATTCTTCCAATGAGACTTCTCCAACAACTCCTCCATATGAACAAGGCCAAGGT
**Vcam1**	NM_011693.2	GGTTTTGAGGATGAACACTCTTACCTGTGCGCTGTGACCTGTCTGCAAAGGACACTGGAAAAGAGAACCCAGGTGGAGGTCTACTCATTCCCTGAAGATC
**Vegfa**	NM_001025250.3	TCTCTCTCTCCCAGATCGGTGACAGTCACTAGCTTGTCCTGAGAAGATATTTAATTTTGCTAACACTCAGCTCTGCCCTCCCTTGTCCCCACCACACATT
**Vegfc**	NM_009506.2	TACAGAAAACACACAGAAGTGCTTCCTTAAAGGGAAGAAGTTCCACCATCAAACATGCAGTTGTTACAGAAGACCGTGTGCGAATCGACTGAAGCATTGT
**Vhl**	NM_009507.3	GGCTTTGTAGTGTTTAAGAATAAACATGCAAAGTGCCACTGCGTCTGCCCTTTGTAGAGCACTCACCCGAGGGAGGAAGACGTTTTCAGTTTTGCTTCCT
**Vim**	NM_011701.4	CAGCGCTCCTACGATTCACAGCCACCGCGCCCTCATTCCCTTGTTGCAGTTTTTCCAGCCGCAGCAAGCCAGCCCACCTTCGAAGCCATGTCTACCAGGT
**Vwf**	NM_011708.3	GTCAGCATCTACGGCGCTATCATGTATGAAGTCAGGTTTACCCATCTTGGCCACATCCTCACATACACGCCACAAAACAACGAGTTCCAACTGCAGCTTA
**Xaf1**	NM_001037713.3	CTCCACTTCATGCTCCACGAGGCCCACTGCCTGCGCTTCATAGTCCTTTGCCCAGAATGTGAAGAGCCCATCCCAGAGTCAAAGATGAAAGAGCACATGG
**Xbp1**	NM_013842.2	CCTCTAGTTTTAGAGATCCCCTCTGAGACAGAGAGTCAAACTAACGTGGTAGTGAAAATTGAGGAAGCACCTCTAAGCTCTTCAGAAGAGGATCACCCTG
**Xcl1**	NM_008510.1	AGAGAGTAGCTGTGTGAACTTACAAACCCAGCGGCTGCCAGTTCAAAAAATCAAGACCTATATCATCTGGGAGGGGGCCATGAGAGCTGTAATTTTTGTC
**Xcr1**	NM_011798.4	CTGTCTACTGCCTGTGTTGATCTCAGCACAATGGAGTTGGTTTCTAGGTGACTTCTTCTGCAAGTTCTTCAACATGATCTTCGGCATCAGCCTCTACAGC
**Ythdf2**	NM_145393.4	GGACAGTCTACTCAAAGCTCTGGATATAGTAGCAATTACGCTTATGCACCCAGCTCCTTAGGTGGAGCCATGATTGATGGACAGTCAGCTTTTGCCAATG
**Yy1**	NM_009537.3	CGGTCACCATGTGGTCCTCGGATGAAAAAAAAGATATTGACCATGAAACAGTGGTTGAAGAGCAGATCATTGGAGAGAACTCACCTCCTGATTATTCTGA
**Zap70**	NM_009539.2	TACGAGAGTCCCTACAGCGACCCTGAGGAACTCAAAGACAAGAAGCTCTTCCTGAAGCGAGAGAATCTCCTCGTGGCGGACATCGAGCTTGGCTGTGGCA
**Zbp1**	NM_021394.2	CGCCAAGGCTCTGGGAATGACGACAGCCAAAGAAGTGAACCCACTCCTGTATTCCATGAGAAATAAGCACCTTCTGAGCTATGACGGACAGACGTGGAAG
**Zfp13**	NM_011747.2	TCTGCAGACAGTCAAAGCATCGCGGCTACTGAGAACGAGGAGAAATCCCATGAGGTCCCAGGTAATGTGCAACACTGTGGAGACATGCTCTCTGGACAGG
Internal Reference Genes		
**Abcf1**	NM_013854.1	GAGGTGTCTTCCCGCCAGGCAATGTTAGAAAATGCATCTGACATTAAGTTGGAAAAGTTCAGCATCTCCGCCCACGGCAAGGAGCTATTCGTCAATGCTG
**Alas1**	NM_020559.2	ACTAGGAATATTTCTGGAACGAGCAAGTTCCACGTAGAACTGGAGCAGGCACTGGCCGACCTCCACGGCAAAGATGCGGCGCTCTTGTTCTCTTCCTGTT
**Edc3**	NM_153799.3	CTTTATAGTTGCCCTCCTCAGGTGTATAGTTGGAACTAGTGGCCTGTCTCAAACATGCCAAAAATGAGCCCAGTTCTAGATGACAGTAGTGGGATTCAGC
**Eef1g**	NM_026007.4	GGCAAGGTTCCAGCATTTGAGGGTGATGATGGATTCTGTGTGTTTGAGAGCAATGCCATTGCCTATTATGTAAGCAATGAGGAGCTGCGAGGAAGTACGC
**Eif2b4**	NM_001127355.1	AGTACTGGTCTGCTGTGAAACGTACAAGTTCTGTGAACGTGTGCAGACTGATGCCTTTGTCTCCAACGAGCTAGATGATCCCGATGATCTGCAGTGTAAG

**G6pdx**	NM_008062.2	ACATTCTAGTTCCTGGGCTTGGACCGCCATTTTGTCCTATGCTGCTGCCACTGCCACCACCAGTAAACCCAGCTACATTCCTCAAATACCAGGCATTTAA
**Gusb**	NM_010368.1	AATACGTGGTCGGAGAGCTCATCTGGAATTTCGCCGACTTCATGACGAACCAGTCACCGCTGAGAGTAATCGGAAACAAGAAGGGGATCTTCACTCGCCA
**Hdac3**	NM_010411.2	ATGAAACCTCATCGCCTGGCATTGACTCATAGCCTAGTCCTGCATTATGGTCTCTATAAGAAGATGATCGTCTTCAAGCCTTACCAGGCCTCCCAGCATG
**Hprt**	NM_013556.2	ATGACTGTAGATTTTATCAGACTGAAGAGCTACTGTAATGATCAGTCAACGGGGGACATAAAAGTTATTGGTGGAGATGATCTCTCAACTTTAACTGGAA
**Nubp1**	NM_011955.2	TGGAGATACACAGGTTGCTCTTCTGGACATCGATATCTGTGGGCCATCGATTCCCAAGATCATGGGCTTAGAAGGAGAGCAGGTTCACCAGAGCGGCTCC
**Oaz1**	NM_008753.4	CCCTGTGCCCTCTCCTGGGTTAGTCCACATGTCGTGATTGTGCAGAATAAACGCTCACTCCATTAGCGGGGTGCTTCTTCGAGCTGAATGCTGTGTTTGT
**Polr1b**	NM_009086.2	TGCCTTTCACTGAGAGTGGCATGATGCCGGACATTCTGTTTAATCCTCACGGGTTTCCCTCCCGTATGACCATAGGTATGTTAATCGAGAGCATGGCTGG
**Polr2a**	NM_009089.2	GTGAATCGTATTCTCAATGATGCTCGAGACAAAACTGGCTCCTCTGCACAGAAATCCCTCTCTGAATATAACAACTTCAAGTCTATGGTGGTGTCTGGAG
**Ppia**	NM_008907.1	CCAAGACTGAATGGCTGGATGGCAAGCATGTGGTCTTTGGGAAGGTGAAAGAAGGCATGAACATTGTGGAAGCCATGGAGCGTTTTGGGTCCAGGAATGG
**Rpl19**	NM_009078.2	GCCTCTAGTGTCCTCCGCTGCGGGAAAAAGAAGGTCTGGTTGGATCCCAATGAGACCAATGAAATCGCCAATGCCAACTCCCGTCAGCAGATCAGGAAGC
**Sap130**	NM_172965.2	TAAATCCGAAGTGCATGTGTCTATAGCCACTCCAGTTACAGTGTCCTTGGAGACCATATCCAATCAAAATGCCGAACAGCCTACCGTTGCTGTCCCTCCG
**Sdha**	NM_023281.1	CTTGCGAGCTGCATTTGGCCTTTCTGAGGCAGGGTTTAATACTGCATGCCTTACAAAGCTCTTTCCTACCCGATCACATACTGTTGCAGCACAGGGAGGT
**Sf3a3**	NM_029157.3	ACAATTTTAGAGCAGCAACGGCGCTATCATGAGGAGAAGGAACGGCTTATGGATGTTATGGCCAAAGAGATGCTTACGAAGAAGTCCACGCTTCGGGACC
**Tbp**	NM_013684.3	GTGGCGGGTATCTGCTGGCGGTTTGGCTAGGTTTCTGCGGTCGCGTCATTTTCTCCGCAGTGCCCAGCATCACTATTTCATGGTGTGTGAAGATAACCCA
**Tubb5**	NM_011655.4	ATTGGAAGTGTCTTCCCTGTATTGGTTCTCCTTTCTCGGAGAGATGGGGGTTGGGGGTGCGGCAAGGTCTTGGTCTTGGTCTCTGAACACTCCCAATTCC

Supplemental Table 2. Statistically significant, differentially expressed genes from PBMC of human AML or MDS patients comparing post-AZA treatment, responsive samples compared to matched, pre-treatment samples.

GeneID	padj	pvalue	lfcSE	stat	log2Fold Change	baseMean	Symbol	Description
5196	2.88e-16	2.09e-20	0.73	9.26	6.7220	914.99	PF4	platelet factor 4
81027	2.99e-16	4.35e-20	0.67	9.18	6.1426	954.86	TUBB1	tubulin beta 1 class VI
5473	3.79e-15	8.26e-19	0.79	8.86	7.0157	5007.51	PPBP	pro-platelet basic protein
10398	4.75e-15	1.38e-18	0.71	8.80	6.2499	225.77	MYL9	myosin light chain 9
4603	5.53e-15	2.01e-18	0.55	8.76	4.8364	403.83	MYBL1	MYB proto-oncogene like 1
4512	2.35e-14	1.02e-17	0.52	8.57	4.4742	880614.15	COX1	cytochrome c oxidase subunit I
64919	4.61e-14	2.34e-17	0.66	8.48	5.5624	301.09	BCL11B	BCL11 transcription factor B
9402	1.78e-13	1.04e-16	0.69	8.30	5.7090	491.35	GRAP2	GRB2 related adaptor protein 2
22806	1.61e-12	1.08e-15	0.56	8.02	4.5250	524.4	IKZF3	IKAROS family zinc finger 3
4514	1.61e-12	1.17e-15	0.66	8.01	5.2682	973257	COX3	cytochrome c oxidase subunit III
4579	2.73e-12	2.18e-15	0.67	7.93	5.3151	65989.37	TRNY	tRNA-Tyr
3575	4.61e-12	4.02e-15	0.81	7.85	6.3551	304.01	IL7R	interleukin 7 receptor
4511	9.34e-12	8.82e-15	0.67	7.76	5.1702	72358.19	TRNC	tRNA-Cys
57595	1.61e-11	1.64e-14	0.74	7.68	5.7026	151.43	PDZD4	PDZ domain containing 4
105376333	1.80e-11	1.96e-14	0.60	7.65	4.6184	1825.52	LOC105376333	uncharacterized LOC105376333
3934	6.47e-11	7.51e-14	0.95	7.48	7.1088	364.24	LCN2	lipocalin 2
6374	6.84e-10	8.44e-13	0.83	7.15	5.9436	88.61	CXCL5	C-X-C motif chemokine ligand 5
3932	9.55e-10	1.25e-12	0.72	7.10	5.1383	184.67	LCK	LCK proto-oncogene, Src family tyrosine kinase
83888	9.67e-10	1.35e-12	0.71	7.09	5.0282	242.84	FGFBP2	fibroblast growth factor binding protein 2
4513	9.67e-10	1.40e-12	0.65	7.08	4.5795	474435.28	COX2	cytochrome c oxidase subunit II

7049	2.13e-09	3.40e-12	0.56	6.96	3.8800	348.17	TGFBR3	transforming growth factor beta receptor 3
6352	2.13e-09	3.34e-12	0.72	6.96	5.0378	979.12	CCL5	C-C motif chemokine ligand 5
923	3.68e-09	6.15e-12	0.78	6.88	5.3526	96.99	CD6	CD6 molecule
800	3.79e-09	6.60e-12	0.91	6.87	6.2487	138.44	CALD1	caldesmon 1
7535	4.22e-09	7.65e-12	0.54	6.84	3.7253	633.74	ZAP70	zeta chain of T cell receptor associated protein kinase 70
9235	4.44e-09	8.38e-12	0.78	6.83	5.3229	271.94	IL32	interleukin 32
388228	1.55e-08	3.04e-11	0.75	6.64	4.9571	101.06	SBK1	SH3 domain binding kinase 1
5197	1.60e-08	3.24e-11	0.97	6.64	6.4052	65.81	PF4V1	platelet factor 4 variant 1
340205	1.83e-08	3.85e-11	0.93	6.61	6.1137	94.12	TREML1	triggering receptor expressed on myeloid cells like 1
9806	1.85e-08	4.04e-11	0.68	6.60	4.4550	432.84	SPOCK2	SPARC (osteonectin), cwcv and kazal like domains proteoglycan 2
399844	1.85e-08	4.17e-11	0.60	6.60	3.9782	2082.94	LINC01002	long intergenic non-protein coding RNA 1002
4519	1.87e-08	4.34e-11	0.71	6.59	4.6491	612427.62	CYTB	cytochrome b
9047	2.77e-08	6.84e-11	0.66	6.52	4.2718	170.51	SH2D2A	SH2 domain containing 2A
4540	2.77e-08	6.81e-11	0.69	6.52	4.5075	641497.04	ND5	NADH dehydrogenase subunit 5
1667	3.17e-08	8.30e-11	1.06	6.50	6.8993	704.86	DEFA1	defensin alpha 1
728358	3.17e-08	8.30e-11	1.06	6.50	6.8993	704.86	DEFA1B	defensin alpha 1B
83699	3.22e-08	8.64e-11	0.73	6.49	4.7208	173.77	SH3BGRL2	SH3 domain binding glutamate rich protein like 2
5335	3.42e-08	9.45e-11	0.62	6.48	4.0306	203.92	PLCG1	phospholipase C gamma 1
107984360	3.68e-08	1.04e-10	1.01	6.46	6.4975	67.89	LOC107984360	
4538	3.73e-08	1.08e-10	0.69	6.45	4.4463	939275.04	ND4	NADH dehydrogenase subunit 4
3820	4.08e-08	1.21e-10	0.76	6.44	4.8871	124.97	KLRB1	killer cell lectin like receptor B1
10417	5.66e-08	1.77e-10	0.59	6.38	3.7395	430.09	SPON2	spondin 2

915	5.66e-08	1.75e-10	0.93	6.38	5.9533	63.29	CD3D	CD3 delta subunit of T-cell receptor complex
10666	6.27e-08	2.00e-10	0.74	6.36	4.7147	503.28	CD226	CD226 molecule
919	1.05e-07	3.42e-10	0.72	6.28	4.5127	332.77	CD247	CD247 molecule
101928512	1.11e-07	3.71e-10	0.68	6.27	4.2679	127.67	LOC101928512	uncharacterized LOC101928512
8784	1.16e-07	4.05e-10	0.86	6.25	5.3934	129.67	TNFRSF18	TNF receptor superfamily member 18
3001	1.16e-07	4.01e-10	0.82	6.25	5.1487	272.2	GZMA	granzyme A
30009	1.16e-07	4.12e-10	0.69	6.25	4.3091	289.63	TBX21	T-box transcription factor 21
171558	1.48e-07	5.36e-10	1.04	6.21	6.4553	74.34	PTCRA	pre T cell antigen receptor alpha
219670	1.95e-07	7.20e-10	1.12	6.16	6.9115	53.56	ENKUR	enkurin, TRPC channel interacting protein
116987	2.02e-07	7.64e-10	0.78	6.15	4.8038	82.03	AGAP1	ArfGAP with GTPase domain, ankyrin repeat and PH domain 1
84433	2.21e-07	8.51e-10	0.58	6.14	3.5854	494.22	CARD11	caspase recruitment domain family member 11
3702	2.43e-07	9.52e-10	0.80	6.12	4.9201	181.27	ITK	IL2 inducible T cell kinase
7504	2.63e-07	1.05e-09	1.01	6.10	6.1531	72.04	XK	X-linked Kx blood group antigen, Kell and VPS13A binding protein
2815	3.03e-07	1.23e-09	0.98	6.08	5.9768	82.38	GP9	glycoprotein IX platelet
9848	3.25e-07	1.34e-09	0.79	6.06	4.8097	87.75	MFAP3L	microfibril associated protein 3 like
84281	3.62e-07	1.53e-09	0.75	6.04	4.5399	256.17	C2orf88	chromosome 2 open reading frame 88
916	4.20e-07	1.80e-09	0.89	6.01	5.3772	118.84	CD3E	CD3 epsilon subunit of T-cell receptor complex
8631	4.37e-07	1.90e-09	0.67	6.01	4.0193	126.18	SKAP1	src kinase associated phosphoprotein 1
3493	4.44e-07	1.97e-09	0.85	6.00	5.0916	76.01	IGHA1	immunoglobulin heavy constant alpha 1
286	6.56e-07	2.95e-09	0.87	5.93	5.1352	90.7	ANK1	ankyrin 1
55287	7.24e-07	3.31e-09	1.08	5.92	6.4090	57.91	TMEM40	transmembrane protein 40
113219467	7.37e-07	3.42e-09	0.76	5.91	4.5044	76179.7	MIR12136	microRNA 12136

321	7.39e-07	3.49e-09	0.93	5.91	5.4982	147.92	APBA2	amyloid beta precursor protein binding family A member 2
4574	7.85e-07	3.76e-09	0.77	5.89	4.5471	35610.06	TRNS1	tRNA-Ser
2781	8.06e-07	3.92e-09	0.83	5.89	4.8570	70.27	GNAZ	G protein subunit alpha z
53637	8.09e-07	3.99e-09	0.90	5.88	5.3183	170.03	S1PR5	sphingosine-1-phosphate receptor 5
3002	8.70e-07	4.36e-09	0.79	5.87	4.6264	378.29	GZMB	granzyme B
925	8.77e-07	4.57e-09	1.01	5.86	5.9427	145.77	CD8A	CD8a molecule
2017	8.77e-07	4.47e-09	0.84	5.87	4.9530	125.5	CTTN	cortactin
27087	8.77e-07	4.58e-09	0.73	5.86	4.3039	92.01	B3GAT1	beta-1,3-glucuronyltransferase 1
4563	9.39e-07	4.97e-09	0.84	5.85	4.9028	850.27	TRNG	tRNA-Gly
2039	9.40e-07	5.05e-09	0.83	5.85	4.8690	158.75	DMTN	dematin actin binding protein
940	1.01e-06	5.48e-09	1.31	5.83	7.6261	62.42	CD28	CD28 molecule
5551	1.20e-06	6.60e-09	0.64	5.80	3.7325	814.54	PRF1	perforin 1
9580	1.51e-06	8.45e-09	0.90	5.76	5.2089	57.92	SOX13	SRY-box transcription factor 13
3824	1.63e-06	9.33e-09	0.76	5.74	4.3793	701.43	KLRD1	killer cell lectin like receptor D1
4157	1.63e-06	9.32e-09	0.72	5.74	4.1180	107.67	MC1R	melanocortin 1 receptor
23531	2.10e-06	1.22e-08	0.75	5.70	4.2533	356.73	MMD	monocyte to macrophage differentiation associated
10578	2.17e-06	1.28e-08	0.77	5.69	4.3968	2002.81	GNLY	granulysin
3690	2.18e-06	1.30e-08	0.77	5.69	4.3970	947.38	ITGB3	integrin subunit beta 3
4646	2.19e-06	1.32e-08	0.78	5.68	4.4177	83.08	MYO6	myosin VI
4576	2.74e-06	1.67e-08	0.80	5.64	4.5201	2420	TRNT	tRNA-Thr
9651	3.05e-06	1.88e-08	0.68	5.62	3.8051	181.4	PLCH2	phospholipase C eta 2
29094	3.51e-06	2.19e-08	0.84	5.60	4.6699	138.21	LGALSL	galectin like

113218488	3.99e-06	2.52e-08	0.79	5.57	4.4242	1190.1	MIR10396B	microRNA 10396b
917	4.20e-06	2.68e-08	1.26	5.56	7.0159	43.66	CD3G	CD3 gamma subunit of T-cell receptor complex
1731	4.28e-06	2.77e-08	0.62	5.56	3.4521	165.85	SEPTIN1	septin 1
100133331	4.54e-06	2.97e-08	0.59	5.54	3.2556	1856.97	LOC100133331	replaced by ID 100288069
107987001	5.10e-06	3.37e-08	0.75	5.52	4.1566	123.26	FAM27E4	family with sequence similarity 27 member E4
105377384	5.24e-06	3.51e-08	1.78	5.51	9.8185	36.96	LOC105377384	uncharacterized LOC105377384
22885	5.24e-06	3.54e-08	1.02	5.51	5.6055	48.88	ABLIM3	actin binding LIM protein family member 3
2812	5.95e-06	4.06e-08	0.99	5.49	5.4339	165.72	GP1BB	glycoprotein Ib platelet subunit beta
4818	7.21e-06	4.97e-08	0.70	5.45	3.8226	662.86	NKG7	natural killer cell granule protein 7
22981	7.48e-06	5.21e-08	0.98	5.44	5.3075	64.59	NINL	ninein like
28526	8.20e-06	5.78e-08	0.83	5.43	4.4807	165.56	TRDC	T cell receptor delta constant
116173	8.31e-06	5.91e-08	1.19	5.42	6.4650	50.94	CMTM5	CKLF like MARVEL transmembrane domain containing 5
4555	9.49e-06	6.82e-08	0.79	5.40	4.2726	353.74	TRND	tRNA-Asp
51176	9.82e-06	7.13e-08	1.02	5.39	5.4696	100.03	LEF1	lymphoid enhancer binding factor 1
3560	9.90e-06	7.26e-08	0.81	5.38	4.3773	842.89	IL2RB	interleukin 2 receptor subunit beta
100506736	1.00e-05	7.41e-08	0.64	5.38	3.4365	213.79	SLFN12L	schlafen family member 12 like
139105	1.24e-05	9.26e-08	1.42	5.34	7.5958	41.53	BEND2	BEN domain containing 2
100507747	1.41e-05	1.06e-07	0.72	5.32	3.8251	387.43	C13orf46	chromosome 13 open reading frame 46
57121	1.57e-05	1.19e-07	0.90	5.29	4.7810	57.28	LPAR5	lysophosphatidic acid receptor 5
10225	1.69e-05	1.30e-07	0.73	5.28	3.8536	249.02	CD96	CD96 molecule
4318	1.83e-05	1.44e-07	1.00	5.26	5.2383	60.45	MMP9	matrix metallopeptidase 9
128611	1.83e-05	1.44e-07	0.70	5.26	3.6576	276.18	ZNF831	zinc finger protein 831

152789	1.94e-05	1.53e-07	1.01	5.25	5.3183	46.41	JAKMIP1	janus kinase and microtubule interacting protein 1
389813	2.04e-05	1.63e-07	0.79	5.24	4.1191	80.25	AJM1	apical junction component 1 homolog
100132062	2.17e-05	1.75e-07	0.59	5.22	3.0796	1831.83	LOC100132062	uncharacterized LOC100132062
4068	2.35e-05	1.91e-07	0.98	5.21	5.0978	53.69	SH2D1A	SH2 domain containing 1A
10316	2.41e-05	1.99e-07	0.71	5.20	3.7122	101.1	NMUR1	neuromedin U receptor 1
2791	2.41e-05	2.01e-07	0.86	5.20	4.4424	208.83	GNG11	G protein subunit gamma 11
342618	2.41e-05	2.01e-07	0.91	5.20	4.7030	103.18	SLFN14	schlafen family member 14
5159	2.99e-05	2.52e-07	0.76	5.16	3.9102	102.47	PDGFRB	platelet derived growth factor receptor beta
23762	3.02e-05	2.57e-07	0.76	5.15	3.8987	79.18	OSBP2	oxysterol binding protein 2
107987206	3.11e-05	2.68e-07	0.83	5.14	4.2491	3084253.92	LOC107987206	replaced by ID 6029
6029	3.11e-05	2.68e-07	0.83	5.14	4.2491	3084253.42	RN7SL1	RNA component of signal recognition particle 7SL1
4541	3.16e-05	2.75e-07	0.84	5.14	4.3254	185894.86	ND6	NADH dehydrogenase subunit 6
2999	3.16e-05	2.78e-07	0.85	5.14	4.3693	128.86	GZMH	granzyme H
1901	4.07e-05	3.61e-07	0.92	5.09	4.6579	78.89	S1PR1	sphingosine-1-phosphate receptor 1
401124	4.53e-05	4.04e-07	0.89	5.07	4.4885	82.71	DTHD1	death domain containing 1
101927854	5.07e-05	4.56e-07	1.89	5.04	9.5540	31.05	THRB-AS2	THRB antisense RNA 2
105378179	5.99e-05	5.48e-07	0.84	5.01	4.2007	4041673.74	LOC105378179	uncharacterized LOC105378179
378706	5.99e-05	5.48e-07	0.84	5.01	4.2008	4041641.81	RN7SL2	RNA component of signal recognition particle 7SL2
387882	8.09e-05	7.46e-07	1.01	4.95	5.0084	135.94	C12orf75	chromosome 12 open reading frame 75
1236	8.44e-05	7.85e-07	1.26	4.94	6.2013	33.87	CCR7	C-C motif chemokine receptor 7
4508	8.78e-05	8.22e-07	0.85	4.93	4.2116	605386.23	ATP6	ATP synthase F0 subunit 6
6775	9.32e-05	8.80e-07	0.67	4.92	3.2960	190.45	STAT4	signal transducer and activator of transcription 4

4753	9.57e-05	9.10e-07	1.74	4.91	8.5485	40.75	NELL2	neural EGFL like 2
2625	1.03e-04	9.87e-07	0.77	4.89	3.7669	137.39	GATA3	GATA binding protein 3
9214	1.24e-04	1.20e-06	0.82	4.86	3.9582	90.93	FCMR	Fc mu receptor
84886	1.27e-04	1.23e-06	0.75	4.85	3.6446	119.31	C1orf198	chromosome 1 open reading frame 198
347404	1.39e-04	1.37e-06	0.99	4.83	4.8007	48.83	LANCL3	LanC like family member 3
8654	1.45e-04	1.43e-06	0.70	4.82	3.3488	165.13	PDE5A	phosphodiesterase 5A
4568	1.49e-04	1.48e-06	0.94	4.81	4.5051	868.52	TRNL2	tRNA-Leu
2113	1.49e-04	1.50e-06	0.70	4.81	3.3880	955.33	ETS1	ETS proto-oncogene 1, transcription factor
109864279	1.52e-04	1.53e-06	0.83	4.81	3.9706	538366.19	RNA45SN2	RNA, 45S pre-ribosomal N2
114804	1.62e-04	1.64e-06	1.20	4.79	5.7492	36.05	RNF157	ring finger protein 157
100131997	1.67e-04	1.71e-06	0.90	4.78	4.2977	114.65	FAM27E3	family with sequence similarity 27 member E3
2811	1.67e-04	1.72e-06	0.74	4.78	3.5548	200.93	GP1BA	glycoprotein Ib platelet subunit alpha
100008589	2.15e-04	2.23e-06	0.58	4.73	2.7227	1773.16	RNA28SN5	RNA, 28S ribosomal N5
5727	2.75e-04	2.88e-06	0.87	4.68	4.0578	63.79	PTCH1	patched 1
914	3.16e-04	3.33e-06	0.98	4.65	4.5406	154.04	CD2	CD2 molecule
4509	3.46e-04	3.67e-06	0.81	4.63	3.7444	70464.84	ATP8	ATP synthase F0 subunit 8
100233209	3.64e-04	3.88e-06	0.94	4.62	4.3254	57.35	PCED1B-AS1	PCED1B antisense RNA 1
8555	3.67e-04	3.95e-06	1.16	4.61	5.3280	32.12	CDC14B	cell division cycle 14B
109864272	3.78e-04	4.09e-06	0.58	4.61	2.6636	1416.82	RNA28SN4	RNA, 28S ribosomal N4
109864282	4.00e-04	4.36e-06	0.58	4.59	2.6780	1379.87	RNA28SN2	RNA, 28S ribosomal N2
1292	4.05e-04	4.43e-06	1.01	4.59	4.6293	484.55	COL6A2	collagen type VI alpha 2 chain
80739	4.31e-04	4.75e-06	0.91	4.58	4.1749	571.27	MPIG6B	megakaryocyte and platelet inhibitory receptor G6b

105379857	4.54e-04	5.04e-06	0.84	4.56	3.8449	58.53	LOC105379857	replaced by ID 642819
4638	4.54e-04	5.08e-06	0.91	4.56	4.1321	130.67	MYLK	myosin light chain kinase
6678	4.66e-04	5.24e-06	0.82	4.55	3.7413	360.13	SPARC	secreted protein acidic and cysteine rich
8436	5.11e-04	5.82e-06	0.90	4.53	4.0978	545.74	CAVIN2	caveolae associated protein 2
378707	5.11e-04	5.86e-06	0.76	4.53	3.4260	338691.09	RN7SL3	RNA component of signal recognition particle 7SL3
105372446	5.11e-04	5.86e-06	1.06	4.53	4.7926	39.8	LIM2-AS1	LIM2 and SIGLEC10 antisense RNA 1
106632264	5.16e-04	5.95e-06	0.59	4.53	2.6802	1688.58	RNA28SN1	RNA, 28S ribosomal N1
5730	5.73e-04	6.66e-06	0.85	4.50	3.8484	74.51	PTGDS	prostaglandin D2 synthase
348378	6.02e-04	7.04e-06	1.00	4.49	4.5061	49.36	SHISAL2A	shisa like 2A
81606	6.29e-04	7.40e-06	0.74	4.48	3.3265	112.26	LBH	LBH regulator of WNT signaling pathway
79616	6.35e-04	7.52e-06	1.03	4.48	4.6155	38.45	CCNJL	cyclin J like
4535	6.37e-04	7.58e-06	0.86	4.48	3.8383	713934.18	ND1	NADH dehydrogenase subunit 1
5577	6.62e-04	7.93e-06	0.77	4.47	3.4346	474.72	PRKAR2B	protein kinase cAMP-dependent type II regulatory subunit beta
4564	6.70e-04	8.08e-06	0.93	4.46	4.1357	1883.31	TRNH	tRNA-His
107986461	6.71e-04	8.13e-06	1.32	4.46	5.9042	26.15	LOC107986461	uncharacterized LOC107986461
4566	7.44e-04	9.07e-06	0.89	4.44	3.9404	1543.83	TRNK	tRNA-Lys
9886	7.46e-04	9.16e-06	0.78	4.44	3.4675	92.92	RHOBTB1	Rho related BTB domain containing 1
728262	8.18e-04	1.01e-05	0.59	4.41	2.6184	2010.11	FAM157A	family with sequence similarity 157 member A
4550	8.18e-04	1.02e-05	0.90	4.41	3.9566	262.81	RNR2	l-rRNA
8530	8.88e-04	1.11e-05	0.71	4.39	3.1021	207.99	CST7	cystatin F
6095	9.03e-04	1.13e-05	0.75	4.39	3.3120	244.43	RORA	RAR related orphan receptor A
85379	9.15e-04	1.16e-05	1.22	4.39	5.3521	27.68	KIAA1671	KIAA1671

54843	9.23e-04	1.17e-05	0.71	4.38	3.1244	141.13	SYTL2	synaptotagmin like 2
28951	9.79e-04	1.25e-05	0.70	4.37	3.0415	101.4	TRIB2	tribbles pseudokinase 2
3003	1.03e-03	1.33e-05	1.34	4.36	5.8517	76.63	GZMK	granzyme K
100500862	1.14e-03	1.47e-05	0.93	4.33	4.0309	11787.95	MIR3648-1	microRNA 3648-1
103504731	1.15e-03	1.49e-05	0.93	4.33	4.0231	23318.02	MIR3648-2	microRNA 3648-2
100289124	1.25e-03	1.64e-05	1.10	4.31	4.7453	54.17	FAM27E2	family with sequence similarity 27 member E2
50852	1.28e-03	1.68e-05	1.82	4.30	7.8346	25.64	TRAT1	T cell receptor associated transmembrane adaptor 1
149628	1.30e-03	1.71e-05	0.74	4.30	3.1719	148.48	PYHIN1	pyrin and HIN domain family member 1
154075	1.30e-03	1.76e-05	0.84	4.29	3.6147	206.39	SAMD3	sterile alpha motif domain containing 3
23224	1.30e-03	1.75e-05	0.68	4.29	2.8986	1317.56	SYNE2	spectrin repeat containing nuclear envelope protein 2
9437	1.30e-03	1.74e-05	0.91	4.30	3.9240	76.34	NCR1	natural cytotoxicity triggering receptor 1
113218501	1.30e-03	1.76e-05	1.04	4.29	4.4554	268.18	MIR10396A	microRNA 10396a
4536	1.36e-03	1.85e-05	0.89	4.28	3.8011	734832.3	ND2	NADH dehydrogenase subunit 2
343413	1.37e-03	1.87e-05	0.81	4.28	3.4488	73.61	FCRL6	Fc receptor like 6
6403	1.46e-03	2.00e-05	0.88	4.27	3.7668	135.52	SELP	selectin P
9254	1.69e-03	2.34e-05	0.88	4.23	3.7231	57.73	CACNA2D2	calcium voltage-gated channel auxiliary subunit alpha2delta 2
8277	1.69e-03	2.34e-05	1.21	4.23	5.1177	51.12	TKTL1	transketolase like 1
6043	1.81e-03	2.53e-05	0.94	-4.21	-3.9531	122.45	SNORA63	small nucleolar RNA, H/ACA box 63
9953	1.82e-03	2.55e-05	0.84	4.21	3.5489	94.43	HS3ST3B1	heparan sulfate-glucosamine 3-sulfotransferase 3B1
2920	1.85e-03	2.60e-05	0.64	-4.21	-2.6869	371.47	CXCL2	C-X-C motif chemokine ligand 2
28639	1.86e-03	2.63e-05	0.95	4.20	4.0018	55.35	TRBC1	T cell receptor beta constant 1
8807	1.88e-03	2.67e-05	0.69	4.20	2.9035	136.42	IL18RAP	interleukin 18 receptor accessory protein

5583	1.88e-03	2.69e-05	0.60	4.20	2.5272	810.23	PRKCH	protein kinase C eta
100128731	1.91e-03	2.75e-05	0.59	4.19	2.4621	589.79	OST4	oligosaccharyltransferase complex subunit 4, non-catalytic
100130231	1.95e-03	2.81e-05	1.03	4.19	4.3111	108.49	LINC00861	long intergenic non-protein coding RNA 861
666	2.02e-03	2.93e-05	0.94	4.18	3.9099	43.61	BOK	BCL2 family apoptosis regulator BOK
143872	2.09e-03	3.05e-05	1.18	4.17	4.9206	31.45	ARHGAP42	Rho GTPase activating protein 42
1793	2.14e-03	3.14e-05	0.87	-4.16	-3.5991	192.46	DOCK1	dedicator of cytokinesis 1
84131	2.33e-03	3.43e-05	0.61	4.14	2.5401	289.46	CEP78	centrosomal protein 78
259215	2.48e-03	3.68e-05	1.24	4.13	5.1340	28.73	LY6G6F	lymphocyte antigen 6 family member G6F
51348	2.48e-03	3.69e-05	0.95	4.13	3.8998	150	KLRF1	killer cell lectin like receptor F1
81563	2.59e-03	3.87e-05	0.72	4.12	2.9448	137.04	C1orf21	chromosome 1 open reading frame 21
105377806	2.68e-03	4.03e-05	0.85	4.11	3.4713	109.15	LOC105377806	
4050	2.77e-03	4.18e-05	0.66	4.10	2.6877	138.21	LTB	lymphotoxin beta
81794	2.98e-03	4.53e-05	0.65	4.08	2.6647	253.96	ADAMTS10	ADAM metallopeptidase with thrombospondin type 1 motif 10
339541	3.11e-03	4.75e-05	1.00	-4.07	-4.0516	148.54	ARMH1	armadillo like helical domain containing 1
58486	3.25e-03	4.97e-05	0.72	-4.06	-2.9294	120.56	ZBED5	zinc finger BED-type containing 5
3693	3.59e-03	5.52e-05	0.83	4.03	3.3455	79.38	ITGB5	integrin subunit beta 5
8787	3.68e-03	5.69e-05	0.93	4.03	3.7281	42.63	RGS9	regulator of G protein signaling 9
10158	3.90e-03	6.09e-05	1.88	4.01	7.5241	20.9	PDZK1IP1	PDZK1 interacting protein 1
57732	3.90e-03	6.07e-05	0.68	4.01	2.7084	117.73	ZFYVE28	zinc finger FYVE-type containing 28
22914	3.90e-03	6.12e-05	0.94	4.01	3.7704	103.95	KLRK1	killer cell lectin like receptor K1
54796	3.98e-03	6.27e-05	1.01	4.00	4.0214	46.94	BNC2	basonuclin 2
101929531	4.34e-03	6.87e-05	1.33	3.98	5.2740	22.25	LINC01871	long intergenic non-protein coding RNA 1871

79993	4.55e-03	7.23e-05	0.89	3.97	3.5273	135.06	ELOVL7	ELOVL fatty acid elongase 7
93010	4.56e-03	7.29e-05	0.98	3.97	3.9048	37.06	B3GNT7	UDP-GlcNAc:betaGal beta-1,3-N-acetylglucosaminyltransferase 7
864	4.57e-03	7.32e-05	0.60	3.97	2.3704	1024.65	RUNX3	RUNX family transcription factor 3
4900	4.76e-03	7.67e-05	0.68	3.95	2.6853	1430.52	NRGN	neurogranin
109910382	5.10e-03	8.25e-05	0.55	3.94	2.1542	1042.18	RNA28SN3	RNA, 28S ribosomal N3
105379807	5.44e-03	8.87e-05	1.17	3.92	4.5978	38.71	LOC105379807	uncharacterized LOC105379807
92591	5.44e-03	8.88e-05	0.87	3.92	3.4114	171.51	ASB16	ankyrin repeat and SOCS box containing 16
124602	5.65e-03	9.27e-05	1.11	3.91	4.3402	44.07	KIF19	kinesin family member 19
9289	5.80e-03	9.56e-05	0.68	3.90	2.6365	465.08	ADGRG1	adhesion G protein-coupled receptor G1
117157	5.92e-03	9.81e-05	0.99	3.90	3.8584	117.34	SH2D1B	SH2 domain containing 1B
54758	5.98e-03	9.95e-05	0.57	3.89	2.2291	299.45	KLHDC4	kelch domain containing 4
760	6.08e-03	1.01e-04	0.82	3.89	3.1916	123.83	CA2	carbonic anhydrase 2
3823	6.16e-03	1.03e-04	0.92	3.88	3.5710	59.19	KLRC3	killer cell lectin like receptor C3
5874	6.32e-03	1.06e-04	0.87	3.88	3.3555	240.25	RAB27B	RAB27B, member RAS oncogene family
60509	6.62e-03	1.12e-04	0.61	3.86	2.3535	382.95	AGBL5	AGBL carboxypeptidase 5
4573	6.62e-03	1.12e-04	0.95	3.86	3.6809	306.24	TRNR	tRNA-Arg
2274	6.69e-03	1.14e-04	0.88	3.86	3.4101	48.27	FHL2	four and a half LIM domains 2
11098	6.80e-03	1.16e-04	0.91	3.85	3.5156	51.9	PRSS23	serine protease 23
4569	6.97e-03	1.20e-04	0.89	3.85	3.4226	682.49	TRNM	tRNA-Met
4537	7.23e-03	1.25e-04	0.92	3.84	3.5099	157586.17	ND3	NADH dehydrogenase subunit 3
4539	7.81e-03	1.35e-04	0.93	3.82	3.5386	139355.36	ND4L	NADH dehydrogenase subunit 4L
23043	8.03e-03	1.40e-04	0.56	3.81	2.1283	380.44	TNIK	TRAF2 and NCK interacting kinase

10125	8.03e-03	1.40e-04	0.71	3.81	2.6914	163.54	RASGRP1	RAS guanyl releasing protein 1
26030	8.07e-03	1.42e-04	0.53	3.81	2.0257	470.15	PLEKHG3	pleckstrin homology and RhoGEF domain containing G3
6932	8.10e-03	1.43e-04	0.82	3.80	3.1000	387.11	TCF7	transcription factor 7
9495	8.39e-03	1.49e-04	0.93	3.79	3.5344	48.48	AKAP5	A-kinase anchoring protein 5
107987026	8.62e-03	1.53e-04	0.93	3.79	3.5297	95.78	LOC107987026	uncharacterized LOC107987026
109864271	8.86e-03	1.58e-04	0.90	3.78	3.4098	426294.63	RNA45SN4	RNA, 45S pre-ribosomal N4
10123	9.12e-03	1.64e-04	0.58	3.77	2.1911	1100.53	ARL4C	ADP ribosylation factor like GTPase 4C
100528032	9.52e-03	1.71e-04	0.93	3.76	3.5015	120.66	KLRC4-KLRK1	KLRC4-KLRK1 readthrough
820	1.03e-02	1.87e-04	1.21	3.74	4.5029	32.12	CAMP	cathelicidin antimicrobial peptide
83988	1.03e-02	1.87e-04	0.79	3.74	2.9563	63.94	NCALD	neurocalcin delta
11278	1.07e-02	1.95e-04	0.75	3.73	2.8096	242.64	KLF12	KLF transcription factor 12
57211	1.08e-02	1.97e-04	1.88	-3.72	-7.0025	68.04	ADGRG6	adhesion G protein-coupled receptor G6
387509	1.11e-02	2.04e-04	1.05	3.71	3.9001	32.45	GPR153	G protein-coupled receptor 153
4575	1.11e-02	2.04e-04	0.98	3.71	3.6310	1251.35	TRNS2	tRNA-Ser
55930	1.12e-02	2.07e-04	1.82	-3.71	-6.7664	65.57	MYO5C	myosin VC
102724646	1.13e-02	2.10e-04	0.71	3.71	2.6310	165.84	LOC102724646	uncharacterized LOC102724646
129049	1.13e-02	2.12e-04	1.47	3.70	5.4433	22.97	SGSM1	small G protein signaling modulator 1
440823	1.25e-02	2.34e-04	0.72	3.68	2.6367	193.92	MIAT	myocardial infarction associated transcript
9124	1.27e-02	2.39e-04	0.80	3.67	2.9344	198.16	PDLIM1	PDZ and LIM domain 1
3674	1.27e-02	2.39e-04	0.85	3.67	3.1086	365.67	ITGA2B	integrin subunit alpha 2b
84879	1.31e-02	2.49e-04	1.64	-3.66	-5.9959	37.54	MFSD2A	MFSD2 lysolipid transporter A, lysophospholipid
23345	1.31e-02	2.49e-04	0.53	3.66	1.9435	2229.53	SYNE1	spectrin repeat containing nuclear envelope protein 1

105376626	1.32e-02	2.52e-04	1.92	-3.66	-7.0378	27.27	LOC105376626	uncharacterized LOC105376626
54438	1.33e-02	2.55e-04	0.56	3.66	2.0469	310.53	GFOD1	glucose-fructose oxidoreductase domain containing 1
2696	1.35e-02	2.59e-04	1.16	3.65	4.2418	26.77	GIPR	gastric inhibitory polypeptide receptor
55655	1.35e-02	2.61e-04	1.06	3.65	3.8808	45.33	NLRP2	NLR family pyrin domain containing 2
54855	1.41e-02	2.74e-04	1.11	3.64	4.0208	282.75	TENT5C	terminal nucleotidyltransferase 5C
161882	1.43e-02	2.79e-04	0.65	3.63	2.3771	129.01	ZFPM1	zinc finger protein, FOG family member 1
26051	1.52e-02	2.97e-04	0.60	3.62	2.1631	192.26	PPP1R16B	protein phosphatase 1 regulatory subunit 16B
112268284	1.52e-02	2.97e-04	0.73	3.62	2.6257	547.02	LOC112268284	
28638	1.55e-02	3.06e-04	0.90	3.61	3.2520	98.06	TRBC2	T cell receptor beta constant 2
3039	1.61e-02	3.18e-04	2.13	3.60	7.6657	3003.76	HBA1	hemoglobin subunit alpha 1
8542	1.62e-02	3.21e-04	0.76	3.60	2.7336	760.21	APOL1	apolipoprotein L1
122416	1.75e-02	3.49e-04	0.76	3.58	2.7136	105.46	ANKRD9	ankyrin repeat domain 9
23336	1.75e-02	3.50e-04	1.06	3.57	3.7834	31.75	SYNM	synemin
3004	1.75e-02	3.49e-04	1.19	3.58	4.2615	31.8	GZMM	granzyme M
4571	1.80e-02	3.62e-04	0.89	3.57	3.1609	5876.29	TRNP	tRNA-Pro
284	1.84e-02	3.71e-04	1.34	-3.56	-4.7648	124.32	ANGPT1	angiopoietin 1
106633800	1.85e-02	3.75e-04	0.80	-3.56	-2.8334	164.61	SNORD133	small nucleolar RNA, C/D box 133
59352	1.86e-02	3.78e-04	1.10	3.55	3.9145	51.9	LGR6	leucine rich repeat containing G protein-coupled receptor 6
7293	1.89e-02	3.85e-04	0.99	3.55	3.5203	57.36	TNFRSF4	TNF receptor superfamily member 4
9053	1.93e-02	3.96e-04	1.52	-3.54	-5.3839	75.24	MAP7	microtubule associated protein 7
3494	1.93e-02	3.96e-04	1.22	3.54	4.3330	30.15	IGHA2	immunoglobulin heavy constant alpha 2 (A2m marker)
3738	1.96e-02	4.03e-04	0.59	3.54	2.0960	401.84	KCNA3	potassium voltage-gated channel subfamily A member 3

494470	1.99e-02	4.12e-04	1.00	3.53	3.5138	62.46	RNF165	ring finger protein 165
139065	1.99e-02	4.13e-04	0.94	-3.53	-3.3180	76.99	SLITRK4	SLIT and NTRK like family member 4
202020	2.09e-02	4.35e-04	1.92	-3.52	-6.7558	22.42	TAPT1-AS1	TAPT1 antisense RNA 1 (head to head)
5521	2.10e-02	4.41e-04	0.94	3.51	3.3171	51.66	PPP2R2B	protein phosphatase 2 regulatory subunit Bbeta
256691	2.10e-02	4.39e-04	1.45	-3.52	-5.0834	113.64	MAMDC2	MAM domain containing 2
23348	2.10e-02	4.41e-04	1.01	3.51	3.5339	53.89	DOCK9	dedicator of cytokinesis 9
103021164	2.10e-02	4.43e-04	2.02	3.51	7.0951	15.17	CASC21	cancer susceptibility 21
84002	2.10e-02	4.45e-04	0.98	-3.51	-3.4341	150.14	B3GNT5	UDP-GlcNAc:betaGal beta-1,3-N-acetylglucosaminyltransferase 5
6622	2.15e-02	4.57e-04	0.83	3.50	2.9029	160.64	SNCA	synuclein alpha
2322	2.17e-02	4.65e-04	0.82	-3.50	-2.8844	205.22	FLT3	fms related receptor tyrosine kinase 3
3040	2.17e-02	4.62e-04	2.22	3.50	7.7566	3930.13	HBA2	hemoglobin subunit alpha 2
6915	2.17e-02	4.65e-04	1.28	3.50	4.4765	34.89	TBXA2R	thromboxane A2 receptor
100037417	2.35e-02	5.07e-04	0.75	3.48	2.6199	275.03	DDTL	D-dopachrome tautomerase like
5729	2.41e-02	5.22e-04	0.91	3.47	3.1667	65.68	PTGDR	prostaglandin D2 receptor
8809	2.53e-02	5.48e-04	0.98	3.46	3.3904	73.66	IL18R1	interleukin 18 receptor 1
10178	2.64e-02	5.75e-04	0.96	3.44	3.2996	49.23	TENM1	teneurin transmembrane protein 1
105369402	2.65e-02	5.79e-04	1.94	-3.44	-6.6822	21.29	LOC105369402	uncharacterized LOC105369402
652966	2.68e-02	5.88e-04	0.93	-3.44	-3.2043	118.06	SNORD10	small nucleolar RNA, C/D box 10
10826	2.69e-02	5.92e-04	0.69	3.44	2.3688	210.39	FAXDC2	fatty acid hydroxylase domain containing 2
6080	2.84e-02	6.27e-04	0.74	-3.42	-2.5439	587.68	SNORA73A	small nucleolar RNA, H/ACA box 73A
54541	2.89e-02	6.40e-04	0.64	3.41	2.1916	140.83	DDIT4	DNA damage inducible transcript 4
8320	2.95e-02	6.55e-04	1.12	3.41	3.8075	35.91	EOMES	eomesodermin

8425	2.95e-02	6.57e-04	0.67	3.41	2.2964	323.27	LTBP4	latent transforming growth factor beta binding protein 4
8510	2.96e-02	6.64e-04	0.81	3.40	2.7449	81.45	MMP23B	matrix metallopeptidase 23B
55607	2.96e-02	6.62e-04	1.01	3.40	3.4504	32.61	PPP1R9A	protein phosphatase 1 regulatory subunit 9A
4684	3.15e-02	7.09e-04	0.87	3.39	2.9587	221.2	NCAM1	neural cell adhesion molecule 1
158038	3.16e-02	7.13e-04	1.10	3.38	3.7310	27.66	LINGO2	leucine rich repeat and Ig domain containing 2
54797	3.20e-02	7.24e-04	0.80	3.38	2.7041	293.08	MED18	mediator complex subunit 18
54910	3.20e-02	7.27e-04	0.72	3.38	2.4343	114.63	SEMA4C	semaphorin 4C
1668	3.59e-02	8.19e-04	2.06	3.35	6.8952	845.14	DEFA3	defensin alpha 3
100526833	3.65e-02	8.36e-04	1.09	3.34	3.6273	233.04	SEPT5-GP1BB	SEPT5-GP1BB readthrough
4570	3.65e-02	8.37e-04	0.81	3.34	2.6991	4503.41	TRNN	tRNA-Asn
64407	3.70e-02	8.55e-04	0.62	3.33	2.0712	910.06	RGS18	regulator of G protein signaling 18
1116	3.70e-02	8.57e-04	1.17	3.33	3.9016	77.95	CHI3L1	chitinase 3 like 1
4602	3.70e-02	8.54e-04	1.19	-3.33	-3.9641	309.63	MYB	MYB proto-oncogene, transcription factor
671	3.79e-02	8.80e-04	0.90	3.33	3.0079	407.29	BPI	bactericidal permeability increasing protein
1521	3.79e-02	8.84e-04	0.74	3.33	2.4454	352.02	CTSW	cathepsin W
5243	3.95e-02	9.24e-04	0.88	3.31	2.9062	82.04	ABCB1	ATP binding cassette subfamily B member 1
8690	3.99e-02	9.36e-04	0.90	-3.31	-2.9668	59.6	JRKL	JRK like
256380	4.00e-02	9.40e-04	0.90	3.31	2.9675	105.59	SCML4	Scm polycomb group protein like 4
6232	4.03e-02	9.53e-04	0.59	3.30	1.9478	2438.21	RPS27	ribosomal protein S27
26053	4.03e-02	9.51e-04	0.71	3.30	2.3556	184.35	AUTS2	activator of transcription and developmental regulator AUTS2
23365	4.07e-02	9.67e-04	0.72	3.30	2.3616	246.27	ARHGEF12	Rho guanine nucleotide exchange factor 12
57613	4.09e-02	9.74e-04	1.97	-3.30	-6.4960	18.72	FAM234B	family with sequence similarity 234 member B

652965	4.14e-02	9.89e-04	0.76	-3.29	-2.5110	441.42	SNORA48	small nucleolar RNA, H/ACA box 48
102724364	4.34e-02	1.04e-03	0.60	3.28	1.9821	212.36	SEC22B4P	SEC22 homolog B4, pseudogene
4145	4.40e-02	1.06e-03	0.79	3.28	2.5723	216.52	MATK	megakaryocyte-associated tyrosine kinase
55759	4.43e-02	1.07e-03	0.91	-3.27	-2.9844	63.19	WDR12	WD repeat domain 12
60312	4.52e-02	1.09e-03	1.08	3.27	3.5314	46.95	AFAP1	actin filament associated protein 1
84628	4.63e-02	1.12e-03	0.69	3.26	2.2385	142.89	NTNG2	netrin G2
4549	4.67e-02	1.14e-03	1.11	3.25	3.6065	148.56	RNR1	s-rRNA
100861532	4.68e-02	1.14e-03	0.94	3.25	3.0486	269159.77	RNA45SN5	RNA, 45S pre-ribosomal N5
105379282	4.87e-02	1.20e-03	0.80	3.24	2.5973	156.8	LOC105379282	
4092	4.87e-02	1.19e-03	0.73	3.24	2.3625	169.55	SMAD7	SMAD family member 7
79037	4.95e-02	1.22e-03	0.82	3.23	2.6512	75.01	PVRIG	PVR related immunoglobulin domain containing
144203	4.96e-02	1.23e-03	1.14	3.23	3.6825	26.84	OVOS2	alpha-2-macroglobulin like 1 pseudogene

Supplemental Table 3. Complete Annotations Lists with Accompanying P-values from Gene Ontology Analysis of Genes Differentially Expressed in AZA-treated Compared to Vehicle-treated Mouse Blood.

Gene Ontology Cellular Compartment	p-value	Gene Ontology Molecular Function	p-value	Gene Ontology Biological Process	p-value	PANTHER Pathway	p-value
alphav-beta3 integrin-PKCalpha complex	4.58E-04	fibrinogen binding	6.29E-07	activation of membrane attack complex	2.29E-04	Blood coagulation (P00011)	1.16E-02
plasma membrane	2.34E-02	protein-containing complex binding	4.75E-02	complement activation	1.28E-02	p53 pathway (P00059)	1.93E-02
cell periphery	3.14E-02	collagen V binding	1.14E-03	positive regulation of response to stimulus	1.14E-02	Integrin signalling pathway (P00034)	4.25E-02
alpha9-beta1 integrin-ADAM8 complex	4.58E-04	collagen binding	1.73E-02	positive regulation of biological process	2.20E-03		
integrin alphaIIb-beta3 complex	6.87E-04	C-X3-C chemokine binding	1.14E-03	regulation of immune system process	4.34E-03		
integrin complex	7.53E-03	chemokine binding	2.35E-05	positive regulation of immune system process	1.20E-03		
plasma membrane signaling receptor complex	4.45E-02	cytokine binding	2.72E-06	positive regulation of adenylate cyclase-inhibiting opioid receptor signaling pathway	2.29E-04		
protein complex involved in cell adhesion	1.34E-02	protein binding	1.69E-02	regulation of adenylate cyclase-inhibiting opioid receptor signaling pathway	2.29E-04		
alphav-beta3 integrin-HMGB1 complex	6.87E-04	neuregulin binding	1.37E-03	regulation of opioid receptor signaling pathway	9.16E-04		
integrin alphav-beta3 complex	6.87E-04	growth factor binding	4.38E-04	regulation of G protein-coupled receptor signaling pathway	3.54E-02		
integrin alpha9-beta1 complex	6.87E-04	vascular endothelial growth factor receptor 2 binding	1.83E-03	positive regulation of opioid receptor signaling pathway	4.58E-04		
alphav-beta3 integrin-IGF-1-IGF1R complex	9.16E-04	vascular endothelial growth factor receptor binding	3.66E-03	positive regulation of G protein-coupled receptor signaling pathway	9.81E-03		
fibrinogen complex	1.37E-03	growth factor receptor binding	3.43E-02	positive regulation of cellular process	2.38E-02		
extracellular space	5.07E-03	complement component C3b binding	2.06E-03	angioblast cell migration	2.29E-04		
extracellular region	1.31E-02	opsonin binding	5.03E-03	cell migration	8.62E-04		
symbiont cell surface	1.60E-03	complement binding	6.40E-03	cell motility	1.70E-03		
other organism part	1.83E-03	insulin-like growth factor I binding	2.97E-03	blood vessel morphogenesis	9.78E-05		
serine-type endopeptidase complex	3.89E-03	insulin-like growth factor binding	4.12E-03	tube morphogenesis	4.24E-04		
serine-type peptidase complex	4.34E-03	fibroblast growth factor binding	1.36E-05	anatomical structure morphogenesis	6.97E-04		
peptidase complex	2.90E-02	fibronectin binding	2.63E-05	developmental process	1.63E-03		
endopeptidase complex	2.22E-02	protein disulfide isomerase activity	4.12E-03	anatomical structure development	1.10E-03		
glycinergic synapse	4.12E-03	intramolecular oxidoreductase activity, transposing S-S bonds	4.12E-03	tube development	1.02E-03		

filopodium membrane	4.34E-03	intramolecular oxidoreductase activity	1.34 E-02	multicellular organism development	2.75E -04
filopodium	2.67E-02	isomerase activity	4.23 E-02	multicellular organismal process	4.13E -03
platelet alpha granule	4.80E-03	low-density lipoprotein particle binding	4.12 E-03	blood vessel development	1.88E -04
cytoplasmic vesicle	8.86E-03	lipoprotein particle binding	6.85 E-03	vasculature development	3.17E -06
intracellular vesicle	8.95E-03	protein-lipid complex binding	6.85 E-03	system development	3.73E -03
vesicle	1.06E-02	C-C chemokine receptor activity	5.48 E-03	circulatory system development	2.41E -05
lamellipodium membrane	5.26E-03	chemokine receptor activity	5.71 E-03	negative regulation of nitric oxide mediated signal transduction	4.58E -04
leading edge membrane	4.61E-02	cytokine receptor activity	2.20 E-02	regulation of nitric oxide mediated signal transduction	2.52E -03
lamellipodium	4.89E-02	immune receptor activity	3.12 E-02	negative regulation of dendritic cell antigen processing and presentation	6.87E -04
microvillus membrane	7.76E-03	G protein-coupled chemoattractant receptor activity	5.71 E-03	negative regulation of antigen processing and presentation	1.60E -03
microvillus	2.74E-02	heparan sulfate proteoglycan binding	5.71 E-03	regulation of antigen processing and presentation	4.80E -03
sarcoplasmic reticulum	1.84E-02	proteoglycan binding	1.00 E-02	regulation of dendritic cell antigen processing and presentation	2.29E -03
sarcoplasm	2.47E-02	sulfur compound binding	1.84 E-03	negative regulation of antigen processing and presentation of peptide or polysaccharide antigen via MHC class II	6.87E -04
ruffle membrane	2.31E-02	transforming growth factor beta binding	5.71 E-03	regulation of antigen processing and presentation of peptide or polysaccharide antigen via MHC class II	1.14E -03
ruffle	4.39E-02	C-C chemokine binding	5.71 E-03	immune complex clearance	6.87E -04
melanosome	2.45E-02	laminin binding	7.31 E-03	monocyte aggregation	6.29E -07
pigment granule	2.45E-02	extracellular matrix binding	1.01 E-04	leukocyte aggregation	3.81E -06
external side of plasma membrane	1.87E-06	protein kinase C binding	1.39 E-02	leukocyte cell-cell adhesion	8.15E -05
cell surface	3.48E-05	enzyme binding	1.07 E-02	cell-cell adhesion	1.07E -04
side of membrane	1.16E-05	RNA polymerase binding	1.46 E-02	cell adhesion	6.84E -04
focal adhesion	4.47E-02	phosphatidylserine binding	1.62 E-02	negative regulation of endothelial cell chemotaxis	9.16E -04
cell-substrate junction	4.78E-02	modified amino acid binding	2.61 E-02	regulation of endothelial cell chemotaxis	5.26E -03
		integrin binding	4.94 E-04	regulation of locomotion	2.49E -02
		cell adhesion molecule binding	1.99 E-03	regulation of response to external stimulus	2.52E -02

heparin binding	6.21E-04	regulation of endothelial cell migration	6.65E-04
glycosaminoglycan binding	1.23E-03	regulation of cell migration	2.05E-02
protease binding	7.33E-04	regulation of cell motility	2.29E-02
extracellular matrix structural constituent	3.21E-02	negative regulation of chemotaxis	1.03E-02
endopeptidase inhibitor activity	4.39E-02	negative regulation of endothelial cell migration	1.30E-02
endopeptidase regulator activity	4.94E-02	regulation of complement activation, alternative pathway	9.16E-04
peptidase inhibitor activity	4.56E-02	regulation of response to stress	3.94E-02
identical protein binding	1.27E-02	regulation of complement activation	5.26E-03
		regulation of humoral immune response	1.14E-02
		regulation of postsynaptic neurotransmitter receptor diffusion trapping	9.16E-04
		regulation of receptor localization to synapse	8.90E-03
		regulation of localization	9.93E-03
		regulation of biological quality	2.07E-03
		regulation of cellular localization	2.44E-02
		negative regulation of long-chain fatty acid import across plasma membrane	9.16E-04
		negative regulation of transmembrane transport	2.96E-02
		negative regulation of transport	5.00E-03
		regulation of transport	5.58E-03
		regulation of transmembrane transport	5.22E-03
		regulation of long-chain fatty acid import across plasma membrane	1.60E-03
		regulation of long-chain fatty acid import into cell	2.06E-03
		regulation of fatty acid transport	9.35E-03
		regulation of lipid transport	5.06E-04
		regulation of lipid localization	7.87E-04
		regulation of organic acid transport	2.40E-02

negative regulation of long-chain fatty acid import into cell	9.16E-04
negative regulation of fatty acid transport	2.29E-03
negative regulation of organic acid transport	6.85E-03
negative regulation of lipid transport	2.35E-05
negative regulation of lipid localization	8.67E-05
mast cell chemotaxis	1.14E-03
leukocyte chemotaxis	3.14E-02
cell chemotaxis	4.85E-02
chemotaxis	2.22E-03
taxis	2.25E-03
response to external stimulus	3.75E-04
locomotion	2.71E-03
response to chemical	5.01E-03
cellular response to chemical stimulus	3.92E-04
cellular response to stimulus	3.87E-02
mast cell migration	1.83E-03
myeloid leukocyte migration	3.19E-02
apolipoprotein A-I-mediated signaling pathway	1.14E-03
negative regulation of low-density lipoprotein receptor activity	1.14E-03
negative regulation of receptor-mediated endocytosis	6.85E-03
negative regulation of endocytosis	1.64E-02
regulation of receptor-mediated endocytosis	3.23E-02
negative regulation of molecular function	1.14E-02
regulation of molecular function	5.79E-03
negative regulation of lipoprotein metabolic process	1.14E-03

	negative regulation of protein metabolic process	1.41E-02
	regulation of protein metabolic process	7.76E-03
	regulation of lipoprotein metabolic process	4.57E-03
	regulation of serotonin uptake	1.37E-03
	regulation of neurotransmitter uptake	4.80E-03
	regulation of neurotransmitter transport	3.01E-02
	negative regulation of cGMP-mediated signaling	1.37E-03
	regulation of cGMP-mediated signaling	3.43E-03
	negative regulation of blood vessel endothelial cell proliferation involved in sprouting angiogenesis	1.60E-03
	negative regulation of endothelial cell proliferation	1.07E-02
	negative regulation of epithelial cell proliferation	3.79E-02
	regulation of epithelial cell proliferation	7.31E-05
	regulation of cell population proliferation	1.91E-04
	regulation of endothelial cell proliferation	3.34E-06
	regulation of blood vessel endothelial cell proliferation involved in sprouting angiogenesis	3.89E-03
	positive regulation of glomerular mesangial cell proliferation	1.83E-03
	positive regulation of cell proliferation involved in kidney development	2.29E-03
	positive regulation of cell population proliferation	2.24E-05
	regulation of cell proliferation involved in kidney development	3.66E-03
	regulation of glomerular mesangial cell proliferation	2.74E-03
	negative regulation of plasminogen activation	1.83E-03
	negative regulation of protein processing	5.94E-03
	regulation of protein processing	1.55E-02
	regulation of protein maturation	1.64E-02
	regulation of proteolysis	8.14E-03

	negative regulation of protein maturation	5.94E-03
	regulation of plasminogen activation	3.89E-03
	engulfment of apoptotic cell	2.06E-03
	apoptotic cell clearance	2.49E-05
	phagocytosis	4.81E-04
	endocytosis	4.98E-03
	vesicle-mediated transport	3.12E-02
	import into cell	9.15E-03
	phagocytosis, engulfment	1.03E-02
	plasma membrane invagination	1.23E-02
	membrane invagination	1.39E-02
	cellular component organization	2.33E-02
	cellular component organization or biogenesis	2.62E-02
	regulation of complement-dependent cytotoxicity	2.06E-03
	regulation of cell killing	3.45E-02
	negative regulation of fibrinolysis	2.29E-03
	positive regulation of blood coagulation	5.94E-03
	positive regulation of coagulation	7.08E-03
	regulation of coagulation	1.71E-02
	regulation of multicellular organismal process	2.62E-02
	positive regulation of multicellular organismal process	5.67E-03
	positive regulation of hemostasis	5.94E-03
	regulation of hemostasis	1.62E-02
	regulation of body fluid levels	4.34E-05
	positive regulation of wound healing	1.46E-02

	positive regulation of response to wounding	1.84E-02	
	regulation of response to wounding	3.99E-02	
	regulation of wound healing	2.99E-02	
	regulation of blood coagulation	1.57E-02	
	regulation of fibrinolysis	3.20E-03	
	negative regulation of macrophage derived foam cell differentiation	2.29E-03	
	negative regulation of cell differentiation	1.02E-02	
	regulation of developmental process	1.01E-03	
	negative regulation of developmental process	9.52E-04	
	regulation of macrophage derived foam cell differentiation	5.71E-03	
	retinal pigment epithelium development	2.29E-03	
	tissue development	6.32E-03	
	retina development in camera-type eye	4.45E-02	
	sensory organ development	9.44E-03	
	animal organ development	2.41E-02	
	complement activation, alternative pathway	2.52E-03	
	defense response	3.12E-03	
	response to stress	3.08E-03	
	nitric oxide-cGMP-mediated signaling	2.52E-03	
	nitric oxide mediated signal transduction	5.48E-03	
	intracellular signaling cassette	1.32E-02	
	cGMP-mediated signaling	7.53E-03	
	positive regulation of fibroblast migration	1.26E-05	
	positive regulation of cell migration	7.39E-03	
	positive regulation of cell motility	8.11E-03	

	positive regulation of locomotion	8.53E-03
	regulation of fibroblast migration	5.32E-05
	cellular response to insulin-like growth factor stimulus	2.97E-03
	response to nitrogen compound	2.56E-02
	response to oxygen-containing compound	4.73E-03
	cellular response to oxygen-containing compound	1.51E-03
	cellular response to nitrogen compound	8.79E-03
	positive regulation of extrinsic apoptotic signaling pathway via death domain receptors	3.20E-03
	positive regulation of extrinsic apoptotic signaling pathway	1.55E-02
	regulation of extrinsic apoptotic signaling pathway	3.97E-02
	regulation of apoptotic process	3.54E-03
	regulation of programmed cell death	3.86E-03
	positive regulation of apoptotic signaling pathway	3.81E-02
	regulation of extrinsic apoptotic signaling pathway via death domain receptors	1.14E-02
	positive regulation of osteoblast proliferation	3.43E-03
	regulation of osteoblast proliferation	7.08E-03
	blood coagulation, fibrin clot formation	3.43E-03
	protein activation cascade	4.34E-03
	protein metabolic process	3.19E-02
	blood coagulation	3.86E-04
	coagulation	3.86E-04
	hemostasis	4.03E-04
	wound healing	1.71E-03
	response to wounding	3.57E-03
	negative regulation of sprouting angiogenesis	3.43E-03

	negative regulation of angiogenesis	2.65E-02	
	negative regulation of blood vessel morphogenesis	2.70E-02	
	regulation of anatomical structure morphogenesis	7.45E-04	
	negative regulation of vasculature development	2.72E-02	
	regulation of vasculature development	3.05E-05	
	regulation of multicellular organismal development	3.50E-03	
	regulation of angiogenesis	2.86E-05	
	regulation of sprouting angiogenesis	1.09E-02	
	retinal rod cell development	3.43E-03	
	retinal rod cell differentiation	5.71E-03	
	camera-type eye photoreceptor cell differentiation	9.58E-03	
	neural retina development	2.11E-02	
	retina morphogenesis in camera-type eye	1.91E-02	
	camera-type eye morphogenesis	3.59E-02	
	eye morphogenesis	4.25E-02	
	animal organ morphogenesis	2.31E-02	
	eye photoreceptor cell differentiation	1.43E-02	
	photoreceptor cell differentiation	1.91E-02	
	eye photoreceptor cell development	1.00E-02	
	photoreceptor cell development	1.46E-02	
	negative regulation of cell migration involved in sprouting angiogenesis	3.89E-03	
	regulation of cell migration involved in sprouting angiogenesis	9.35E-03	
	regulation of blood vessel endothelial cell migration	2.29E-02	
	negative regulation of blood vessel endothelial cell migration	8.44E-03	
	regulation of trophoblast cell migration	3.89E-03	

	cellular response to nitric oxide	4.12E-03	
	response to nitric oxide	5.48E-03	
	cellular response to reactive nitrogen species	4.57E-03	
	inflammatory response to wounding	4.12E-03	
	inflammatory response	1.87E-04	
	platelet aggregation	2.63E-05	
	platelet activation	8.15E-05	
	cell activation	1.26E-02	
	homotypic cell-cell adhesion	6.66E-05	
	maintenance of postsynaptic specialization structure	4.12E-03	
	postsynaptic density organization	8.22E-03	
	postsynaptic specialization organization	1.00E-02	
	postsynapse organization	3.12E-02	
	maintenance of synapse structure	9.58E-03	
	cell junction maintenance	1.57E-02	
	cellular component maintenance	1.95E-02	
	negative regulation of interleukin-12 production	4.34E-03	
	regulation of interleukin-12 production	1.46E-02	
	positive regulation of transforming growth factor beta production	4.57E-03	
	regulation of transforming growth factor beta production	7.76E-03	
	positive regulation of gene expression	3.15E-02	
	positive regulation of metabolic process	3.86E-02	
	positive regulation of macromolecule metabolic process	3.01E-02	
	negative regulation of fibroblast growth factor receptor signaling pathway	4.57E-03	
	negative regulation of cellular response to growth factor stimulus	2.47E-02	

	regulation of fibroblast growth factor receptor signaling pathway	7.99E-03
	smooth muscle cell migration	4.57E-03
	muscle cell migration	8.44E-03
	mitochondrial DNA metabolic process	4.80E-03
	mitochondrial genome maintenance	7.76E-03
	eosinophil chemotaxis	4.80E-03
	eosinophil migration	5.03E-03
	granulocyte migration	2.29E-02
	granulocyte chemotaxis	1.84E-02
	positive regulation of protein export from nucleus	4.80E-03
	regulation of protein export from nucleus	9.13E-03
	regulation of intracellular protein transport	3.97E-02
	regulation of protein localization	2.00E-02
	regulation of nucleocytoplasmic transport	3.12E-02
	positive regulation of intracellular protein transport	2.52E-02
	positive regulation of intracellular transport	3.48E-02
	positive regulation of nucleocytoplasmic transport	1.75E-02
	growth plate cartilage development	5.03E-03
	connective tissue development	1.48E-03
	endochondral bone growth	6.85E-03
	bone growth	7.99E-03
	organ growth	3.12E-02
	bone development	1.07E-03
	skeletal system development	5.79E-03
	cartilage development involved in endochondral bone morphogenesis	8.67E-03

	cartilage development	8.12E-04
	endochondral bone morphogenesis	1.62E-02
	bone morphogenesis	2.58E-02
	positive regulation of cell adhesion mediated by integrin	5.26E-03
	positive regulation of cell adhesion	5.42E-03
	regulation of cell adhesion	1.33E-02
	regulation of cell adhesion mediated by integrin	1.12E-02
	response to magnesium ion	5.26E-03
	positive regulation of endothelial cell apoptotic process	5.48E-03
	regulation of endothelial cell apoptotic process	7.14E-05
	neutrophil homeostasis	5.71E-03
	myeloid cell homeostasis	4.03E-02
	leukocyte homeostasis	2.85E-02
	positive regulation of macrophage chemotaxis	5.71E-03
	positive regulation of leukocyte chemotaxis	2.40E-02
	positive regulation of chemotaxis	3.63E-02
	positive regulation of leukocyte migration	6.00E-04
	regulation of leukocyte migration	1.26E-03
	regulation of leukocyte chemotaxis	3.03E-02
	positive regulation of macrophage migration	7.08E-03
	positive regulation of mononuclear cell migration	2.18E-04
	regulation of mononuclear cell migration	4.69E-04
	regulation of macrophage migration	1.12E-02
	regulation of granulocyte chemotaxis	1.39E-02
	regulation of macrophage chemotaxis	7.76E-03

	mesodermal cell differentiation	5.94E-03
	mesoderm formation	1.68E-02
	mesoderm morphogenesis	1.77E-02
	mesoderm development	2.79E-02
	formation of primary germ layer	2.76E-02
	anatomical structure formation involved in morphogenesis	1.06E-03
	gastrulation	4.05E-02
	positive regulation of bone resorption	6.17E-03
	regulation of bone resorption	1.21E-02
	regulation of bone remodeling	1.43E-02
	regulation of tissue remodeling	1.95E-02
	peptide cross-linking	6.17E-03
	cellular response to platelet-derived growth factor stimulus	6.40E-03
	response to platelet-derived growth factor	7.08E-03
	positive regulation of macrophage activation	7.08E-03
	regulation of macrophage activation	1.43E-02
	behavioral response to pain	7.31E-03
	response to pain	1.16E-02
	multicellular organismal response to stress	2.56E-02
	positive regulation of transforming growth factor beta receptor signaling pathway	7.31E-03
	positive regulation of cellular response to transforming growth factor beta stimulus	7.31E-03
	regulation of cellular response to transforming growth factor beta stimulus	3.41E-02
	regulation of transforming growth factor beta receptor signaling pathway	3.34E-02
	positive regulation of transmembrane receptor protein serine/threonine kinase signaling pathway	2.45E-02
	negative regulation of endothelial cell apoptotic process	7.76E-03

	negative regulation of apoptotic process	8.52E-04	
	negative regulation of programmed cell death	9.44E-04	
	vascular associated smooth muscle cell differentiation	7.76E-03	
	smooth muscle cell differentiation	1.23E-02	
	negative regulation of lipid storage	7.99E-03	
	regulation of lipid storage	1.48E-02	
	heterotypic cell-cell adhesion	8.44E-03	
	ventricular system development	8.44E-03	
	negative regulation of cell-matrix adhesion	8.44E-03	
	negative regulation of cell-substrate adhesion	1.43E-02	
	regulation of cell-substrate adhesion	1.10E-03	
	regulation of cell-matrix adhesion	3.42E-04	
	positive regulation of T cell migration	8.44E-03	
	positive regulation of lymphocyte migration	1.03E-02	
	regulation of lymphocyte migration	1.62E-02	
	regulation of T cell migration	1.19E-02	
	regulation of postsynaptic neurotransmitter receptor internalization	8.90E-03	
	regulation of receptor internalization	2.16E-02	
	positive regulation of osteoclast differentiation	9.13E-03	
	regulation of osteoclast differentiation	2.02E-02	
	regulation of myeloid leukocyte differentiation	3.37E-02	
	positive regulation of myeloid leukocyte differentiation	1.75E-02	
	positive regulation of leukocyte differentiation	4.89E-02	
	positive regulation of hemopoiesis	4.89E-02	
	positive regulation of developmental process	3.06E-03	

positive regulation of myeloid cell differentiation	2.67E-02
negative chemotaxis	9.35E-03
positive regulation of substrate adhesion-dependent cell spreading	9.35E-03
positive regulation of cell-substrate adhesion	4.09E-04
regulation of substrate adhesion-dependent cell spreading	1.34E-02
cell-substrate junction assembly	9.58E-03
cell-substrate junction organization	1.00E-02
cell adhesion mediated by integrin	1.00E-02
response to dietary excess	1.05E-02
energy homeostasis	2.27E-02
substrate adhesion-dependent cell spreading	1.12E-02
cell-substrate adhesion	4.19E-02
positive regulation of G1/S transition of mitotic cell cycle	1.14E-02
regulation of G1/S transition of mitotic cell cycle	3.77E-02
regulation of cell cycle G1/S phase transition	4.39E-02
positive regulation of cell cycle G1/S phase transition	1.48E-02
positive regulation of cell cycle phase transition	2.70E-02
positive regulation of mitotic cell cycle phase transition	2.22E-02
positive regulation of mitotic cell cycle	3.03E-02
determination of adult lifespan	1.14E-02
endocardial cushion development	1.16E-02
positive regulation of endothelial cell proliferation	2.23E-04
positive regulation of epithelial cell proliferation	1.18E-03
regulation of protein tyrosine kinase activity	1.21E-02
regulation of protein kinase activity	4.45E-03

	regulation of kinase activity	5.06E-03
	regulation of phosphorylation	1.88E-02
	regulation of phosphate metabolic process	2.40E-02
	regulation of phosphorus metabolic process	2.41E-02
	regulation of transferase activity	6.61E-03
	regulation of catalytic activity	1.64E-03
	regulation of protein phosphorylation	1.68E-02
	regulation of protein modification process	2.70E-02
	embryo implantation	1.28E-02
	female pregnancy	4.63E-02
	positive regulation of angiogenesis	5.35E-06
	positive regulation of vasculature development	5.63E-06
	ERK1 and ERK2 cascade	1.37E-02
	MAPK cascade	4.76E-02
	positive regulation of smooth muscle cell proliferation	3.06E-04
	regulation of smooth muscle cell proliferation	8.12E-04
	nucleosome assembly	1.41E-02
	protein-DNA complex assembly	3.52E-02
	protein-DNA complex organization	3.97E-02
	nucleosome organization	1.82E-02
	positive regulation of blood vessel endothelial cell migration	1.41E-02
	positive regulation of endothelial cell migration	2.72E-04
	sprouting angiogenesis	1.43E-02
	angiogenesis	2.58E-03
	glomerulus development	1.48E-02

	nephron development	3.50E-02
	symbiont entry into host cell	1.48E-02
	symbiont entry into host	1.48E-02
	biological process involved in interaction with host	2.47E-02
	biological process involved in symbiotic interaction	4.81E-02
	viral life cycle	2.70E-02
	viral process	3.39E-02
	positive regulation of cell-matrix adhesion	1.50E-02
	regulation of extracellular matrix organization	1.52E-02
	chemokine-mediated signaling pathway	1.57E-02
	cellular response to chemokine	1.82E-02
	response to chemokine	1.82E-02
	response to cytokine	1.82E-02
	response to peptide	1.88E-02
	activation of protein kinase activity	1.64E-02
	positive regulation of protein kinase activity	1.63E-03
	positive regulation of protein phosphorylation	7.25E-03
	positive regulation of phosphorylation	7.84E-03
	positive regulation of phosphate metabolic process	9.41E-03
	positive regulation of phosphorus metabolic process	9.41E-03
	positive regulation of protein modification process	1.15E-02
	positive regulation of protein metabolic process	1.61E-03
	positive regulation of kinase activity	1.77E-03
	positive regulation of transferase activity	2.38E-03
	positive regulation of catalytic activity	3.41E-04

	positive regulation of molecular function	1.09E-03	
	positive regulation of smooth muscle cell migration	1.64E-02	
	regulation of smooth muscle cell migration	2.47E-02	
	cellular response to mechanical stimulus	1.71E-02	
	response to mechanical stimulus	1.19E-03	
	response to abiotic stimulus	2.79E-02	
	cellular response to external stimulus	1.75E-02	
	G1/S transition of mitotic cell cycle	1.73E-02	
	mitotic cell cycle phase transition	3.19E-02	
	cell cycle phase transition	3.50E-02	
	cell cycle G1/S phase transition	1.77E-02	
	positive regulation of fibroblast proliferation	1.77E-02	
	regulation of fibroblast proliferation	2.85E-02	
	positive regulation of reactive oxygen species metabolic process	1.86E-02	
	regulation of reactive oxygen species metabolic process	3.66E-02	
	negative regulation of cysteine-type endopeptidase activity	1.91E-02	
	negative regulation of endopeptidase activity	3.19E-02	
	regulation of endopeptidase activity	1.65E-03	
	regulation of peptidase activity	1.99E-03	
	regulation of hydrolase activity	7.46E-03	
	negative regulation of peptidase activity	3.54E-02	
	negative regulation of hydrolase activity	4.81E-02	
	regulation of cysteine-type endopeptidase activity	8.86E-04	
	regulation of release of sequestered calcium ion into cytosol	1.91E-02	
	regulation of sequestering of calcium ion	3.05E-02	

	regulation of calcium ion transmembrane transport	4.23E-02	
	organ or tissue specific immune response	1.95E-02	
	integrin-mediated signaling pathway	1.98E-02	
	response to activity	1.98E-02	
	outflow tract morphogenesis	2.00E-02	
	platelet-derived growth factor receptor signaling pathway	2.02E-02	
	positive regulation of MAP kinase activity	2.29E-02	
	positive regulation of protein serine/threonine kinase activity	3.48E-02	
	regulation of MAP kinase activity	3.57E-02	
	positive regulation of MAPK cascade	5.60E-03	
	positive regulation of intracellular signal transduction	2.63E-02	
	regulation of MAPK cascade	1.07E-02	
	response to unfolded protein	2.34E-02	
	response to topologically incorrect protein	2.74E-02	
	roof of mouth development	2.36E-02	
	positive regulation of tumor necrosis factor production	2.74E-02	
	regulation of tumor necrosis factor production	4.45E-02	
	regulation of tumor necrosis factor superfamily cytokine production	4.52E-02	
	positive regulation of tumor necrosis factor superfamily cytokine production	2.79E-02	
	positive regulation of cysteine-type endopeptidase activity	2.94E-02	
	positive regulation of endopeptidase activity	3.28E-02	
	positive regulation of peptidase activity	3.50E-02	
	cell-matrix adhesion	2.99E-02	
	positive regulation of translation	3.19E-02	
	mitochondrial gene expression	3.32E-02	

	response to calcium ion	3.37E-02
	response to glucose	3.54E-02
	response to hexose	3.68E-02
	response to monosaccharide	3.86E-02
	response to carbohydrate	4.41E-02
	visual perception	3.66E-02
	sensory perception of light stimulus	3.74E-02
	ATP metabolic process	3.77E-02
	purine ribonucleoside triphosphate metabolic process	4.30E-02
	purine nucleoside triphosphate metabolic process	4.47E-02
	nucleoside triphosphate metabolic process	4.81E-02
	ribonucleoside triphosphate metabolic process	4.43E-02
	positive regulation of peptidyl-tyrosine phosphorylation	3.79E-02
	calcium-mediated signaling	3.83E-02
	positive regulation of cytosolic calcium ion concentration	4.43E-02
	positive regulation of phosphatidylinositol 3-kinase/protein kinase B signal transduction	4.47E-02
	neuromuscular process	4.59E-02
	cellular response to xenobiotic stimulus	4.81E-02
	positive regulation of ERK1 and ERK2 cascade	4.92E-02

Supplemental Table 4. Complete Annotations Lists with Accompanying P-values from Gene Ontology Analysis of Genes Differentially Expressed in Post-AZA treated, Responsive Patient PBMC Compared to Pre-treated matched, PBMC.

Gene Ontology Cellular Compartment	p-value	Gene Ontology Molecular Function	p-value	Gene Ontology Biological Process	p-value	PANTHER Pathway	p-value
stereocilium coat	8.41E-03	cyanamide hydratase activity	7.03E-05	positive regulation of glutathione peroxidase activity	8.41E-03	Blood coagulation (P00011)	2.01E-06
cellular anatomical structure	1.00E-03	NADP phosphatase activity	8.41E-03	regulation of glutathione peroxidase activity	8.41E-03	T cell activation (P00053)	8.17E-05
glycocalyx	8.41E-03	methanethiol oxidase activity	8.41E-03	regulation of peroxidase activity	1.67E-02	GABA-B receptor II signaling (P05731)	3.86E-02
external encapsulating structure	8.74E-03	oxidoreductase activity	2.24E-02	regulation of catalytic activity	4.45E-02	Dopamine receptor mediated signaling pathway (P05912)	1.28E-02
cell periphery	2.93E-11	high-affinity IgM receptor activity	8.41E-03	biological regulation	4.93E-05	Integrin signalling pathway (P00034)	2.26E-04
secretory dimeric IgA immunoglobulin complex	5.84E-07	IgM receptor activity	8.41E-03	positive regulation of peroxidase activity	1.67E-02	PDGF signaling pathway (P00047)	3.35E-02
secretory IgA immunoglobulin complex	5.77E-06	immune receptor activity	2.01E-03	positive regulation of cellular process	2.78E-05	Inflammation mediated by chemokine and cytokine signaling pathway (P00031)	6.82E-03
polymeric IgA immunoglobulin complex	5.77E-06	signaling receptor activity	3.96E-04	positive regulation of biological process	5.80E-08		
IgA immunoglobulin complex, circulating	5.77E-06	molecular transducer activity	3.96E-04	regulation of biological process	2.68E-05		
immunoglobulin complex, circulating	2.99E-08	transmembrane signaling receptor activity	2.75E-02	regulation of cellular process	4.30E-05		
extracellular space	2.72E-06	chemokine receptor antagonist activity	8.41E-03	positive regulation of catalytic activity	3.17E-02		

extracellular region	4.75E-05	signaling receptor binding	4.77E-09	apoptotic process in bone marrow cell	8.41E-03
immunoglobulin complex	1.46E-03	protein binding	2.57E-06	programmed cell death	2.66E-02
protein-containing complex	2.82E-02	binding	1.94E-05	cell death	3.86E-02
IgA immunoglobulin complex	9.74E-07	thiomorpholine-carboxylate dehydrogenase activity	8.41E-03	cellular process	7.02E-04
dimeric IgA immunoglobulin complex	5.84E-07	CCR4 chemokine receptor binding	8.41E-03	natural killer cell mediated cytotoxicity directed against tumor cell target	8.41E-03
cytolytic granule lumen	7.03E-05	chemokine receptor binding	2.88E-04	natural killer cell mediated immune response to tumor cell	8.41E-03
cytoplasm	6.31E-03	cytokine receptor binding	2.73E-02	immune response to tumor cell	1.44E-03
cytoplasmic vesicle lumen	6.81E-03	G protein-coupled receptor binding	4.20E-05	response to tumor cell	1.72E-02
cytoplasmic vesicle	7.18E-04	NADPH phosphatase activity	8.41E-03	response to biotic stimulus	8.63E-11
intracellular vesicle	1.03E-03	thromboxane A2 receptor activity	8.41E-03	response to stimulus	1.36E-14
vesicle	6.93E-06	thromboxane receptor activity	8.41E-03	immune response	1.93E-13
vesicle lumen	6.93E-03	hemoglobin alpha binding	2.40E-08	immune system process	4.52E-17
cytolytic granule	7.46E-08	hemoglobin binding	2.66E-11	natural killer cell mediated immunity	6.56E-08
cyclin D2-CDK6 complex	8.41E-03	haptoglobin binding	2.66E-11	innate immune response	3.63E-03
monomeric IgA immunoglobulin complex	2.32E-06	pore-forming activity	2.10E-04	defense response to symbiont	2.52E-08
laminin-11 complex	2.10E-04	polymeric immunoglobulin binding	1.67E-02	defense response to other organism	1.56E-08
laminin complex	2.43E-03	immunoglobulin binding	2.00E-02	response to other organism	3.28E-11

basement membrane	9.12E-03	protein-containing complex binding	1.11E-05	biological process involved in interspecies interaction between organisms	4.00E-12
collagen-containing extracellular matrix	2.96E-02	iron ion sequestering activity	1.67E-02	response to external biotic stimulus	3.49E-11
extracellular matrix	2.97E-02	metal ion sequestering activity	4.13E-02	response to external stimulus	1.77E-10
integrin alphaIIb-beta3 complex	2.10E-04	5-aminolevulinate synthase activity	1.67E-02	defense response	1.93E-08
integrin complex	1.27E-04	N-succinyltransferase activity	1.67E-02	response to stress	5.78E-09
plasma membrane signaling receptor complex	2.06E-08	succinyltransferase activity	2.50E-02	lymphocyte mediated immunity	8.03E-11
plasma membrane protein complex	4.37E-06	structural constituent of synapse-associated extracellular matrix	1.67E-02	leukocyte mediated immunity	1.61E-09
membrane protein complex	1.16E-03	structural molecule activity	4.46E-02	immune effector process	1.08E-09
membrane	4.54E-05	HLA-E specific inhibitory MHC class Ib receptor activity	1.67E-02	natural killer cell mediated cytotoxicity	1.14E-06
plasma membrane	7.12E-10	inhibitory MHC class Ib receptor activity	1.67E-02	leukocyte mediated cytotoxicity	1.33E-08
receptor complex	3.80E-06	MHC class Ib receptor activity	1.44E-03	cell killing	2.72E-10
protein complex involved in cell adhesion	5.50E-07	MHC class Ib protein binding, via antigen binding groove	1.67E-02	negative regulation of epithelial cell proliferation involved in lung morphogenesis	8.41E-03
hemoglobin complex	8.42E-13	MHC class Ib protein complex binding	3.32E-02	regulation of epithelial cell proliferation	1.10E-02
haptoglobin-hemoglobin complex	6.61E-11	MHC protein complex binding	3.86E-02	regulation of cell population proliferation	1.85E-03

glycoprotein Ib-IX-V complex	5.77E-06	MHC protein binding	3.48E-04	negative regulation of epithelial cell proliferation	2.86E-02
glycoprotein complex	9.14E-04	enterobactin binding	1.67E-02	negative regulation of cellular process	3.52E-04
platelet-derived growth factor complex	1.67E-02	FBXO family protein binding	1.67E-02	negative regulation of biological process	2.13E-04
gamma-delta T cell receptor complex	1.15E-05	interleukin-7 receptor activity	1.67E-02	metanephric glomerular basement membrane development	8.41E-03
T cell receptor complex	3.31E-04	cytokine receptor activity	4.73E-02	extracellular matrix organization	9.64E-03
caveolar macromolecular signaling complex	1.67E-02	asparagine-tRNA ligase activity	1.67E-02	external encapsulating structure organization	9.99E-03
membrane raft	3.72E-02	oxygen carrier activity	5.29E-10	extracellular structure organization	9.81E-03
membrane microdomain	3.83E-02	molecular carrier activity	2.00E-02	anatomical structure development	2.96E-04
neurofilament cytoskeleton	1.67E-02	C-4 methylsterol oxidase activity	2.50E-02	developmental process	4.50E-04
alpha9-beta1 integrin-ADAM8 complex	1.67E-02	IgM binding	2.50E-02	multicellular organismal process	7.79E-08
interleukin-2 receptor complex	1.67E-02	CD8 receptor binding	2.50E-02	CD8-positive, alpha-beta T cell differentiation involved in immune response	8.41E-03
integrin alphav-beta5 complex	1.67E-02	Fc-gamma receptor I complex binding	2.50E-02	alpha-beta T cell activation	4.32E-09
integrin alpha9-beta1 complex	1.67E-02	immunoglobulin receptor binding	4.19E-07	T cell activation	7.46E-11
cyclin D3-CDK6 complex	1.67E-02	procollagen-lysine 5-dioxygenase activity	2.50E-02	lymphocyte activation	5.91E-12
cyclin D1-CDK6 complex	1.67E-02	peptidyl-lysine 5-dioxygenase activity	3.32E-02	leukocyte activation	2.47E-12
ankyrin-1 complex	3.17E-05	arylesterase activity	1.03E-03	cell activation	1.28E-17
platelet alpha granule membrane	3.71E-09	interleukin-2 binding	2.50E-02	alpha-beta T cell differentiation	6.41E-04

secretory granule membrane	1.94E-02	growth factor binding	1.03E-03	T cell differentiation	6.71E-09
secretory granule	2.38E-04	IgA binding	2.50E-02	lymphocyte differentiation	1.12E-07
secretory vesicle	9.70E-04	V1A vasopressin receptor binding	2.50E-02	mononuclear cell differentiation	4.11E-07
platelet alpha granule	2.85E-10	vasopressin receptor binding	2.50E-02	leukocyte differentiation	3.33E-06
laminin-10 complex	2.50E-02	G protein-coupled serotonin receptor binding	2.50E-02	hemopoiesis	7.98E-10
alpha-beta T cell receptor complex	2.27E-06	interleukin-15 receptor activity	2.50E-02	cell development	4.20E-06
laminin-3 complex	2.50E-02	interleukin-2 receptor activity	2.50E-02	cell differentiation	3.81E-05
laminin-1 complex	2.50E-02	fibrinogen binding	1.44E-03	cellular developmental process	3.86E-05
Fc-gamma receptor III complex	2.50E-02	MHC class I protein complex binding	1.44E-03	alpha-beta T cell differentiation involved in immune response	8.57E-03
Fc receptor complex	4.13E-02	myosin light chain kinase activity	3.32E-02	T cell differentiation involved in immune response	1.06E-02
alphav-beta3 integrin-HMGB1 complex	2.50E-02	acetylcholine receptor activator activity	3.32E-02	T cell activation involved in immune response	4.14E-02
alphav-beta3 integrin-PKCalpha complex	2.50E-02	adenylate cyclase inhibitor activity	3.32E-02	lymphocyte activation involved in immune response	3.49E-02
collagen type V trimer	2.50E-02	cyclase inhibitor activity	4.13E-02	leukocyte activation involved in immune response	3.19E-02
supramolecular fiber	2.31E-02	sphingosine-1-phosphate receptor activity	2.43E-03	cell activation involved in immune response	3.42E-02
supramolecular polymer	2.40E-02	bioactive lipid receptor activity	8.79E-03	alpha-beta T cell activation involved in immune response	8.57E-03
pentameric IgM immunoglobulin complex	2.50E-02	neuregulin binding	4.13E-02	olfactory bulb axon guidance	8.41E-03

IgM immunoglobulin complex, circulating	2.50E-02	C-X3-C chemokine binding	4.13E-02	anatomical structure morphogenesis	3.62E-02
IgM immunoglobulin complex	3.02E-03	chemokine binding	3.13E-02	cell morphogenesis	1.86E-02
integrin alphav-beta3 complex	2.50E-02	receptor signaling protein tyrosine kinase activator activity	4.13E-02	positive regulation of piRNA transcription	8.41E-03
alphav-beta3 integrin-vitronectin complex	2.50E-02	kainate selective glutamate receptor activity	4.13E-02	regulation of piRNA transcription	8.41E-03
spectrin-associated cytoskeleton	4.72E-05	thyroid hormone binding	4.13E-02	positive regulation of metabolic process	1.18E-02
postsynaptic specialization of symmetric synapse	2.50E-02	integrin binding involved in cell-matrix adhesion	4.13E-02	positive regulation of biosynthetic process	2.30E-02
cell junction	8.32E-03	integrin binding	6.68E-05	thromboxane A2 signaling pathway	8.41E-03
IgG immunoglobulin complex	3.25E-06	cell adhesion molecule binding	2.75E-03	signal transduction	4.06E-09
glycinergic synapse	1.44E-03	cell adhesion mediator activity	2.62E-02	cellular response to stimulus	5.53E-08
actin cap	3.32E-02	thrombin-activated receptor activity	4.13E-02	signaling	1.61E-08
cortical actin cytoskeleton	4.64E-03	proteinase-activated receptor activity	4.13E-02	cell communication	1.91E-08
actin cytoskeleton	5.62E-04	pyruvate dehydrogenase (acetyl-transferring) kinase activity	4.13E-02	integrin biosynthetic process	8.41E-03
cortical cytoskeleton	3.91E-06	CXCR3 chemokine receptor binding	4.13E-02	plasma membrane organization	3.96E-02

cell cortex	1.00E-04	CXCR chemokine receptor binding	1.05E-05	cytotoxic T cell differentiation	8.41E-03
alphav-beta3 integrin-IGF-1-IGF1R complex	3.32E-02	oxygen binding	2.45E-07	negative regulation of norepinephrine uptake	8.41E-03
cytolytic granule membrane	3.32E-02	T cell receptor binding	3.68E-03	regulation of norepinephrine uptake	1.67E-02
internode region of axon	3.32E-02	platelet-derived growth factor binding	3.68E-03	positive regulation of mammary stem cell proliferation	8.41E-03
main axon	2.36E-02	lipoteichoic acid binding	4.94E-02	regulation of developmental process	7.15E-05
9+0 motile cilium	4.13E-02	MHC class II protein binding	4.94E-02	regulation of mammary stem cell proliferation	8.41E-03
kainate selective glutamate receptor complex	4.13E-02	BH3 domain binding	4.94E-02	positive regulation of cell population proliferation	3.34E-04
phagocytic vesicle lumen	4.13E-02	CCR1 chemokine receptor binding	4.94E-02	positive regulation of multicellular organismal process	9.95E-04
endocytic vesicle	3.31E-03	nerve growth factor binding	4.94E-02	regulation of multicellular organismal process	4.36E-05
endocytic vesicle lumen	3.13E-05	poly(A)-specific ribonuclease activity	5.16E-03	negative regulation of dopamine uptake involved in synaptic transmission	8.41E-03
PAR polarity complex	4.94E-02	3'-5'-RNA exonuclease activity	3.49E-02	negative regulation of catecholamine uptake involved in synaptic transmission	8.41E-03
IgE immunoglobulin complex	4.94E-02	RNA exonuclease activity, producing 5'-phosphomonoesters	4.05E-02	regulation of neurotransmitter uptake	1.21E-02
IgD immunoglobulin complex	4.94E-02	RNA exonuclease activity	4.45E-02	negative regulation of neurotransmitter uptake	2.50E-02
platelet dense granule membrane	4.94E-02	phospholipase activator activity	5.99E-04	positive regulation of adenylate cyclase-inhibiting opioid receptor signaling pathway	8.41E-03
immunological synapse	1.45E-07	lipase activator activity	1.04E-03	regulation of adenylate cyclase-inhibiting opioid receptor signaling pathway	8.41E-03
protein complex involved in cell-matrix adhesion	8.79E-03	platelet-derived growth factor receptor binding	5.98E-03	regulation of opioid receptor signaling pathway	2.50E-02

blood microparticle	1.98E-11	long-chain fatty acid-CoA ligase activity	6.87E-03	regulation of G protein-coupled receptor signaling pathway	2.29E-03
lamellipodium membrane	1.45E-02	fatty acid-CoA ligase activity	1.86E-02	regulation of response to stimulus	4.79E-06
cell leading edge	3.11E-02	fatty acid ligase activity	1.86E-02	positive regulation of opioid receptor signaling pathway	8.41E-03
lamellipodium	2.87E-02	acid-thiol ligase activity	2.79E-02	positive regulation of G protein-coupled receptor signaling pathway	4.27E-03
platelet alpha granule lumen	2.51E-04	ligase activity, forming carbon-sulfur bonds	4.65E-02	positive regulation of response to stimulus	2.54E-05
filamentous actin	2.46E-02	CoA-ligase activity	2.15E-02	negative regulation of endodeoxyribonuclease activity	8.41E-03
actin filament	3.00E-03	carbonate dehydratase activity	6.87E-03	negative regulation of deoxyribonuclease activity	1.67E-02
stress fiber	7.55E-04	chemokine activity	3.30E-05	regulation of hydrolase activity	4.65E-03
actomyosin	1.03E-03	cytokine activity	3.63E-03	negative regulation of hydrolase activity	2.60E-02
contractile actin filament bundle	7.55E-04	peroxidase activity	7.42E-06	activation of phospholipase D activity	8.41E-03
actin filament bundle	1.19E-03	oxidoreductase activity, acting on peroxide as acceptor	9.12E-06	positive regulation of phospholipase activity	4.39E-03
heterotrimeric G-protein complex	3.49E-02	antioxidant activity	8.17E-05	regulation of phospholipase activity	4.34E-04
cytoplasmic side of plasma membrane	4.33E-04	ankyrin binding	1.09E-02	regulation of lipase activity	2.65E-03
cytoplasmic side of membrane	1.98E-03	MHC class I protein binding	1.09E-02	positive regulation of lipase activity	1.45E-02
side of membrane	1.54E-08	phospholipase binding	1.58E-02	fast-twitch skeletal muscle fiber contraction	8.41E-03
GTPase complex	4.65E-02	spectrin binding	2.00E-02	twitch skeletal muscle contraction	1.67E-02
tertiary granule lumen	1.11E-02	sphingolipid binding	2.30E-02	voluntary skeletal muscle contraction	1.67E-02

external side of plasma membrane	8.24E-09	coreceptor activity	1.73E-03	positive regulation of erythrocyte enucleation	8.41E-03
cell surface	4.42E-08	structural constituent of cytoskeleton	4.53E-05	regulation of cellular component organization	3.45E-02
Z disc	5.36E-03	transcription corepressor binding	8.10E-03	regulation of erythrocyte enucleation	8.41E-03
I band	8.58E-03	extracellular matrix binding	2.45E-03	regulation of cell differentiation	3.74E-04
sarcomere	3.91E-02	protein kinase C binding	9.54E-03	chemokine (C-C motif) ligand 5 signaling pathway	8.41E-03
myofibril	4.56E-03	protein kinase binding	8.76E-04	response to chemical	5.24E-03
contractile muscle fiber	5.66E-03	kinase binding	3.70E-03	cell surface receptor signaling pathway	5.82E-09
acrosomal vesicle	9.06E-03	dynein intermediate chain binding	3.67E-02	positive regulation of somatic stem cell division	8.41E-03
focal adhesion	2.73E-02	collagen binding	3.18E-03	positive regulation of cell division	9.44E-03
cell-substrate junction	3.07E-02	cyclic nucleotide binding	3.86E-02	regulation of cell division	2.15E-02
anchoring junction	1.38E-02	lipopolysaccharide binding	4.45E-02	regulation of somatic stem cell division	8.41E-03
extracellular exosome	3.51E-03	heme binding	2.10E-04	positive regulation of SNARE complex assembly	8.41E-03
extracellular vesicle	5.31E-03	tetrapyrrole binding	3.18E-04	regulation of organelle organization	3.01E-02
extracellular membrane-bounded organelle	5.32E-03	carbohydrate transmembrane transporter activity	4.65E-02	regulation of vesicle fusion	3.49E-02
extracellular organelle	5.32E-03	structural constituent of muscle	4.86E-02	response to antipsychotic drug	8.41E-03
Unclassified	2.01E-03	antigen binding	4.51E-05	lung ciliated cell differentiation	8.41E-03
nuclear protein-containing complex	1.04E-02	SH3 domain binding	2.89E-03	regulation of branching involved in salivary gland morphogenesis by epithelial-mesenchymal signaling	8.41E-03
nucleus	2.56E-03	heparin binding	6.20E-04	epithelial-mesenchymal cell signaling	4.13E-02

	glycosaminoglycan binding	2.09E-04	cardiac muscle tissue growth involved in heart morphogenesis	8.41E-03
	sulfur compound binding	8.29E-03	growth involved in heart morphogenesis	3.32E-02
	iron ion binding	1.95E-03	muscle structure development	1.29E-02
	phosphoprotein binding	4.37E-02	pH elevation	8.41E-03
	serine-type endopeptidase activity	1.67E-02	regulation of pH	1.23E-02
	serine-type peptidase activity	2.42E-02	monoatomic cation homeostasis	4.18E-03
	serine hydrolase activity	2.61E-02	monoatomic ion homeostasis	4.82E-03
	carbohydrate binding	3.08E-02	homeostatic process	3.55E-02
	identical protein binding	1.34E-02	regulation of nuclear migration along microtubule	8.41E-03
	Unclassified	1.45E-03	positive regulation of hydrogen peroxide catabolic process	1.67E-02
	RNA binding	1.98E-03	positive regulation of hydrogen peroxide metabolic process	4.94E-02
	nucleic acid binding	3.65E-03	regulation of hydrogen peroxide catabolic process	3.32E-02
			negative regulation of mesenchymal cell proliferation involved in lung development	1.67E-02
			negative regulation of developmental process	5.06E-03
			regulation of mesenchymal cell proliferation	3.31E-02
			regulation of mesenchymal cell proliferation involved in lung development	1.67E-02
			positive regulation of oocyte development	1.67E-02
			regulation of oocyte development	2.50E-02
			regulation of oogenesis	2.50E-02

	metanephric podocyte development	1.67E-02	
	metanephric podocyte differentiation	1.67E-02	
	metanephric glomerular epithelial cell differentiation	1.67E-02	
	metanephric glomerular epithelium development	1.67E-02	
	metanephric glomerular epithelial cell development	1.67E-02	
	positive regulation of dipeptide transmembrane transport	1.67E-02	
	positive regulation of dipeptide transport	1.67E-02	
	positive regulation of oligopeptide transport	1.67E-02	
	regulation of oligopeptide transport	1.67E-02	
	regulation of dipeptide transport	1.67E-02	
	regulation of dipeptide transmembrane transport	1.67E-02	
	cytotoxic T cell pyroptotic cell death	1.67E-02	
	pyroptotic cell death	4.13E-02	
	pyroptotic inflammatory response	3.13E-02	
	inflammatory response	3.40E-04	
	nerve growth factor production	1.67E-02	
	neurotrophin production	1.67E-02	
	negative regulation of natural killer cell chemotaxis	1.67E-02	
	regulation of natural killer cell chemotaxis	1.44E-03	

	regulation of lymphocyte chemotaxis	1.72E-02
	regulation of lymphocyte migration	2.04E-02
	regulation of leukocyte migration	1.33E-02
	regulation of cell migration	9.32E-03
	regulation of cell motility	3.87E-03
	regulation of locomotion	3.41E-04
	regulation of immune system process	5.33E-09
	regulation of chemotaxis	3.19E-02
	regulation of response to external stimulus	4.77E-04
	negative regulation of lymphocyte chemotaxis	2.50E-02
	negative regulation of response to stimulus	1.16E-02
	embryonic lung development	1.67E-02
	axon extension involved in regeneration	1.67E-02
	sprouting of injured axon	1.67E-02
	response to wounding	4.37E-08
	platelet maturation	1.67E-02
	positive regulation of myeloid dendritic cell activation	1.67E-02
	positive regulation of leukocyte activation	1.08E-03
	positive regulation of immune system process	3.33E-08

	positive regulation of cell activation	4.81E-06	
	regulation of cell activation	4.62E-07	
	regulation of leukocyte activation	1.21E-04	
	regulation of myeloid dendritic cell activation	4.13E-02	
	positive regulation of somatic stem cell population maintenance	1.67E-02	
	hydroxylysine biosynthetic process	1.67E-02	
	primary metabolic process	9.21E-03	
	hydroxylysine metabolic process	1.67E-02	
	lymphoid lineage cell migration into thymus	1.67E-02	
	lymphoid lineage cell migration	1.67E-02	
	cell migration	7.82E-07	
	cell motility	3.52E-06	
	regulation of postsynaptic neurotransmitter receptor diffusion trapping	1.67E-02	
	regulation of biological quality	4.63E-04	
	nitric oxide transport	4.17E-04	
	negative regulation of phosphatidylinositol biosynthetic process	1.67E-02	
	negative regulation of phospholipid biosynthetic process	4.13E-02	
	negative regulation of phospholipid metabolic process	4.94E-02	
	regulation of phosphate metabolic process	4.80E-02	

regulation of phosphorus metabolic process	4.82E-02
regulation of phosphatidylinositol biosynthetic process	4.13E-02
negative regulation of mitochondrial electron transport, NADH to ubiquinone	1.67E-02
negative regulation of mitochondrial ATP synthesis coupled electron transport	1.67E-02
regulation of mitochondrial ATP synthesis coupled electron transport	4.94E-02
regulation of mitochondrial electron transport, NADH to ubiquinone	4.13E-02
positive regulation of type III interferon production	1.67E-02
positive regulation of cytokine production	3.45E-03
positive regulation of gene expression	7.35E-03
regulation of type III interferon production	4.13E-02
dendritic cell apoptotic process	1.67E-02
oxygen transport	9.19E-10
gas transport	2.77E-10
carbon dioxide transport	9.19E-10
one-carbon compound transport	1.11E-09
positive regulation of cellular pH reduction	6.91E-04
regulation of cellular pH reduction	1.90E-03
regulation of intracellular pH	4.03E-02
regulation of cellular pH	4.49E-02

	intracellular monoatomic cation homeostasis	1.13E-02
	intracellular monoatomic ion homeostasis	1.28E-02
	blood coagulation, intrinsic pathway	3.17E-05
	blood coagulation, fibrin clot formation	8.11E-06
	protein activation cascade	1.69E-05
	blood coagulation	7.63E-09
	coagulation	8.67E-09
	hemostasis	1.11E-08
	regulation of body fluid levels	9.66E-08
	wound healing	2.54E-08
	granzyme-mediated programmed cell death signaling pathway	1.52E-06
	negative regulation of endodermal cell differentiation	2.50E-02
	negative regulation of cell differentiation	1.39E-02
	positive regulation of cell-cell adhesion mediated by integrin	1.03E-03
	regulation of cell-cell adhesion mediated by integrin	3.68E-03
	regulation of cell adhesion mediated by integrin	8.10E-03
	regulation of cell adhesion	3.46E-06
	regulation of cell-cell adhesion	1.10E-03
	positive regulation of cell-cell adhesion	5.33E-04

	positive regulation of cell adhesion	4.29E-06
	positive regulation of cell adhesion mediated by integrin	7.99E-04
	positive regulation of axon guidance	2.50E-02
	positive regulation of respiratory burst	4.72E-05
	regulation of respiratory burst	5.12E-04
	membrane raft polarization	2.50E-02
	membrane raft organization	2.30E-02
	cellular localization	2.19E-03
	maternal aggressive behavior	2.50E-02
	Fc receptor-mediated immune complex endocytosis	2.50E-02
	asparaginyl-tRNA aminoacylation	2.50E-02
	dendritic cell proliferation	2.50E-02
	mononuclear cell proliferation	3.23E-03
	leukocyte proliferation	6.07E-03
	cell population proliferation	9.95E-03
	negative regulation of serotonin uptake	2.50E-02
	regulation of serotonin uptake	4.17E-04
	smooth endoplasmic reticulum calcium ion homeostasis	2.50E-02
	negative regulation of thrombin-activated receptor signaling pathway	2.50E-02

	regulation of thrombin-activated receptor signaling pathway	2.50E-02
	negative regulation of G protein-coupled receptor signaling pathway	1.35E-02
	negative regulation of cell communication	4.91E-02
	establishment or maintenance of actin cytoskeleton polarity	2.50E-02
	cytoskeleton organization	3.56E-03
	actin cytoskeleton organization	7.15E-04
	actin filament-based process	2.12E-03
	positive regulation of metanephric mesenchymal cell migration by platelet-derived growth factor receptor-beta signaling pathway	2.50E-02
	regulation of metanephric mesenchymal cell migration by platelet-derived growth factor receptor-beta signaling pathway	2.50E-02
	cell surface receptor protein tyrosine kinase signaling pathway	2.83E-02
	enzyme-linked receptor protein signaling pathway	4.26E-02
	regulation of metanephric mesenchymal cell migration	3.32E-02
	positive regulation of metanephric mesenchymal cell migration	2.50E-02
	positive regulation of cell migration	2.52E-03
	positive regulation of cell motility	1.28E-03
	positive regulation of locomotion	5.30E-04
	negative regulation of chaperone-mediated autophagy	2.50E-02
	cellular response to water deprivation	2.50E-02

	response to water deprivation	4.13E-02	
	response to oxygen-containing compound	5.74E-03	
	cellular response to water stimulus	4.94E-02	
	cellular response to oxygen-containing compound	2.48E-02	
	cellular response to chemical stimulus	1.57E-03	
	siderophore transport	2.50E-02	
	mesodermal to mesenchymal transition involved in gastrulation	2.50E-02	
	complement-dependent cytotoxicity	2.50E-02	
	cell differentiation involved in salivary gland development	2.50E-02	
	positive regulation of platelet activation	3.25E-06	
	regulation of platelet activation	8.98E-05	
	striatal medium spiny neuron differentiation	3.32E-02	
	head development	4.48E-02	
	principal sensory nucleus of trigeminal nerve development	3.32E-02	
	trigeminal sensory nucleus development	3.32E-02	
	club cell differentiation	3.32E-02	
	aorta smooth muscle tissue morphogenesis	3.32E-02	
	immunoglobulin transcytosis in epithelial cells	3.32E-02	
	negative regulation of epithelial cell differentiation involved in kidney development	3.32E-02	

	T-helper 1 cell lineage commitment	3.32E-02
	CD4-positive, alpha-beta T cell activation	4.25E-04
	positive T cell selection	2.06E-04
	T cell selection	5.06E-05
	adaptive immune response based on somatic recombination of immune receptors built from immunoglobulin superfamily domains	2.84E-06
	adaptive immune response	1.11E-10
	establishment of blood-retinal barrier	3.32E-02
	negative regulation of T-helper 2 cell cytokine production	3.32E-02
	regulation of immune response	3.98E-06
	negative regulation of T cell mediated immunity	2.42E-03
	negative regulation of lymphocyte mediated immunity	1.74E-02
	negative regulation of leukocyte mediated immunity	2.62E-02
	regulation of immune effector process	7.90E-03
	regulation of leukocyte mediated immunity	3.56E-04
	regulation of lymphocyte mediated immunity	4.88E-05
	regulation of adaptive immune response based on somatic recombination of immune receptors built from immunoglobulin superfamily domains	2.87E-02
	regulation of adaptive immune response	3.73E-02
	negative regulation of adaptive immune response based on somatic recombination of immune	1.54E-02

	receptors built from immunoglobulin superfamily domains		
	negative regulation of adaptive immune response	1.89E-02	
	T cell receptor V(D)J recombination	3.32E-02	
	somatic recombination of T cell receptor gene segments	3.32E-02	
	immune system development	3.19E-03	
	somatic diversification of T cell receptor genes	3.32E-02	
	positive regulation of killing of cells of another organism	3.32E-02	
	regulation of killing of cells of another organism	4.13E-02	
	regulation of cell killing	3.81E-05	
	regulation of programmed cell death	2.66E-02	
	positive regulation of cell killing	3.08E-04	
	positive regulation of RNA polymerase II regulatory region sequence-specific DNA binding	3.32E-02	
	regulation of DNA binding	3.18E-03	
	regulation of binding	3.08E-02	
	positive regulation of DNA binding	3.67E-02	
	regulation of acyl-CoA biosynthetic process	1.90E-03	
	regulation of sulfur metabolic process	7.80E-03	
	regulation of ketone metabolic process	2.60E-02	
	negative regulation of low-density lipoprotein receptor activity	3.32E-02	

	negative regulation of glycolytic process through fructose-6-phosphate	3.32E-02
	regulation of glycolytic process through fructose-6-phosphate	3.32E-02
	T cell anergy	3.32E-02
	lymphocyte anergy	3.32E-02
	tolerance induction	1.09E-02
	T cell tolerance induction	4.94E-02
	negative regulation of female receptivity	3.32E-02
	hydrogen peroxide catabolic process	6.48E-08
	hydrogen peroxide metabolic process	9.80E-07
	reactive oxygen species metabolic process	3.81E-05
	CD4-positive, alpha-beta T cell proliferation	2.43E-03
	alpha-beta T cell proliferation	5.98E-03
	T cell proliferation	2.54E-02
	lymphocyte proliferation	1.57E-02
	positive regulation of T cell anergy	4.13E-02
	positive regulation of lymphocyte anergy	4.13E-02
	positive regulation of Fc receptor mediated stimulatory signaling pathway	4.13E-02
	positive regulation of immune response	2.71E-07
	cGMP catabolic process	4.13E-02

	cGMP metabolic process	9.84E-03	
	cyclic purine nucleotide metabolic process	4.65E-02	
	cyclic nucleotide metabolic process	4.86E-02	
	regulation of systemic arterial blood pressure by vasopressin	4.13E-02	
	negative regulation of T cell mediated cytotoxicity	3.02E-03	
	negative regulation of leukocyte mediated cytotoxicity	2.95E-02	
	regulation of leukocyte mediated cytotoxicity	1.90E-04	
	negative regulation of cell killing	3.49E-02	
	negative regulation of cAMP/PKA signal transduction	4.13E-02	
	NK T cell differentiation	4.13E-02	
	positive regulation of dopamine receptor signaling pathway	4.13E-02	
	negative regulation of MHC class II biosynthetic process	4.13E-02	
	transposable element silencing by piRNA-mediated heterochromatin formation	4.13E-02	
	CD27 signaling pathway	4.13E-02	
	positive regulation of CD4-positive, alpha-beta T cell proliferation	3.02E-03	
	positive regulation of alpha-beta T cell proliferation	1.72E-02	
	regulation of T cell proliferation	1.86E-02	
	regulation of lymphocyte proliferation	1.58E-02	
	regulation of mononuclear cell proliferation	4.66E-03	

	regulation of leukocyte proliferation	7.68E-03	
	regulation of lymphocyte activation	1.37E-03	
	regulation of T cell activation	1.76E-03	
	positive regulation of T cell proliferation	1.12E-02	
	positive regulation of T cell activation	6.29E-03	
	positive regulation of leukocyte cell-cell adhesion	1.38E-04	
	regulation of leukocyte cell-cell adhesion	9.57E-05	
	positive regulation of lymphocyte activation	7.30E-03	
	positive regulation of lymphocyte proliferation	3.42E-02	
	positive regulation of mononuclear cell proliferation	8.35E-03	
	positive regulation of leukocyte proliferation	1.29E-02	
	regulation of CD4-positive, alpha-beta T cell proliferation	1.45E-02	
	regulation of CD4-positive, alpha-beta T cell activation	3.39E-02	
	positive regulation of natural killer cell chemotaxis	4.13E-02	
	positive regulation of lymphocyte migration	4.86E-02	
	positive regulation of leukocyte migration	3.96E-02	
	positive regulation of response to external stimulus	3.35E-03	
	apolipoprotein A-I-mediated signaling pathway	4.13E-02	
	nuclear-transcribed mRNA catabolic process, no-go decay	4.13E-02	

	negative regulation of lipoprotein metabolic process	4.13E-02
	tendon development	4.13E-02
	negative regulation of thymocyte apoptotic process	3.02E-03
	negative regulation of T cell apoptotic process	5.31E-05
	negative regulation of lymphocyte apoptotic process	2.85E-04
	regulation of lymphocyte apoptotic process	1.73E-03
	regulation of leukocyte apoptotic process	1.08E-03
	regulation of apoptotic process	3.64E-02
	negative regulation of leukocyte apoptotic process	1.16E-04
	negative regulation of apoptotic process	1.23E-02
	negative regulation of programmed cell death	1.41E-02
	regulation of T cell apoptotic process	4.21E-04
	regulation of thymocyte apoptotic process	6.87E-03
	negative regulation of urine volume	4.13E-02
	renal system process	4.42E-03
	positive regulation of receptor binding	4.13E-02
	axon midline choice point recognition	4.13E-02
	cell recognition	2.93E-02
	miRNA-mediated gene silencing by mRNA destabilization	4.13E-02

	gamma-delta T cell activation	2.10E-05
	negative regulation of skeletal muscle cell proliferation	4.13E-02
	salivary gland cavitation	4.13E-02
	tube lumen cavitation	4.13E-02
	wound healing, spreading of epidermal cells	3.02E-04
	wound healing, spreading of cells	2.21E-03
	epiboly involved in wound healing	2.21E-03
	epiboly	2.42E-03
	morphogenesis of an epithelial sheet	1.06E-02
	regulation of basement membrane organization	3.68E-03
	regulation of extracellular matrix organization	1.60E-02
	positive regulation of isotype switching to IgG isotypes	3.68E-03
	positive regulation of isotype switching	2.46E-02
	positive regulation of immune effector process	7.38E-03
	regulation of isotype switching	4.05E-02
	regulation of immunoglobulin mediated immune response	1.54E-02
	regulation of B cell mediated immunity	1.54E-02
	positive regulation of immunoglobulin mediated immune response	5.63E-03
	positive regulation of B cell mediated immunity	5.63E-03

	positive regulation of lymphocyte mediated immunity	1.28E-04	
	positive regulation of leukocyte mediated immunity	3.31E-04	
	regulation of isotype switching to IgG isotypes	6.87E-03	
	megakaryocyte development	3.77E-05	
	myeloid cell development	3.67E-07	
	myeloid cell differentiation	5.68E-05	
	megakaryocyte differentiation	1.11E-04	
	negative regulation of megakaryocyte differentiation	4.94E-02	
	regulation of hemopoiesis	8.64E-03	
	regulation of megakaryocyte differentiation	1.72E-02	
	positive regulation of establishment of endothelial barrier	4.94E-02	
	positive regulation of endothelial cell development	4.94E-02	
	zygotic determination of anterior/posterior axis, embryo	4.94E-02	
	response to iron(II) ion	4.94E-02	
	positive regulation of integrin-mediated signaling pathway	4.39E-03	
	regulation of integrin-mediated signaling pathway	1.21E-02	
	endodermal cell fate specification	4.94E-02	
	endodermal cell differentiation	6.80E-03	
	endoderm formation	1.17E-02	

	endoderm development	5.29E-03
	formation of primary germ layer	1.92E-02
	cell fate specification	3.92E-02
	negative regulation of transmission of nerve impulse	4.94E-02
	negative thymic T cell selection	4.39E-03
	negative T cell selection	5.16E-03
	thymic T cell selection	6.95E-04
	T cell differentiation in thymus	1.77E-05
	positive thymic T cell selection	4.39E-03
	negative regulation of protein targeting to membrane	4.94E-02
	negative regulation of protein localization to membrane	4.45E-02
	dopamine uptake involved in synaptic transmission	4.94E-02
	catecholamine uptake involved in synaptic transmission	4.94E-02
	establishment of localization in cell	2.36E-02
	cell dedifferentiation	4.94E-02
	dedifferentiation	4.94E-02
	thyroid hormone transport	4.39E-03
	hemidesmosome assembly	4.94E-02
	cell-substrate junction assembly	3.83E-04

	cell-substrate junction organization	4.61E-04	
	cell junction organization	2.95E-02	
	cell junction assembly	1.44E-02	
	positive regulation of prostaglandin biosynthetic process	4.94E-02	
	sperm ejaculation	4.94E-02	
	negative regulation of cardiac muscle contraction	4.94E-02	
	regulation of axon diameter	4.94E-02	
	regulation of anatomical structure size	1.03E-02	
	hemoglobin biosynthetic process	4.94E-02	
	hemoglobin metabolic process	9.17E-05	
	intracellular magnesium ion homeostasis	4.94E-02	
	response to cycloheximide	4.94E-02	
	positive regulation of cytoplasmic mRNA processing body assembly	4.94E-02	
	positive regulation of natural killer cell mediated cytotoxicity directed against tumor cell target	4.94E-02	
	regulation of natural killer cell mediated cytotoxicity	7.48E-05	
	regulation of natural killer cell mediated immunity	9.12E-06	
	regulation of defense response	3.03E-02	
	regulation of response to stress	9.78E-03	
	positive regulation of natural killer cell mediated immunity	9.05E-06	

	positive regulation of innate immune response	4.04E-02	
	positive regulation of defense response	3.91E-03	
	positive regulation of natural killer cell mediated cytotoxicity	8.43E-05	
	positive regulation of leukocyte mediated cytotoxicity	2.45E-03	
	commitment of neuronal cell to specific neuron type in forebrain	4.94E-02	
	type II pneumocyte differentiation	4.94E-02	
	positive regulation of leukocyte tethering or rolling	5.16E-03	
	regulation of leukocyte tethering or rolling	9.84E-03	
	regulation of leukocyte adhesion to vascular endothelial cell	2.21E-03	
	regulation of cellular extravasation	4.65E-02	
	positive regulation of leukocyte adhesion to vascular endothelial cell	1.04E-03	
	immunological synapse formation	5.16E-03	
	negative regulation of peptidyl-threonine phosphorylation	5.16E-03	
	regulation of peptidyl-threonine phosphorylation	2.79E-02	
	glomerular filtration	5.99E-04	
	renal filtration	9.14E-04	
	commissural neuron axon guidance	5.98E-03	
	positive regulation of fibroblast migration	5.98E-03	
	regulation of fibroblast migration	4.05E-02	

	maintenance of cell polarity	5.98E-03
	stress fiber assembly	7.99E-04
	contractile actin filament bundle assembly	7.99E-04
	actin filament bundle assembly	1.88E-04
	actin filament bundle organization	2.34E-04
	actin filament organization	9.29E-03
	supramolecular fiber organization	1.96E-02
	sphingosine-1-phosphate receptor signaling pathway	6.87E-03
	sphingolipid mediated signaling pathway	7.80E-03
	negative regulation of focal adhesion assembly	7.80E-03
	negative regulation of cell-matrix adhesion	3.67E-02
	regulation of cell-substrate adhesion	2.51E-03
	regulation of cell-matrix adhesion	3.59E-03
	negative regulation of cell-substrate junction organization	7.80E-03
	negative regulation of cell junction assembly	2.95E-02
	B cell receptor signaling pathway	4.87E-07
	antigen receptor-mediated signaling pathway	7.56E-11
	immune response-activating cell surface receptor signaling pathway	3.84E-10
	immune response-regulating cell surface receptor signaling pathway	3.15E-10

	immune response-regulating signaling pathway	4.85E-08	
	immune response-activating signaling pathway	1.98E-08	
	activation of immune response	2.43E-07	
	T cell mediated cytotoxicity	8.79E-03	
	T cell mediated immunity	4.59E-03	
	acute inflammatory response to antigenic stimulus	9.84E-03	
	inflammatory response to antigenic stimulus	4.05E-02	
	regulation of glutamate secretion	9.84E-03	
	regulation of amino acid transport	4.86E-02	
	negative regulation of oxidoreductase activity	9.84E-03	
	complement activation, classical pathway	2.85E-04	
	humoral immune response mediated by circulating immunoglobulin	2.94E-05	
	humoral immune response	8.60E-08	
	immunoglobulin mediated immune response	9.54E-04	
	B cell mediated immunity	1.12E-03	
	complement activation	1.26E-03	
	negative regulation of anoikis	1.09E-02	
	regulation of anoikis	2.00E-02	
	positive regulation of T cell receptor signaling pathway	1.09E-02	

	regulation of T cell receptor signaling pathway	9.54E-03
	regulation of antigen receptor-mediated signaling pathway	2.87E-03
	positive regulation of antigen receptor-mediated signaling pathway	2.00E-02
	response to magnesium ion	1.09E-02
	fibrinolysis	1.09E-02
	negative regulation of blood coagulation	5.98E-04
	negative regulation of coagulation	7.61E-04
	regulation of coagulation	4.25E-04
	regulation of blood coagulation	2.88E-04
	regulation of wound healing	1.28E-04
	regulation of response to wounding	5.99E-04
	regulation of hemostasis	3.29E-04
	negative regulation of hemostasis	6.49E-04
	negative regulation of wound healing	2.73E-03
	negative regulation of response to wounding	7.59E-03
	long-chain fatty-acyl-CoA biosynthetic process	1.09E-02
	long-chain fatty-acyl-CoA metabolic process	1.86E-02
	fatty-acyl-CoA metabolic process	4.65E-02
	fatty-acyl-CoA biosynthetic process	2.30E-02

	cellular defense response	6.81E-05
	cell adhesion mediated by integrin	4.21E-04
	cell adhesion	1.41E-07
	erythrocyte development	4.61E-04
	erythrocyte differentiation	2.49E-04
	erythrocyte homeostasis	4.27E-04
	myeloid cell homeostasis	1.34E-03
	homeostasis of number of cells	8.41E-04
	multicellular organismal-level homeostasis	1.19E-02
	T cell costimulation	4.61E-04
	lymphocyte costimulation	5.04E-04
	neutrophil chemotaxis	8.98E-05
	neutrophil migration	2.18E-04
	granulocyte migration	1.12E-04
	myeloid leukocyte migration	8.17E-04
	leukocyte migration	3.73E-05
	granulocyte chemotaxis	4.35E-05
	leukocyte chemotaxis	1.93E-04
	cell chemotaxis	4.13E-04

	chemotaxis	3.03E-05	
	taxis	3.21E-05	
	locomotion	5.73E-05	
	nitric oxide biosynthetic process	1.45E-02	
	nitric oxide metabolic process	2.95E-02	
	reactive nitrogen species metabolic process	3.31E-02	
	stimulatory C-type lectin receptor signaling pathway	1.45E-02	
	cellular response to lectin	1.45E-02	
	response to lectin	1.45E-02	
	platelet formation	1.58E-02	
	platelet morphogenesis	1.86E-02	
	monocyte chemotaxis	1.72E-02	
	mononuclear cell migration	1.53E-02	
	platelet aggregation	7.03E-04	
	platelet activation	1.99E-07	
	homotypic cell-cell adhesion	2.32E-03	
	cell-cell adhesion	2.42E-04	
	lamellipodium assembly	3.40E-03	
	lamellipodium organization	5.49E-04	

	T cell receptor signaling pathway	5.18E-07
	positive regulation of positive chemotaxis	2.00E-02
	regulation of positive chemotaxis	2.15E-02
	natural killer cell activation	2.18E-04
	antibacterial humoral response	2.51E-04
	antimicrobial humoral response	1.75E-07
	defense response to bacterium	6.57E-05
	response to bacterium	4.36E-09
	positive regulation of interleukin-4 production	2.15E-02
	regulation of interleukin-4 production	3.31E-02
	defense response to protozoan	2.15E-02
	response to protozoan	2.62E-02
	negative regulation of miRNA transcription	2.30E-02
	negative regulation of miRNA metabolic process	2.62E-02
	regulation of miRNA metabolic process	3.70E-02
	regulation of miRNA transcription	2.62E-02
	killing of cells of another organism	5.12E-06
	disruption of cell in another organism	5.12E-06
	disruption of anatomical structure in another organism	6.23E-06

	release of sequestered calcium ion into cytosol	1.35E-03
	negative regulation of sequestering of calcium ion	1.44E-03
	regulation of sequestering of calcium ion	8.50E-04
	calcium ion transmembrane import into cytosol	8.49E-03
	regulation of bone resorption	5.63E-03
	regulation of bone remodeling	9.05E-03
	regulation of tissue remodeling	2.54E-02
	negative regulation of stress fiber assembly	2.46E-02
	regulation of actin filament organization	2.69E-02
	regulation of actin cytoskeleton organization	2.96E-03
	regulation of cytoskeleton organization	1.55E-02
	regulation of actin filament-based process	6.25E-03
	negative regulation of actin filament bundle assembly	2.95E-02
	negative regulation of cytoskeleton organization	1.09E-02
	negative regulation of supramolecular fiber organization	1.20E-02
	negative regulation of DNA binding	2.46E-02
	focal adhesion assembly	2.46E-02
	cell-matrix adhesion	1.07E-03
	cell-substrate adhesion	9.54E-04

	regulation of synaptic vesicle recycling	2.46E-02
	cellular oxidant detoxification	1.19E-04
	cellular detoxification	4.68E-04
	detoxification	1.65E-03
	response to toxic substance	1.38E-03
	cellular response to toxic substance	7.23E-04
	positive regulation of viral genome replication	2.79E-02
	regulation of macrophage derived foam cell differentiation	2.79E-02
	positive regulation of smooth muscle contraction	2.79E-02
	integrin-mediated signaling pathway	1.43E-04
	skeletal muscle fiber development	2.95E-02
	myotube cell development	3.67E-02
	muscle cell differentiation	3.18E-02
	striated muscle cell differentiation	3.79E-02
	myotube differentiation	4.05E-03
	positive regulation of vasoconstriction	2.95E-02
	regulation of vasoconstriction	1.96E-02
	blood vessel diameter maintenance	3.96E-02
	regulation of tube diameter	3.96E-02

	regulation of tube size	4.04E-02	
	positive regulation of blood pressure	3.31E-02	
	actin filament capping	3.31E-02	
	negative regulation of actin filament depolymerization	4.05E-02	
	regulation of actin polymerization or depolymerization	3.88E-02	
	regulation of actin filament length	4.12E-02	
	negative regulation of protein polymerization	2.36E-02	
	regulation of protein polymerization	2.98E-02	
	regulation of actin filament polymerization	2.60E-02	
	negative regulation of extrinsic apoptotic signaling pathway in absence of ligand	3.49E-02	
	negative regulation of extrinsic apoptotic signaling pathway	4.85E-02	
	negative regulation of signal transduction in absence of ligand	3.49E-02	
	positive regulation of T cell migration	3.49E-02	
	very long-chain fatty acid metabolic process	3.49E-02	
	positive regulation of fibroblast proliferation	1.01E-02	
	regulation of fibroblast proliferation	3.81E-02	
	Fc-gamma receptor signaling pathway	3.67E-02	
	Fc receptor signaling pathway	1.35E-02	
	positive regulation of interleukin-2 production	3.67E-02	

	regulation of interleukin-2 production	1.81E-02
	nuclear-transcribed mRNA catabolic process, deadenylation-dependent decay	3.67E-02
	astrocyte development	3.86E-02
	astrocyte differentiation	1.96E-02
	glial cell differentiation	3.54E-02
	negative regulation of intracellular steroid hormone receptor signaling pathway	3.86E-02
	positive regulation of establishment of protein localization to mitochondrion	3.86E-02
	positive regulation of cell-matrix adhesion	1.17E-02
	positive regulation of cell-substrate adhesion	4.27E-03
	response to copper ion	4.05E-02
	regulation of lamellipodium assembly	4.05E-02
	positive regulation of type II interferon production	4.05E-03
	regulation of type II interferon production	1.92E-02
	heterotypic cell-cell adhesion	4.25E-02
	maintenance of blood-brain barrier	4.25E-02
	positive regulation of nitric oxide biosynthetic process	4.45E-02
	positive regulation of nitric oxide metabolic process	4.65E-02
	negative chemotaxis	4.45E-02
	spleen development	4.45E-02

hematopoietic or lymphoid organ development	1.08E-02
positive regulation of smooth muscle cell migration	4.45E-02
regulation of smooth muscle cell migration	1.96E-02
antimicrobial humoral immune response mediated by antimicrobial peptide	5.60E-04
regulation of release of cytochrome c from mitochondria	4.65E-02
regulation of substrate adhesion-dependent cell spreading	1.54E-02
Schwann cell development	4.86E-02
positive regulation of wound healing	1.60E-02
positive regulation of response to wounding	2.54E-02
positive regulation of smooth muscle cell proliferation	5.76E-03
regulation of smooth muscle cell proliferation	2.93E-02
cellular response to lipopolysaccharide	2.69E-04
cellular response to molecule of bacterial origin	3.87E-04
response to molecule of bacterial origin	1.56E-04
cellular response to biotic stimulus	8.39E-04
response to lipopolysaccharide	9.47E-05
response to lipid	3.01E-03
cellular response to lipid	1.36E-02
cerebellum development	1.27E-02

	metencephalon development	1.72E-02
	hindbrain development	1.09E-02
	myelination	2.36E-02
	axon ensheathment	2.48E-02
	ensheathment of neurons	2.48E-02
	adenylate cyclase-activating G protein-coupled receptor signaling pathway	1.29E-02
	adenylate cyclase-modulating G protein-coupled receptor signaling pathway	1.93E-02
	positive regulation of phosphatidylinositol 3-kinase/protein kinase B signal transduction	1.98E-02
	positive regulation of inflammatory response	4.04E-02
	regulation of peptidyl-tyrosine phosphorylation	4.20E-02
	regulation of lymphocyte differentiation	3.98E-02
	regulation of leukocyte differentiation	2.31E-02
	regulation of angiogenesis	3.83E-02
	regulation of vasculature development	4.16E-02
	positive regulation of phosphorylation	3.03E-02
	Unclassified	1.11E-02
	protein transport	2.69E-02
	establishment of protein localization	2.69E-02
	protein localization	2.81E-03

	cellular macromolecule localization	2.81E-03
	macromolecule localization	5.38E-04
	intracellular transport	1.40E-02
	protein modification by small protein conjugation	4.81E-02
	protein modification by small protein conjugation or removal	2.42E-02
	post-translational protein modification	1.10E-02
	protein modification process	9.73E-03
	macromolecule modification	3.95E-03
